# Systematic analysis of infectious disease outcomes by age shows lowest severity in school-age children

**DOI:** 10.1038/s41597-020-00668-y

**Published:** 2020-10-15

**Authors:** Judith R. Glynn, Paul A. H Moss

**Affiliations:** 1 Department of Infectious Disease Epidemiology, London School of Hygiene & Tropical Medicine, London, UK; 2 Institute of Immunology and Immunotherapy, College of Medical and Dental Sciences, University of Birmingham, Birmingham, UK

**Keywords:** Viral infection, Bacterial infection, Viral infection, Bacterial infection, Risk factors

## Abstract

The COVID-19 pandemic has ignited interest in age-specific manifestations of infection but surprisingly little is known about relative severity of infectious disease between the extremes of age. In a systematic analysis we identified 142 datasets with information on severity of disease by age for 32 different infectious diseases, 19 viral and 13 bacterial. For almost all infections, school-age children have the least severe disease, and severity starts to rise long before old age. Indeed, for many infections even young adults have more severe disease than children, and dengue was the only infection that was most severe in school-age children. Together with data on vaccine response in children and young adults, the findings suggest peak immune function is reached around 5–14 years of age. Relative immune senescence may begin much earlier than assumed, before accelerating in older age groups. This has major implications for understanding resilience to infection, optimal vaccine scheduling, and appropriate health protection policies across the life course.

## Introduction

A striking feature of the COVID-19 pandemic is that disease and death are both much less common in children than in adults^1^. This unexplained phenomenon has renewed interest in age patterns of disease. Before COVID-19, most contemporary discussion focussed on vulnerabilities at the extremes of age, due to the immature immune system in infants, and immune deterioration or ‘immunosenescence’ in the elderly^2^. There has been little investigation of the comparative response to infection in between theses extremes: indeed, it is generally assumed that the response is broadly constant over this period and that immune function remains stable until around 60 years of age^2^.

Infections which have been noted to increase in severity in young adults, such as Spanish influenza and tuberculosis, are typically discussed as outliers, and explanations sought. However the medical literature a century ago noted that the case fatality rate (deaths/cases) by age is J-shaped for a number of infections, with a ‘honeymoon period’ in school-age children and rising fatality throughout adulthood^3,4^. This pattern, which has never been systematically examined, appears to have been largely forgotten, and the explanation for it is unknown.

We present a systematic analysis of severity of infectious disease by age, concentrating on the age at which severity starts to rise after childhood. We investigated case fatality rates and other measures of severity for a wide range of viral and bacterial infections. We are interested in the effect of age on the outcome of infection, so do not include population mortality rates, as they would be influenced by different exposures to infection by age. For infections for which there are effective treatments or vaccines, older studies were sought, as the interest is in the natural host response. We have included all studies identified by our search that fit our inclusion criteria.

We find that for almost all infections, school-age children have the lowest severity, severity starts to rise well before old age, and for many infections, severity is already higher in young adults than in children.

## Results

**Fig. 1:**
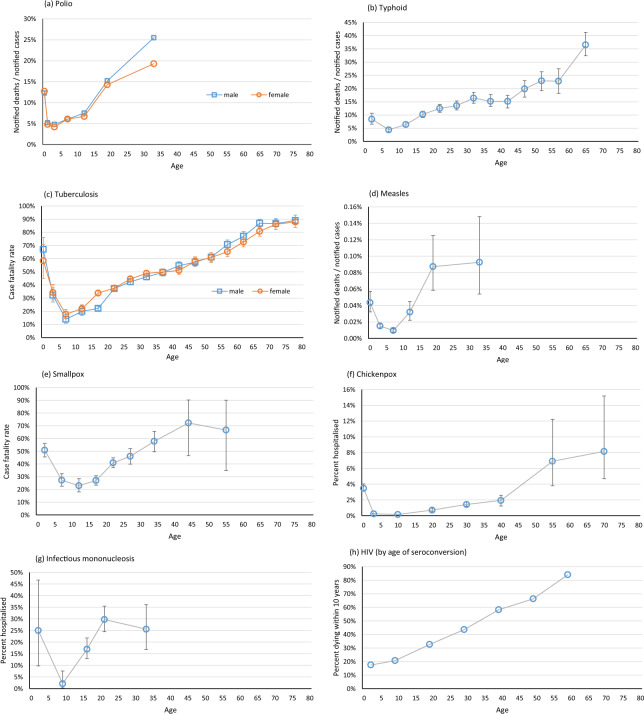
Severity of infectious disease by age for polio, typhoid, tuberculosis, smallpox, chickenpox, measles, infectious mononucleosis and HIV. The 95% confidence intervals on the estimates are shown where they are known or could be calculated. (**a**) Polio in England and Wales 1947–50, notified deaths/notified cases (23,143 cases)^12^; (**b**) Typhoid in small towns in New York State, US, 1915–24, notified deaths/notified cases^13^; (**c**) Pulmonary TB in Denmark, 1925–34, percent of notified cases dying by 31^st^ December 1934^21^; (**d**) Measles in England and Wales, 1970–88, notified deaths/notified cases^29^; (**e**) Smallpox in London Smallpox Hospital, UK, 1836–51, case fatality rate among unvaccinated patients^35^; (**f**) Chickenpox in France 1997–9, estimated hospitalisation rates based on national databases and surveillance^40^; (**g**) Infectious mononucleosis in Rochester, Minnesota, US, 1950–69, percent hospitalised^43^; (**h**) HIV in Europe, North America and Australia, 1983–1996, percent dying within 10 years, mother-to-child infections excluded (12,910 cases, survival estimated from graph)^44^.

**Fig. 2:**
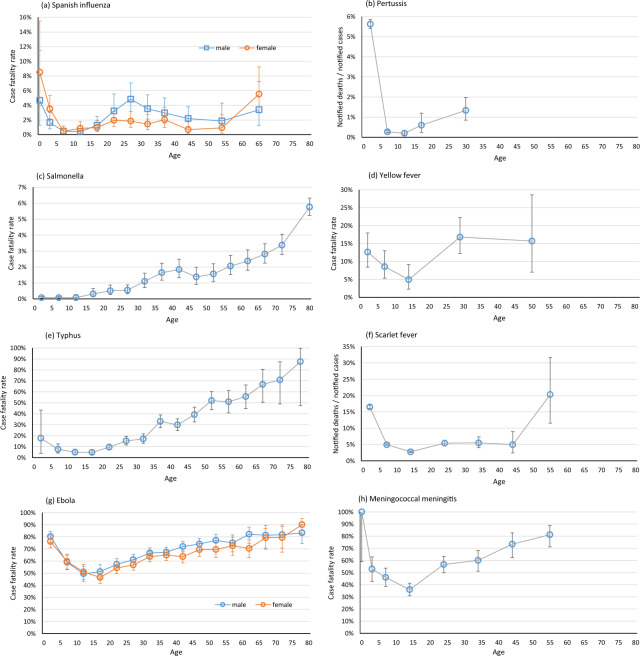
Severity of infectious disease by age for influenza, pertussis, Salmonellosis, yellow fever, typhus, scarlet fever, Ebola, and Meningococcal meningitis. The 95% confidence intervals on the estimates are shown where they are known or could be calculated. (**a**) Influenza in Maryland, US, 1918–19, case fatality rate from household surveys^47^; (**b**) Pertussis in small towns in New York State, US, 1915–24, notified deaths/notified cases^13^; (**c**) Salmonellosis in Spain 1997–2006, case fatality rate among hospitalised patients (numbers estimated from graph and population data)^55^; (**d**) Yellow fever in New Orleans, US, 1878, case fatality rate^63^; (**e**) Typhus in London Fever Hospital 1848–57, case fatality rate^15^; (**f**) Scarlet fever in Pennsylvania, US, 1907–12, notified deaths/notified cases^9^; (**g**) Ebola in Guinea, Liberia and Sierra Leone, 2013–15. case fatality rate^66^; (**h**) Meningococcal meningitis in Cyprus, 1908–9, case fatality rate^70^.

**Fig. 3:**
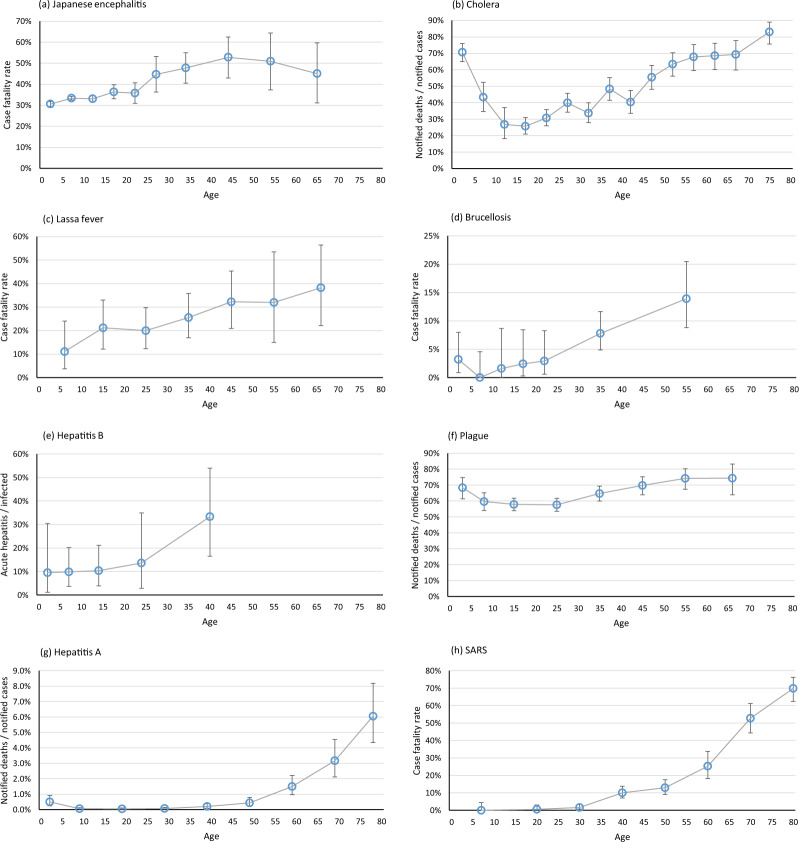
Severity of infectious disease by age for Japanese encephalitis, cholera, Lassa fever, brucellosis, hepatitis B, plague, hepatitis A, and SARS The 95% confidence intervals on the estimates are shown where they are known or could be calculated. (**a**) Japanese encephalitis in Korea, 1955–66, notified deaths/notified cases^73^; (**b**) Cholera in Munich, Germany, 1873–4, notified deaths/notified cases^76^; (**c**) Lassa fever in Nigeria, 2018, case fatality rate in laboratory confirmed cases^91^; (**d**) Brucellosis in Malta, 1936, case fatality rate^96^; (**e**) Hepatitis B in Alaska, 1971–6, proportion of new infections with symptomatic hepatitis^99^; (**f**) Plague in 75 villages in Jalandhar District, India, 1897–8, notified deaths/notified cases^102^; (**g**) Hepatitis A in England and Wales, 1979–85, notified deaths/notified cases^111^; (**h**) SARS in Hong Kong, 2003, case fatality rate (numbers estimated from incidence and population data)^114^.

**Fig. 4:**
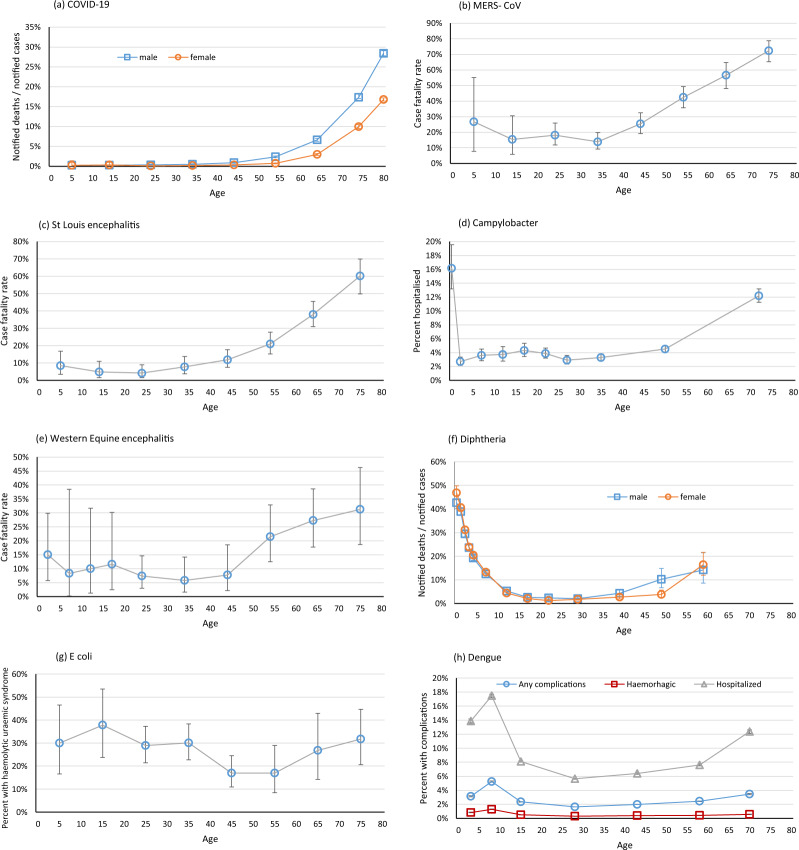
Severity of infectious disease by age for COVID-19, MERS-CoV, St Louis encephalitis, Western equine encephalitis, diphtheria, Escherichia coli, and dengue. The 95% confidence intervals on the estimates are shown where they are known or could be calculated. (**a**) COVID-19 in Spain 2020, notified deaths/notified confirmed cases, as of 11 May 2020^117^; (**b**) MERS-CoV in Saudi Arabia June 2012-July 2014 and 2017–18, case fatality rate^124,125^; (**c**) S Louis encephalitis in St Louis, US, 1933, case fatality rate^126^, (**d**) Campylobacter in Canada, 2001–4, percent hospitalised^57^; (**e**) Western equine encephalitis in Mannitoba, Canada, 1941, case fatality rate^134^; (**f**) Diphtheria in London, UK, 1894–1903, notified deaths/notified cases^135^; (**g**) *E coli* 0104/H4 Hamburg, Germany, 2011, proportion developing haemolytic uraemic syndrome^138^; (**h**) Dengue in Brazil 2000–2014, proportion haemorrhagic, with any complications, and hospitalised^142^.

Information on at least one measure of severity in at least one study was found for 32 infections. Severity is measured as case fatality rate, or the proportion of cases with severe disease (e.g. the proportion hospitalised), or as the proportion with symptomatic disease among those with infection or known exposure. For ease of comparison, a figure from the largest dataset for each infection is presented in Figs. 1–4. Details of all identified studies for each infection are given in the Supplementary Material^1,5–143^, together with figures, including the number of cases in each age group, where known, and 95% confidence intervals where these could be calculated. Results are shown separately by sex when this was available and the sample size was sufficient.

For most infections clinical severity is relatively high in infancy, drops in early childhood and rises in adulthood and more steeply into old age. Figures 1–4, Supplementary Figures S1–32 and online-only Table 1 are arranged in order of the age at which the severity starts to rise.

For many infections the rise in severity is apparent by age 20 years. For polio, typhoid, tuberculosis and measles (Fig. 1a–d, Supplementary Figures S1–4) some datasets suggest that the case fatality rate is already higher by age 10–14 years than in younger children^9,11–13,21,29^. A rise in severity by age 15–19 or 15–24 years is seen clearly in large datasets for case fatality rates for smallpox^35^, HIV^44^, Spanish influenza^47,48^, pertussis^13^, and Salmonella^55^, for hospitalisation rates for chickenpox^40^, and in smaller datasets for hospitalisation for infectious mononucleosis^43^, and case fatality rate for Yellow fever^63^ (Figs. 1d–h, 2a–d, Supplementary Figures S5–12).

A slightly later rise, from around 20 years, is seen for case fatality rates for typhus^15^, scarlet fever^9^, Ebola^66^, meningococcal meningitis^70^, and Japanese encephalitis^73^ (Figs. 2e–h, 3a, Supplementary Figures S13–17). For cholera, case fatality rates were lowest around 10-24 years in a number of datasets (Supplementary Figure S18)^75,76^, but mortality rates in the large 19^th^ century waterborne outbreaks, in which all age groups would have been exposed, were lowest at 5-14 years^88,89^. For Lassa fever, case fatality rates in Nigeria^91,92^ increased with age from 20–30 years but no particular pattern with age was seen in Sierra Leone (Supplementary Figure S19)^93,94^.

For most of the remaining infections for which information was available, the severity rises with age from 30 years (seasonal influenza^48^, brucellosis^96^, plague^102^, probably hepatitis B acute infection^99^, Fig. 3d–f, Supplementary Figures S9c, S20–22), 40 years (Hepatitis A^111^, SARS^114^, COVID-19^117^, MERS-CoV^124,125^, St Louis encephalitis^126^, Figs. 3g,h4a–c, Supplementary Figures S23–27) or 50 years (Campylobacter^57^, Western equine encephalitis^134^, Fig. 4c,d Supplementary Figures S28, 29).

Three infections show different patterns, with greater severity in children than in young adults. The case fatality rate from diphtheria drops steeply with age in childhood, and continues to fall during adolescence, so the lowest case fatality rate is in the 20 s and 30 s, before rising from age 40 years (Fig. 4f, Supplementary Figure S30)^9,135^. The proportion of people with Shiga-toxin producing *E coli* who develop the haemolytic uraemic syndrome follows a similar pattern, with a drop during childhood, and the lowest proportion in the 40 s and 50 s before rising from age 60 years (Fig. 4g, Supplementary Figure S31)^138,139^. For Dengue the proportion with more severe disease (measured as the proportion with haemorrhagic or any complications or the proportion hospitalised) is greatest in school-age children, dropping to a nadir in the 20–30 s before rising again (Fig. 4h, Supplementary Figure S32)^142^.

## Discussion

Our findings show that the severity of most infectious diseases is at its lowest in school-age children. Strikingly, the severity was higher among young adults than among school-age children for polio^12^, typhoid^13–15^, tuberculosis^21,22^, measles^29^, smallpox^34,35^, chickenpox^40,41^, infectious mononucleosis^43^, HIV^44^, influenza^47,48^, pertussis^13^, Salmonella^55^, Yellow fever^62,63^, typhus^15^, scarlet fever^9,65^, Ebola^144^, meningococcal meningitis^70^, Japanese encephalitis^73^ cholera^76^, and perhaps Lassa fever^91^. Some infections show a slower rise of severity with age after childhood (brucellosis^96^, plague^102^, coronavirus infections^113,115,117,124,125^, acute hepatitis B^99^ and hepatitis A^111^, St Louis encephalitis^126^, Campylobacter^57^, and Western equine encephalitis^134^,) but for most this rise begins well before old age. For Diphtheria^9,135^ and Shiga toxin-producing E coli^138,139^ severity remains raised in childhood and adolescents compared to young or middle-age adults, and for Dengue^142^ the greatest severity is in school-age children.

Since exposure may vary for children and adults we examined case fatality rates and other measures of severity, not mortality rates. Many reports relied on clinical diagnoses or were based on surveillance data or were limited to hospitalised patients, and it is possible that diagnoses, notifications and hospitalisations differ for children and adults. In order to measure outcomes that are unaffected by vaccination or effective treatments, some of the included studies are old. But even some older studies were carefully conducted. House-to-house surveys of smallpox^34^ and influenza^47,48^ ensured mild cases and all ages are included. Direct estimates of fatalities in well-characterized outbreaks of Yellow fever^62,63^, meningococcal meningitis^70^, and viral encephalitides^73,126^ avoid any strain differences or notification differences by age. Long-term follow-up studies of patients with tuberculosis^21,22^ ensured all deaths are included. For each infection the different datasets tell a consistent story. The general pattern is remarkable, and unlikely to be explained by biases in the data.

A number of factors may explain these observations. The severity of infectious disease depends on the virulence of the infecting organism, the dose or route of infection, and the response of the host. Variation in strain virulence by age is unlikely, and many of the studies reported outbreaks of single strains (e.g. Spanish influenza, Ebola, smallpox, typhoid, cholera). Most infections have a single route or mode of infection, and for HIV the mode of infection does not explain the age pattern^44^.

Adolescents and adults may be exposed to higher doses of infectious agents than are younger children, through caring responsibilities or because they eat more. Greater exposure often increases the risk of acquiring an infection, but the relationship with disease severity is less consistent. An association between infecting dose and severity of disease has been suggested for measles^145^ and perhaps chickenpox^146,147^, and for Salmonella food-poisoning but not typhoid^148–150^. Fatal cases of SARS and MERS had a slightly shorter incubation period, consistent with a higher infectious dose, but this was not found for influenza^151–153^, and an association between severity and incubation period could be explained by host susceptibility. For Ebola, infecting dose (as estimated by degree of exposure to people with Ebola virus disease and their bodily fluids) is strongly associated with the risk of disease, but did not influence the case fatality rate^67,69^. Infectious dose cannot explain the continued rise in severity throughout adulthood.

The J-shaped pattern of severity, high in infancy, dropping in early childhood to a minimum around age 10 years, and rising in adolescence and through adulthood, with a steep increment in old age mirrors that seen for all-cause mortality across populations^154,155^. It is also seen for many of the more frequent causes of death including respiratory disease, diarrhoeal disease, and tuberculosis^154^, consistent with a changing age-specific host response.

Co-morbidities tend to increase with age but are generally low in young adults. Interestingly, in a large study of trauma in the US, the case fatality rate, adjusted for severity of injury, only rose from age 55 years onwards^156^, suggesting that the change in resilience to infection at younger ages is immunological, not dependent only on co-morbidities or physiological changes.

Further support comes from the immunological response to vaccine challenge by age. In clinical trials of human papilloma virus vaccine, the antibody levels to 9 different component vaccine sub-types measured 7 months post-vaccination decreased with each year increase in age from 9–26 years, with the steepest decline between ages 10 and 16 years^157^. A similar profile was seen in responses to Hepatitis B vaccine in China, with antibody titres in subjects vaccinated at ages 15–24 years half of those vaccinated at 5–14 years;^158^ and in bacteriocidal antibody responses to Meningococcal group A conjugate vaccine in West Africa, with lower titres in those aged 18–29 years at vaccination than in those aged 2–10 or 11–17 years^159^. However this is not universal: responses by age to the Meningococcal B vaccine varied by vaccine strain^160^.

Humoral responses to natural infection also indicate that immune function may be optimised in children and adolescents. Antibody titres against persistent herpesviruses such as cytomegalovirus and varicella zoster increase in response to recurrent episodes of viral reactivation so higher titres indicate less effective immune control, or re-infection. In the Gambia, where most people are infected with cytomegalovirus in infancy, cytomegalovirus-specific antibody levels show a ‘J-shaped’ pattern with age, with the lowest levels (suggesting optimal control) in adolescents, rising after age 20 years^161^. Similarly, Varicella zoster is ubiquitous where there is no vaccination, and studies in different settings suggest that antibody levels in seropositive individuals are at their lowest (suggesting optimal control) at 8–9 or 15–19 years^162,163^.

The immune system changes in infancy and old age are well described^2^. After birth the weak innate immune system of neonates strengthens rapidly, and the adaptive immune system develops an expanded repertoire of memory B and T cells triggered by infections (and vaccinations), the microbiome, and allergens in food and the environment^2^. With ageing, there is a decline in naïve T-cell numbers and T-cell receptor diversity, cytokine production by CD4 and CD8 T-cells is impaired, the CD4 to CD8 ratio is inverted, and the cells of the innate immune response function less well^164^. It is likely that the majority of the data presented here reflect the response to initial pathogen exposure, as those who are already fully protected by acquired immunity will not be included as cases, and those who are partially protected may have mild disease and not be diagnosed. The primary immune response may be particularly affected by ageing. The very high case fatality rates in elderly people observed in response to novel pathogens such as COVID-19, MERS, SARS, St Louis encephalitis, and Ebola, may result from restriction of the naïve T-cell repertoire.

Studies of immune senescence have focussed largely on those over 60 years, but our data indicate that important changes in immune function are apparent at a much younger age. This could be explained by relative ‘senescence’ of the immune system starting in young adulthood or even earlier^165,166^. Peak immune function in childhood and adolescence, at around age 5–14 years, might represent the intersection of improved function due to maturity and increasing antigenic exposure, and decreasing function due to early senescence. This is supported by assessment of immune parameters, some of which change almost linearly over the lifespan^167^, and by changes in the thymic cortex and medulla which peak in size at age 4–11 years^168^, or earlier^169^.

The severity of infectious disease depends not only on the control of the infectious agent but also on pathological damage arising from the immune response. Dengue is an example where strong immune responses in childhood are associated with increased clinical complications due to antibody dependent enhancement^170^. It has been suggested that the balance between an insufficient immune response and an over-active immunopathological response is generally more favourable in children^4^. However it seems surprising that a similar balance would be required for the wide range of viral and bacterial infections with similar age-severity patterns presented here, including infections in which protection is mainly antibody-mediated (e.g. smallpox) and those in which it is predominantly cell-mediated (e.g. tuberculosis).

The increase in infectious disease severity during adolescence suggests that puberty could have a role. Sex hormones influence immune responses (reviewed in^171,172^) but in different ways and with inconsistent findings between studies. Overall, testosterone tends to suppress and oestrogens to enhance the immune response^172,173^. These associations may contribute to the generally higher mortality rate from infectious disease in males and higher incidence of autoimmune disease in females^173,174^. Numerous differences in immune marker concentrations and response to infection have been described between males and females, and these increase after puberty^175^. However, where data are available, the increase in severity of disease in young adults compared to children and adolescents was seen in both males and females (e.g. Ebola, tuberculosis, Spanish influenza, polio). The decreased immune response to human papilloma virus vaccine with age, and the profile of cytomegalovirus-specific immunity with age, are also similar in males and females^157,161,176^.

Environmental exposures and heterologous infections may modulate the magnitude and quality of the immune response at different ages. For tuberculosis it has been argued that not only HIV but some (unknown) sexually and parenterally transmitted virus might trigger progression to disease and explain the age distribution^177^.

Persistent infections, such as herpesviruses, may establish early in life, or increase with age, especially with the changing social contact patterns of adolescence. Cytomegalovirus seropositivity has been associated with immunosenescent changes, including a reduction in the naïve T-cell pool and commensurate increase in CD8 + memory T-cells with a reduced CD4:CD8 ratio^165,166,178–181^, as well as with increased risk of mortality in older age^182,183^. However, the functional consequence of persistent cytomegalovirus infection appears to vary with age and cytomegalovirus may enhance responses to heterologous infections in younger people^166,184–186^. In most countries (and probably everywhere at the time when some of the data we present were collected) almost everyone is infected with cytomegalovirus in early childhood^161,187^, and continuing exposure and multiple infections are likely. So if cytomegalovirus has a role in explaining the age-pattern of severity, it may reflect immunological attrition due to the burden of persistent infection or reinfection rather than simple presence or absence of infection^188^.

The age pattern of severity suggests that natural selection has acted to optimise immune function in childhood and adolescence with a subsequent decline during adulthood. Peak function before reproduction seems counter-intuitive, but according to life history theory there is a trade-off between the energy required for immune response and that required for other functions, such as reproduction and growth^189,190^. Whilst the energy costs of innate immunity are spread throughout the lifetime^189,190^ the adaptive immune system has high upfront but lower running energy costs. Thymic involution conserves energy when sufficient naïve T-cells have been generated^189^, facilitating the metabolic demands of the adolescent growth spurt and secondary sexual development^191^, but leaving the elderly more vulnerable.

SARS-CoV and SARS-CoV-2 appear to have more extreme variation in severity by age than other infections, with predominantly mild disease in children, and very high case fatality rates in the elderly. Unusually, there is no evidence of higher severity in infants. The reasons for this extreme variation are not yet known, but should be considered in the context of the findings of this review.

Appreciating how responses to infections vary by age outside the extremes of age can have major implications. Understanding the prevalence and transmission of SARS-CoV-2 in children, and how this differs from influenza, is crucial in predicting the role of school closures in the response^192^. Vaccination programmes that increase the average age of infection, can paradoxically increase the numbers of people with severe disease^193^. This has led to targeted vaccination of older children for measles and rubella. The higher vaccine response of younger children to human papilloma virus vaccine may lead to a shift in target age groups.

The finding that many infectious diseases are least severe in school-age children suggests that relative immune ageing starts far earlier than has previously been recognised. Improved understanding of the mechanisms underlying this observation may provide novel opportunities for intervention strategies. Children tend to have lower mortality than adults from sepsis, and comparison of their responses is being used in drug discovery^194^; similar approaches could be used for other infections. Comparison of immune response to infections which increase in severity in young adults and those that only increase later may give clues to drug and vaccine design. In the midst of a pandemic and with increasing antimicrobial resistance, new approaches such as these are desperately needed.

## Methods

We sought information on severity of infectious disease by age for a wide range of different infections.

### Defining outcome measures

The focus of this study is the host response to infection by age. We are interested in the outcome of infections or exposure. We have therefore used:

- Case fatality rates estimated directly from cohorts of individuals
- Case fatality rates estimated from surveillance data, comparing numbers of deaths and numbers of cases
- Proportion of cases with severe disease (e.g. the proportion hospitalised)
- Proportion with symptomatic disease among those with infection or known exposure

Note that mortality rates, though more readily available, would be misleading, as exposure is likely to vary by age.

### Sources of data and possible biases

The most accurate data come from situations in which there is active case ascertainment to ensure mild cases are not missed, and active follow-up to ensure outcomes are recorded, ideally proving infection through serology or microbiology. But such studies are rare, and imposing such strict criteria would leave very little data.

Hospital-based data exclude mild cases, and admission criteria may vary with age, but outcomes are likely to be accurately recorded.

Surveillance data provide some of the largest studies but interpreting the age patterns assumes diagnosis and notification are not influenced by age group. Case fatality rates measured from surveillance data can also be biased because of the lag between incidence and death. This is a particular problem in ongoing epidemics, and for diseases with long duration, but will not necessarily bias the age pattern.

Strains of different virulence could affect different sections of the population. Although it is unlikely that strains would differ systematically by age group, studies of single outbreaks of disease avoid this theoretical problem, but are only available for some infections.

For infections for which effective vaccines or treatments are available there may be very few cases or few severe cases, and differential access to or efficacy of vaccines and treatment by age could influence the results. For these infections estimates from the older (e.g. pre-antibiotic or pre-vaccine) literature can be helpful.

For endemic infections leading to life-long immunity, disease in adults may be rare or atypical, so estimates from isolated (e.g. island) populations are most informative. Similarly, newly-emerged infections, or infections in areas where they have not been seen before, will not be affected by partial immunity in older individuals.

### Search strategy

Finding sufficiently detailed age-specific information for different diseases is not straightforward and a conventional systematic review is not possible. Very few studies are done to look at the effect of age on disease outcomes. Most outbreak or surveillance reports do not include enough detail, giving age only as a median and range, or giving the age breakdown only for deaths or only for cases. Relevant data are occasionally included as background information in studies on other topics.

Information on different infections was found in different ways. PubMed was used to find reports on specific infections, searching with “case fatality” or “severity” or “hospital*”and “age”, or looking at reports of large outbreaks. Outbreak reports were also searched through the *Morbidity and Mortality Weekly Report (MMWR)* produced by the US Centers for Disease Control.

To find suitable studies in the older literature, the journal *Public Health* was hand-searched from volume 1 (1888) to volume 59 (1946), the *Public Health Bulletin* from 1904–1949 and the issues of the *Journal of Tropical Medicine and Hygiene* available on-line (1915–19, 1921, 1923). All pamphlets and reports in the London School of Hygiene & Tropical Medicine library open shelves and books in the Wellcome collection library open shelves relating to infectious diseases were hand-searched. These include reports from the 19^th^ century onwards. We also searched books and reports available electronically from the United States Public Health Service, US National Library of Medicine Digital Collections, the London School of Hygiene & Tropical Medicine, or Google on plague, cholera and yellow fever. The authors’ literature collection was searched, and relevant references followed (snowball searching).

All identified studies that gave information on the age-pattern of severity were included if they satisfied the following criteria:

- Included children and adults
- Data stratified in 10-year age groups or less
- At least 100 cases included
- Source of data given

All eligible studies were included whatever age-severity pattern they showed. Where the number of cases and outcomes was presented, or could be derived, by age group, binomial exact 95% confidence intervals were calculated for the proportions. For each infection, the relative risk of the outcome (death or other measure of severity) in adults was compared to that in children using the largest dataset with information on numbers of individuals in the relevant age groups.

## Supplementary information

Supplementary information

## Data Availability

All datasets are available in an Excel file^195^. This links to the supplementary material, and includes the origin of each dataset.

## Online-only Table

**Online-only Table 1:** Comparison of disease severity in children and adults.

Disease	Study	Outcome	Age groups	% with outcome		Relative risk (95% CI)	*p*
				Child	Adult	Adult/Child	
Polio	New York 1916^9^	Death	5–14 vs 20–29 10–14 vs 20–29	25.9 28.0	35.6 35.6	1.38 (1.00–1.89) 1.27 (0.88–1.84)	0.064 0.22
Typhoid	New York 1915–24^13^	Death	5–14 vs 20–29 10–14 vs 20–29	5.4 6.4	12.9 12.9	2.38 (2.05–2.75) 2.01 (1.68–2.39)	<0.0001 <0.0001
Tuberculosis	Denmark 1925–34^21^	Death	5–14 vs 20–29 10–14 vs 20–29	19.1 21.1	40.2 40.2	2.11 (1.94–2.29) 1.90 (1.72–2.10)	<0.0001 <0.0001
Measles	England & Wales 1970–88^29^	Death	5–14 vs 15–24 10–14 vs 15–24	0.012 0.032	0.087 0.087	7.34 (4.89–11.01) 2.73 (1.66–4.49)	<0.0001 <0.0001
Smallpox	London 1836–51^35^	Death	5–14 vs 20–29 10–14 vs 20–29	25.3 23.0	42.4 42.4	1.67 (1.43–1.96) 1.85 (1.47–2.33)	<0.0001 <0.0001
Chickenpox	France 1997–9^40^	Hospitalised	5–14 vs 15–24	0.16	0.71	4.5	<0.0001
Infectious mononucleosis	Minnesota 1950–69^43^	Hospitalised	5–14 vs 19–24	2.2	29.7	13.8 (3.47–55.1)	<0.0001
HIV	Multisite 1983–96^44^	Death in ≤10 years	5–14 vs 15–24	20.7	32.6	1.58 (1.38–1.81)	<0.0001
Influenza	Maryland 1918–19^47^	Death	5–14 vs 20–29 10–14 vs 20–29	0.55 0.65	2.7 2.7	4.94 (2.94–8.30) 4.18 (2.16–8.11)	<0.0001 <0.0001
Pertussis	New York 1915–24^13^	Death	5–14 vs 15–19 10–14 vs 15–19	0.26 0.20	0.58 0.58	2.24 (1.04–4.79) 2.89 (1.18–7.06)	0.034 0.015
Salmonella	Spain 1997–2006^55^	Death	5–14 vs 20–29 10–14 vs 20–29	0.081 0.081	0.55 0.55	6.77 (3.31–13.8) 6.77 (1.62–28.3)	<0.0001 0.0023
Yellow Fever	Kentucky 1878^62^	Death	5–14 vs 20–29 10–14 vs 20–29	31.0 28.0	51.0 51.0	1.65 (0.97–2.80) 1.82 (0.92–3.62)	0.053 0.059
Typhus	London 1848–57^15^	Death	5–14 vs 20–29 10–14 vs 20–29	5.9 5.0	11.8 11.8	2.02 (1.37–2.96) 2.38 (1.46–3.88)	0.0002 0.0002
Scarlet fever	Pennsylvania 1907–12^9^	Death	5–19 vs 20–29 10–19 vs 20–29	4.2 2.8	5.4 5.4	1.30 (1.11–1.53) 1.96 (1.64–2.34)	0.0011 <0.0001
Ebola	W Africa 2013–15^66^	Death	5–14 vs 20–29 10–14 vs 20–29	57.8 53.2	59.8 59.8	1.03 (0.97–1.10) 1.12 (1.03–1.23)	0.29 0.007
Meningococcal meningitis	Cyprus 1908–9^70^	Death	5–19 vs 20–29 10–19 vs 20–29	39.4 35.9	56.7 56.7	1.43 (1.23–1.68) 1.58 (1.32–1.89)	<0.0001 <0.0001
Japanese encephalitis	Korea 1955–66^73^	Death	5–14 vs 20–29 10–14 vs 20–29	33.3 33.1	38.1 38.1	1.14 (1.02–1.28) 1.15 (1.02–1.30)	0.022 0.024
Cholera	Munich 1873–4^76^	Death	5–14 vs 20–29 5–14 vs 30–39 10–14 vs 20–29 10–14 vs 30–39	36.5 36.5 26.9 26.9	34.9 40.5 34.9 40.5	0.96 (0.78–1.17) 1.11 ((0.90–1.36) 1.30 (0.91–1.84) 1.51 (1.06–2.14)	0.66 0.32 0.13 0.01
Lassa fever	Nigeria 2018^91^	Death	0–10 vs 21–30 0–10 vs 31–40 0–20 vs 21–30 0–20 vs 31–40	11.1 11.1 17.1 17.1	20.0 25.6 20.0 25.6	1.8 (0.71–4.53) 2.3 (0.94–5.65) 1.17 (0.65–2.09) 1.49 (0.87–2.56)	0.19 0.05 0.60 0.14
Brucellosis	Malta 1936^96^	Death	5–14 vs 20–24 5–14 vs 25–44 10–14 vs 20–24 10–14 vs 25–44	0.71 0.71 1.6 1.6	2.9 7.8 2.9 7.8	4.11 (0.43–38.92) 11.0 (1.49–80.69) 1.81 (0.19–16.98) 4.82 (0.66–35.17)	0.31 0.0025 1.0 0.092
Hepatitis B	Alaska 1971–6^99^	Symptomatic acute	5–19 vs 20–29 5–19 vs 30+ 10–19 vs 20–29 10–19 vs 30+	10.1 10.1 10.3 10.3	13.6 33.3 13.6 33.3	1.35 (0.42–4.40) 3.31 (1.55–7.04) 1.32 (0.36–4.82) 3.22 (1.27–8.14)	0.62 0.0019 0.68 0.0096
Plague	India 1897–8^102^	Death	6–20 vs 21–30 6–20 vs 31–40 11–20 vs 21–30 11–20 vs 31–40	58.5 58.5 57.9 57.9	57.6 64.8 57.6 64.8	0.98 (0.90–1.07) 1.11 (1.01–1.21) 0.99 (0.90–1.09) 1.12 (1.02–1.23)	0.73 0.028 0.92 0.025
Hepatitis A	England & Wales 1978–85^111^	Death	5–14 vs 15–24 5–14 vs 25–34 5–14 vs 35–44	0.060 0.060 0.060	0.043 0.065 0.19	0.72 (0.24–2.20) 1.09 (0.38–3.13) 3.24 (1.22–8.62)	0.56 0.88 0.013
SARS	Hong Kong 2003^114^	Death	0–14 vs 15–24 0–14 vs 25–34 0–14 vs 35–44	0.0 0.0 0.0	0.56 1.62 10.0	— — -	0.69 0.60 0.001
COVID-19	Spain 2020^117^	Death	10–19 vs 20–29 10–19 vs 30–39 10–19 vs 40–49 10–19 vs 50–59	0.31 0.31 0.31 0.31	0.17 0.27 0.57 1.43	0.55 (0.21–1.44) 0.87 (0.35–2.15) 1.81 (0.75–4.39) 4.54 (1.89–10.9)	0.22 0.76 0.18 0.0002
MERS	Saudi Arabia 2012–18^124,125^	Death	10–19 vs 20–29 10–19 vs 30–39 10–19 vs 40–49 10–19 vs 50–59	15.4 15.4 15.4 15.4	18.1 13.9 25.4 34.2	1.18 (0.52–2.68) 0.90 (0.40–2.05) 1.65 (0.76–3.60) 2.22 (1.00–4.93)	0.70 0.81 0.18 0.032
St Louis encephalitis	St Louis US 1933^126,130^	Death	10–19 vs 20–29 10–19 vs 30–39 10–19 vs 40–49	4.9 4.9 4.9	4.2 7.8 11.9	0.87 (0.27–2.77) 1.60 (0.56–4.52) 2.46 (0.96–6.32)	0.81 0.37 0.050
Campylobacter	Canada 2001–4^57^	Hospitalised	5–14 vs 20–29 5–14 vs 30–39 5–14 vs 40–59	3.7 3.7 3.7	3.4 3.3 4.5	0.93 (0.75–1.16) 0.90 (0.72–1.13) 1.23 (1.01–1.51)	0.52 0.38 0.038
Western equine encephalitis	Manitoba Canada 1941^134^	Death	5–14 vs 20–29 5–14 vs 30–39 5–14 vs 40–49 5–14 vs 50–59 5–14 vs 60–69	9.4 9.4 9.4 9.4 9.4	7.4 5.8 7.7 21.4 27.3	0.79 (0.22–2.86) 0.62 (0.15–2.60) 0.82 (0.20–3.43) 2.29 (0.71–7.34) 2.91 (0.93–9.07)	0.73 0.51 0.79 0.14 0.04
Diphtheria	London 1894–1903^135^	Death	5–14 vs 20–24 10–14 vs 20–24	10.7 4.8	1.6 1.6	0.15 (0.12–0.19) 0.34 (0.27–0.43)	<0.0001 <0.0001
Escherichia coli	Hamburg 2011^138^	Haemolytic uraemic syndrome	11–20 vs 21–30 11–20 vs 31–40 11–20 vs 41–50	37.8 37.8 37.8	28.9 30.1 16.9	0.76 (0.48–1.21) 0.80 (0.51–1.25) 0.45 (0.26–0.76)	0.26 0.33 0.0038
Dengue	Brazil 2000–14^142^	Complications	6–10 vs 11–20 6–10 vs 21–35 11–20 vs 21–35	5.2 5.2 2.4	2.4 1.6 1.6	0.45 (0.44–0.46) 0.31 (0.30–0.32) 0.69 (0.68–0.71)	<0.0001 <0.0001 <0.0001

For children the age groups 5–14 and 10–14 years were used where available. For adults the age group 20–29 years was used where available. If there was no difference in outcome between children and adults aged 20–29, older age groups were examined. The largest dataset^*^ with available data was selected for each infection. Some datasets used different cut-points for age.

*The largest datasets were selected except for Spanish influenza, as the larger dataset shown in Supplementary Figure S9b combined “influenza, pneumonia and grippe”; and for Western equine encephalitis and E coli, for which datasets of large single outbreaks were selected rather than surveillance data (Supplementary Figures S29, S31).

## Code availability

There was no custom code for this analysis
