# Supplementary material for: Systematic analysis of infectious disease outcomes by age shows lowest severity in school-age children
Source: Sci Data. 2020 Oct 15;7:329. doi: 10.1038/s41597-020-00668-y (PMC7566589; doi:10.1038/s41597-020-00668-y)
Supplement: Supplementary file 1 — Supplementary information [file 41597_2020_668_MOESM1_ESM.pdf]

# Systematic analysis of infectious disease outcomes by age shows lowest severity in school-age children

## Appendix: datasets with information on severity of infectious disease by age

**Contents** **Page no.**


| | |
|---|---|
| Introduction | 2 |
| Polio (Fig S1) | 3 |
| Typhoid (Fig S2) | 5 |
| Tuberculosis (Fig S3) | 7 |
| Measles (Fig S4) | 9 |
| Smallpox (Fig S5) | 11 |
| Chickenpox (Fig S6) | 12 |
| Infectious mononucleosis (Fig S7) | 13 |
| HIV (Fig S8) | 14 |
| Influenza (Fig S9) | 15 |
| Pertussis (Fig S10) | 16 |
| Salmonella (Fig S11) | 17 |
| Yellow fever (Fig S12) | 19 |
| Typhus (Fig S13) | 20 |
| Scarlet fever (Fig S14) | 21 |
| Ebola (Fig S15) | 22 |
| Meningococcal meningitis (Fig S16) | 23 |
| Japanese encephalitis (Fig S17) | 24 |
| Cholera (Fig S18) | 25 |
| Lassa fever (Fig S19) | 28 |
| Brucellosis (Fig S20) | 29 |
| Hepatitis B (fig S21) | 30 |
| Plague (Fig S22) | 31 |
| Hepatitis A (Fig S23) | 33 |
| Severe acute respiratory syndrome (SARS) (Fig S24) | 34 |
| COIVD-19 (Fig S25) | 35 |
| Middle East respiratory syndrome (MERS-CoV) (Fig S26) | 37 |
| St Louis encephalitis (Fig S27) | 38 |
| Campylobacter (Fig S28) | 39 |
| Western Equine encephalitis (Fig S29) | 40 |
| Diphtheria (Fig S30) | 41 |
| Escherichia coli (Fig S31) | 42 |
| Dengue (Fig S32) | 43 |
| References | 44 |

## Introduction

Descriptions and figures for all identified datasets that fulfil the selection criteria described in the Methods are shown here. They are arranged by disease, following the order in figures 1-4 (i.e. by age at which severity of disease starts to rise). For each disease, a short description accompanies the figures. Each figure shows the point estimates of a measure of severity (e.g. case fatality rate, CFR) by age group. The legend gives the origins of the data (e.g. based on notifications, hospital data). Numbers of cases and binomial exact 95% confidence intervals are shown if available or if they could be calculated from the data given. Scales vary.

***Polio***

Most polio infections are asymptomatic but whether the case/infection ratio varies by age is not known.[5] Among diagnosed cases the CFR was lower in children aged 1-14 years than in older adolescents and adults in large studies in Europe and the US, though the pattern was less clear in smaller studies (Fig S1).[6-11]

**Fig S1 Polio** (a) England and Wales 1947-50, notified deaths / notified cases (23,143 cases);[12] (b) France 1952, notified deaths / notified cases (~1300 cases);[8] (c) Sweden 1935-44, CFR among 11,455 paralytic cases (estimates from graph);[11] (d) New York City 1916, notified deaths / notified cases;[9] (e) Small towns in New York State, US, 1915-24, notified deaths / notified cases (note: the numbers from which the fatality rates are derived are estimated from age-specific case and mortality rates and population data);[13] (f) Kentucky 1936, notified deaths / notified cases;[10] (g) Manitoba, Canada, 1941, CFR;[7] (h) Scotland, UK, 1947, CFR.[6]

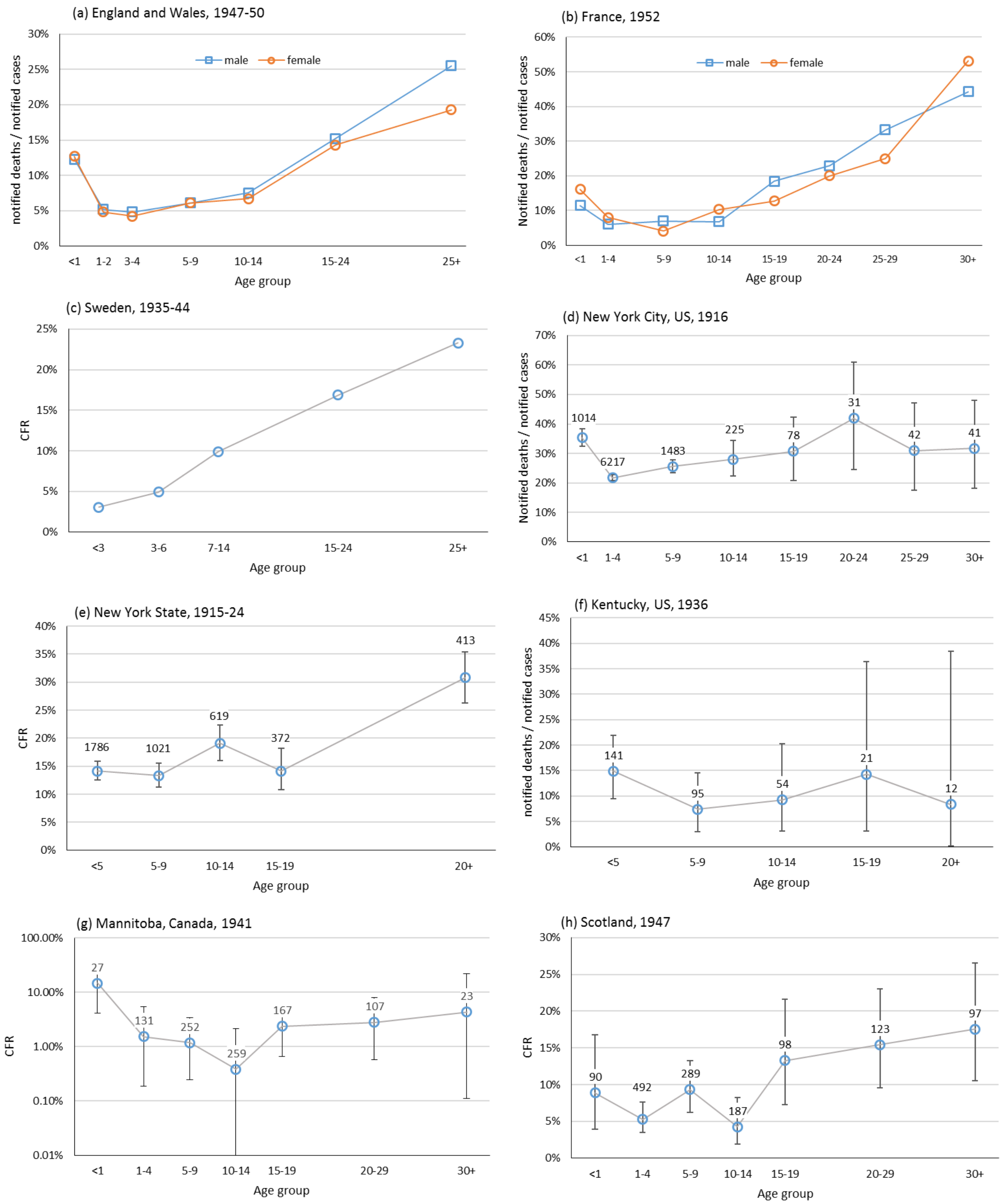

***Typhoid***

Notification data on >16,000 patients with typhoid in New York State, US, 1915-24, show a 'J-shaped' pattern of the CFR with age, lowest at 5-9 years and rising steadily thereafter (Fig S2a).[13] Hospital data on >12000 patients from Glasgow 1866-1914 show a similar pattern (Fig S2b).[14] Smaller datasets from the London Fever Hospital in 1848-57,[15] and from individual epidemics in the UK[16-19] and Australia[20] all show an increase in CFR with age but with less clear patterns (Fig S2c-h). The number of cases in children <5 years is generally lower than expected from mortality data, suggesting they are under-represented in these data.

**Fig S2 Typhoid** (a) Small towns in New York State, US, 1915-24, notified deaths / notified cases;[13] (b) Glasgow fever hospitals, UK, 1866-1914, CFR;[14] (c) London Fever Hospital, UK, 1848-57, CFR;[15] (d) Stockport, UK, 1893, notified deaths / notified cases;[19] (e) Warrington, UK, 1899, CFR;[18] (f) Plumstead, Kent, UK, 1896, notified deaths / notified cases;[16] (g) Croydon, UK, 1937, CFR;[17] (h) Moorabbin, Australia, 1943, CFR.[20]

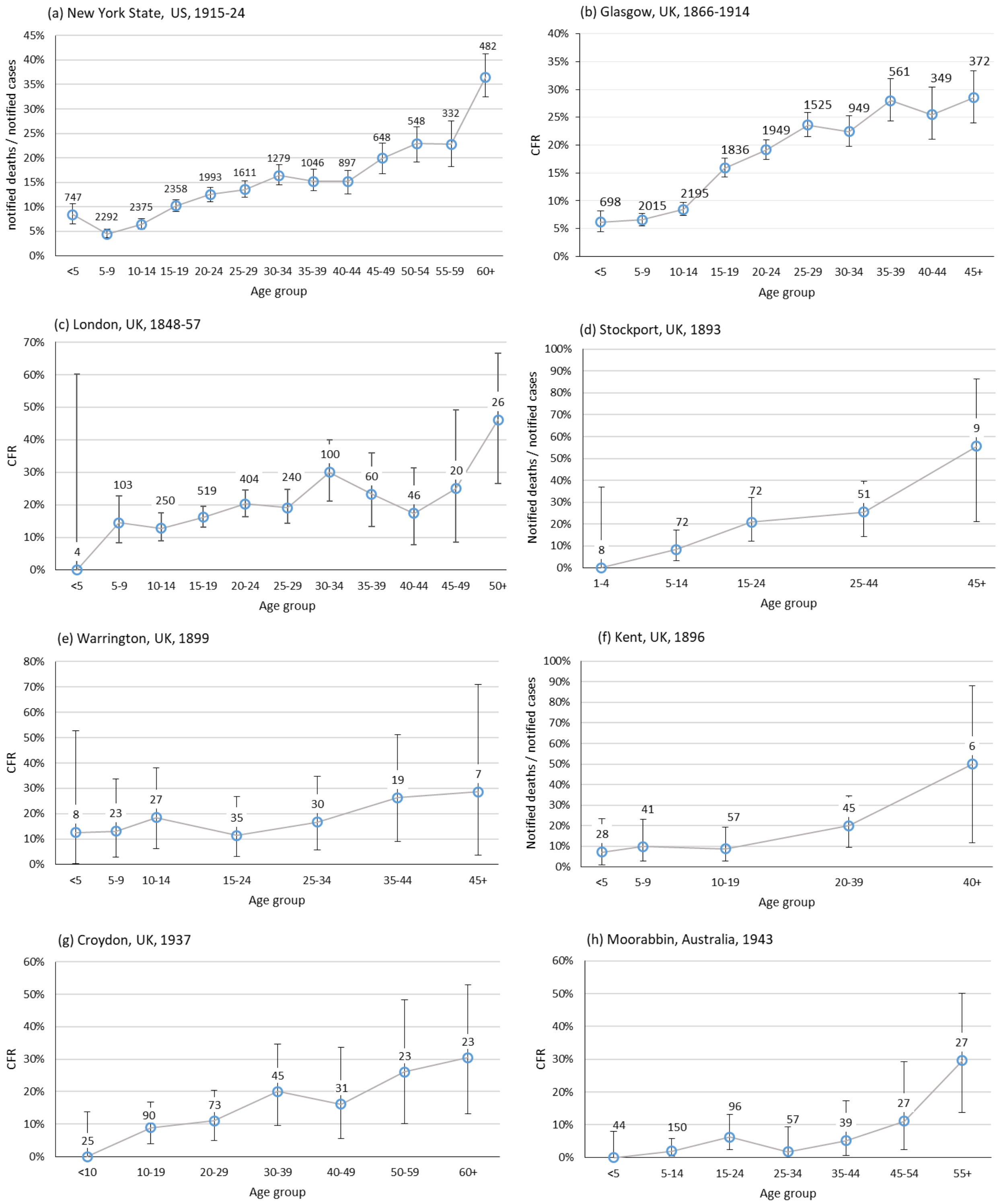

### *Tuberculosis*

In Denmark, of nearly 40,000 pulmonary tuberculosis (TB) cases notified in 1925-34, the proportion who had died by 31st December 1934 was lowest at age 5-9 years rising steadily thereafter (Figs 1c, S3a).[21] In Glasgow, UK, 3425 patients with pulmonary tuberculosis in 1910 were followed for the next 4 years.[22] The CFR was much lower in the 5-9 and 10-14 year age groups than in any other group, and the pattern was similar when those who were lost to follow-up were excluded (Fig S3c). Notification data from four UK cities in 1910-13,[22] and from Wales in 1936-8[23] show a 'J-shaped' pattern of age-specific CFRs, with the nadir at 5-14 years (Fig S3c-f). The CFR based on notification rates can be difficult to interpret as screening for tuberculosis may be carried out in certain age groups (e.g. school children),[22] and with a chronic disease with decreasing incidence, deaths in a particular year may outnumber new cases.[23] However in Denmark, comparing the number of notified deaths to the number of notified cases gave a very similar pattern by age to the CFR measured directly for the notified cases (Fig S3a,b).[21] The age-specific risk of tuberculosis following exposure shows a similar dip in school-age children.[24] Among 12,594 household contacts followed in the US, the risk of developing TB within one year was much lower at ages 5-9 and 10-14 years than in young adults (Fig S3g).[25] And among more than 82,000 Puerto Rican children in the US who had skin-test evidence of infection and were followed for nearly 19 years, disease incidence dropped between ages 8 and 12 years.[26]

**Fig S3 TB** (a) Pulmonary TB, Denmark, 1925-34, percent of notified cases dying by 31st December 1934;[21] (b) Pulmonary TB, Denmark, 1925-34, notified deaths / notified cases;[21] (c) Pulmonary TB, Glasgow, UK, 1910-14, risk of dying from TB over 4 years;[22] (d) Pulmonary TB, Glasgow, UK, 1910-12 (~7000 cases) and Birmingham, UK, 1912-13 (~9000 cases) , notified deaths / notified cases;[22] (e) Pulmonary TB, Portsmouth (1267 cases) and Sheffield (980 cases), UK, 1912, notified deaths / notified cases;[22] (f) Pulmonary TB, Wales, 1936-38 (9339 cases), notified deaths / notified cases;[23] (g) Risk of TB (pulmonary or extrapulmonary) among household contacts within 1 year of diagnosis of TB in index case, US 1957-60.[25]

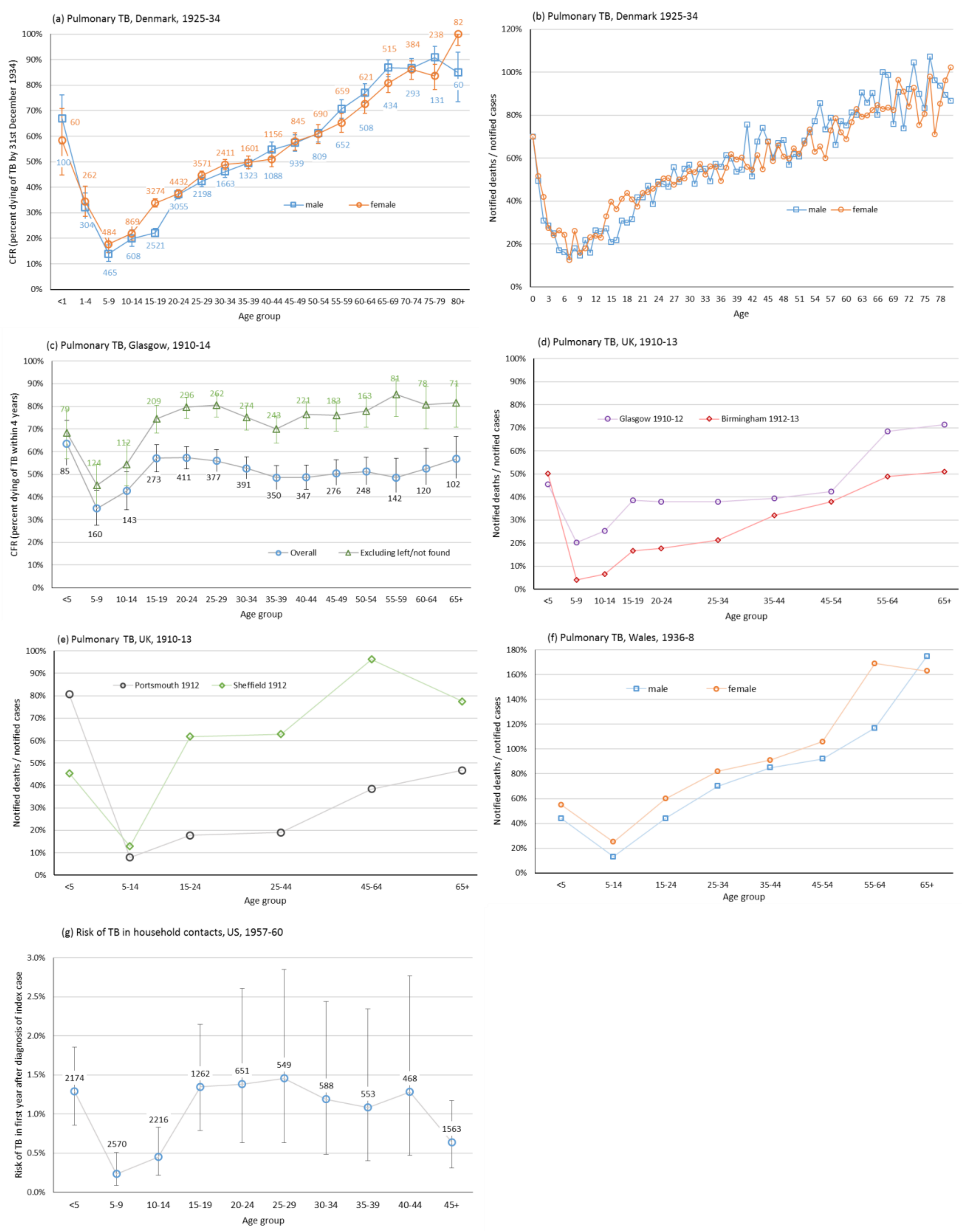
(a) Pulmonary TB, Denmark, 1925-34
CFR (percent dying of TB by 31st December 1934)
Age group
male
female
(b) Pulmonary TB, Denmark 1925-34
Notified deaths / notified cases
Age
male
female
(c) Pulmonary TB, Glasgow, 1910-14
CFR (percent dying of TB within 4 years)
Age group
Overall
Excluding left/not found
(d) Pulmonary TB, UK, 1910-13
Notified deaths / notified cases
Age group
Glasgow 1910-12
Birmingham 1912-13
(e) Pulmonary TB, UK, 1910-13
Notified deaths / notified cases
Age group
Portsmouth 1912
Sheffield 1912
(f) Pulmonary TB, Wales, 1936-8
Notified deaths / notified cases
Age group
male
female
(g) Risk of TB in household contacts, US, 1957-60
Risk of TB in first year after diagnosis of index case
Age group

*Measles*

Before vaccination was introduced, measles infection in childhood was almost universal, so reports including adults are rare. Panum described an outbreak in the Faroe Islands in 1846, where measles had been absent for 65 years.[27] Compared to previous years, all-cause mortality was very high in infants, unchanged at age 1-19 years, and increased with age between 20-60 years. Estimated CFRs (given in the appendix to his report) were lowest at 10-19 years, but implicitly assume a constant attack rate by age so confidence intervals have not been calculated (Fig S4a). Surveillance data are available from Aberdeen, UK in 1883-1902, Kansas, US in 1914-15 and New York State, US, in 1915-24.[9,13,28] These show the lowest CFR in children over 5 years, with an increase from young adulthood (Fig S4b-d). In England and Wales in 1971-88 the case fatality rate based on more than 2 million notifications was lowest at age 5-9 years; vaccination status is not given (Fig S4e).[29] Data from unvaccinated individuals in Romania 2016-18 appear to show a similar pattern although confidence intervals are wide (Fig S4f),[30,31] and only one of those dying over the age of 2 years had no underlying illness. Data from partially vaccinated subjects in Bulgaria in 2009-11 show a later rise with age (Fig S4g).[32] In The Netherlands in 2013-14 the proportion hospitalised was lowest at ages 4-17 years (Fig S4h).[33]

**Fig S4 Measles**. (a) Faroe Islands 1846, estimated CFR (6100 cases);[27] (b) Aberdeen 1883-1902, notified deaths / notified cases;[28] (c) Kansas, US, 1914-15, notified deaths / notified cases[9] (d) Small towns in New York State, US, 1915-24, notified deaths / notified cases;[13] (e) England and Wales 1970-88, notified deaths / notified cases;[29] (f) Romania 2016-19, notified deaths / notified cases in unvaccinated individuals;[30,31] (g) Bulgaria, 2009-11, CFR, 41% unvaccinated, 49% had had one dose of vaccine (of whom 11% were vaccinated within 14 days of onset of measles), 10% had had 2 or more doses;[32] (h) The Netherlands, 2013-14, percent hospitalised (94% unvaccinated).[33]

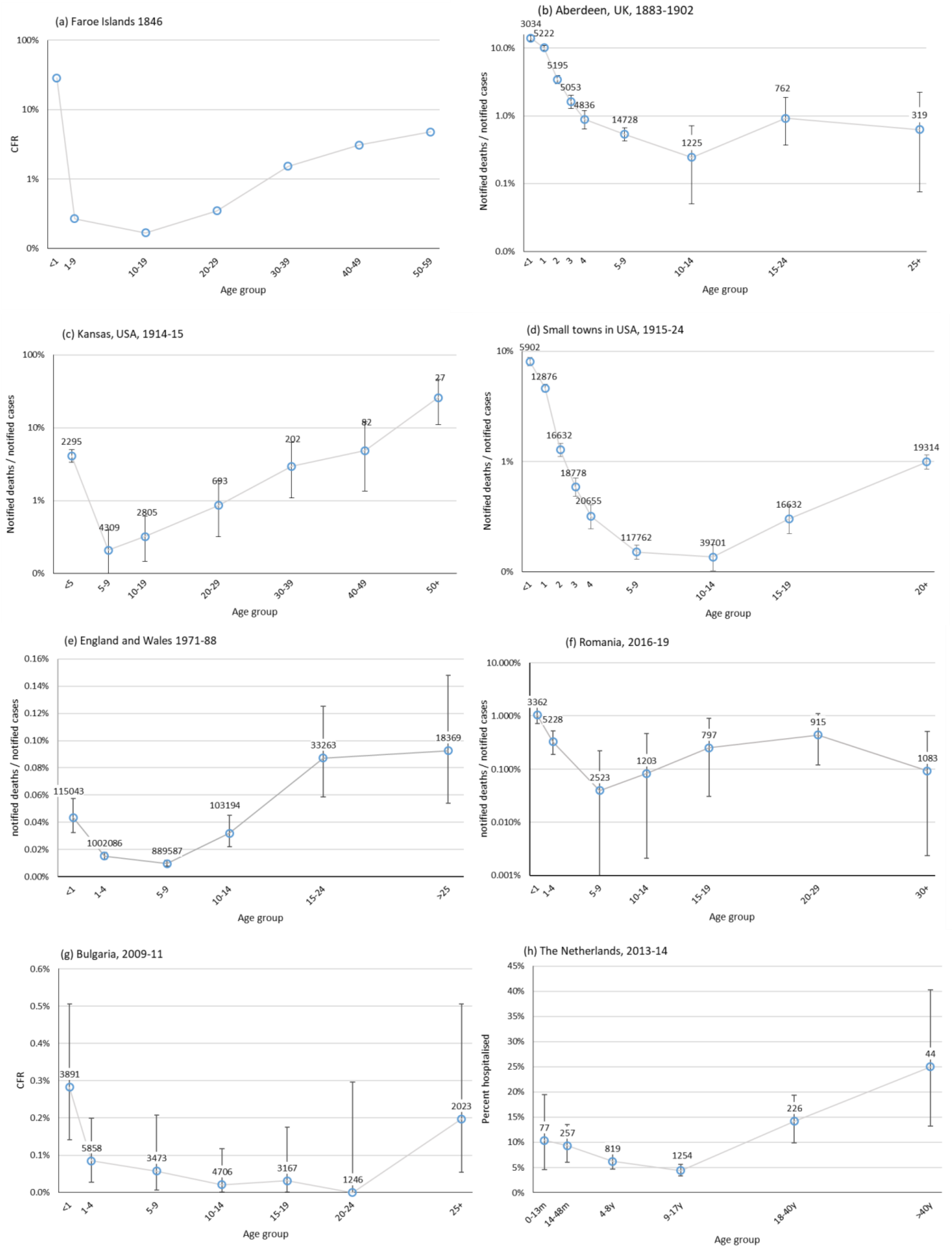
(a) Faroe Islands 1846
CFR
100%
10%
1%
0%
<1
1-9
10-19
20-29
30-39
40-49
50-59
Age group
(b) Aberdeen, UK, 1883-1902
Notified deaths / notified cases
10.0%
1.0%
0.1%
0.0%
3034
5222
5195
5053
4836
14728
1225
762
319
<1
1
2
3
4
5-9
10-14
15-24
25+
Age group
(c) Kansas, USA, 1914-15
Notified deaths / notified cases
100%
10%
1%
0%
2295
4309
2805
693
202
82
27
<5
5-9
10-19
20-29
30-39
40-49
50+
Age group
(d) Small towns in USA, 1915-24
Notified deaths / notified cases
10%
1%
0%
5902
12876
16632
18778
20655
117762
39701
16632
19314
<1
1
2
3
4
5-9
10-14
15-19
20+
Age group
(e) England and Wales 1971-88
notified deaths / notified cases
0.16%
0.14%
0.12%
0.10%
0.08%
0.06%
0.04%
0.02%
0.00%
115043
1002086
889587
103194
33263
18369
<1
1-4
5-9
10-14
15-24
>25
Age group
(f) Romania, 2016-19
notified deaths / notified cases
10.000%
1.000%
0.100%
0.010%
0.001%
3362
5228
2523
1203
797
915
1083
<1
1-4
5-9
10-14
15-19
20-29
30+
Age group
(g) Bulgaria, 2009-11
CFR
0.6%
0.5%
0.4%
0.3%
0.2%
0.1%
0.0%
3891
5858
3473
4706
3167
1246
2023
<1
1-4
5-9
10-14
15-19
20-24
25+
Age group
(h) The Netherlands, 2013-14
Percent hospitalised
45%
40%
35%
30%
25%
20%
15%
10%
5%
0%
77
257
819
1254
226
44
0-13m
14-48m
4-8y
9-17y
18-40y
>40y
Age group

## *Smallpox*

The CFR amongst unvaccinated individuals is available from several smallpox outbreaks in the UK in the 19th century (Fig S5). In Sheffield in 1887-8 (Fig S5b), house-to-house enumeration ensured inclusion of mild non-hospitalised cases and identification of "re-attacked" cases (presumably those reported to have had smallpox previously, who might have milder disease).[34] Other studies were restricted to hospitalised cases or estimated from notifications of cases and deaths (Fig S5).[35-39] All show a 'J-shaped' pattern of CFR with age with the lowest rates in those aged around 5-14 years.

**Fig S5 Smallpox** Among unvaccinated individuals (a) London Smallpox Hospital,UK, 1836-51, CFR;[35] (b) Sheffield, UK, 1887-8, CFR among cases in enumerated houses;[34] (c) Birmingham UK 1893-5, notified deaths/notified cases;[39] (d) Middlesborough, UK, 1898, CFR;[37] (e) Leicester, UK, 1903, CFR among hospitalised patients;[36] (f) Westhulme Hospital Oldham, UK, 1892-3, CFR.[38]

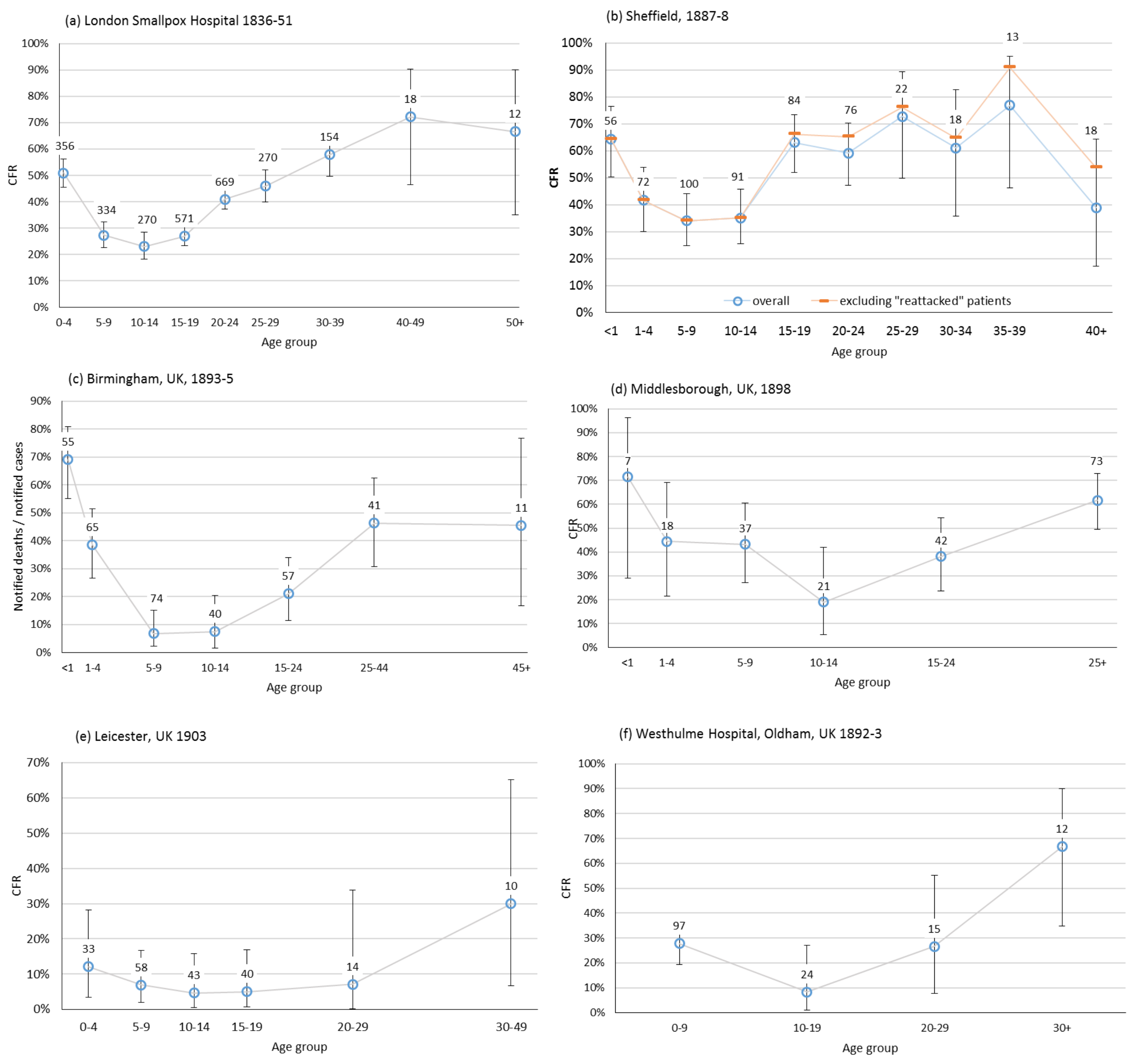

***Chickenpox***

The proportion of chickenpox cases leading to hospitalisation in France in 1997-9, estimated from national databases and surveillance, shows a 'J-shaped' pattern with age, with much higher rates at 15-24 than at 5-14 years (Fig S6a).[40]A similar pattern was observed for the CFR but with wide confidence intervals. Estimates of the proportion of cases hospitalised based on sample surveys and surveillance in the US, Canada, and England and Wales were a similar shape, with the lowest proportion hospitalised in age groups 5-9, 5-11 and 12-18 years respectively. (Fig S6b-d).[41,42]

**Fig S6 Chickenpox**. Estimated hospitalisation per 100,000 cases (unvaccinated) (a) France 1997-9, based on national databases and surveillance (3306 hospitalisations);[40] (b) US 1979-82, based on sample surveys and average 3837 hospitalisations per year;[41] (c) Manitoba, Canada, 1979-97, based on physician billing claims and hospital records;[42] (d) England and Wales, based on sentinel surveillance in general practices and national surveillance of hospitalisations.[42]

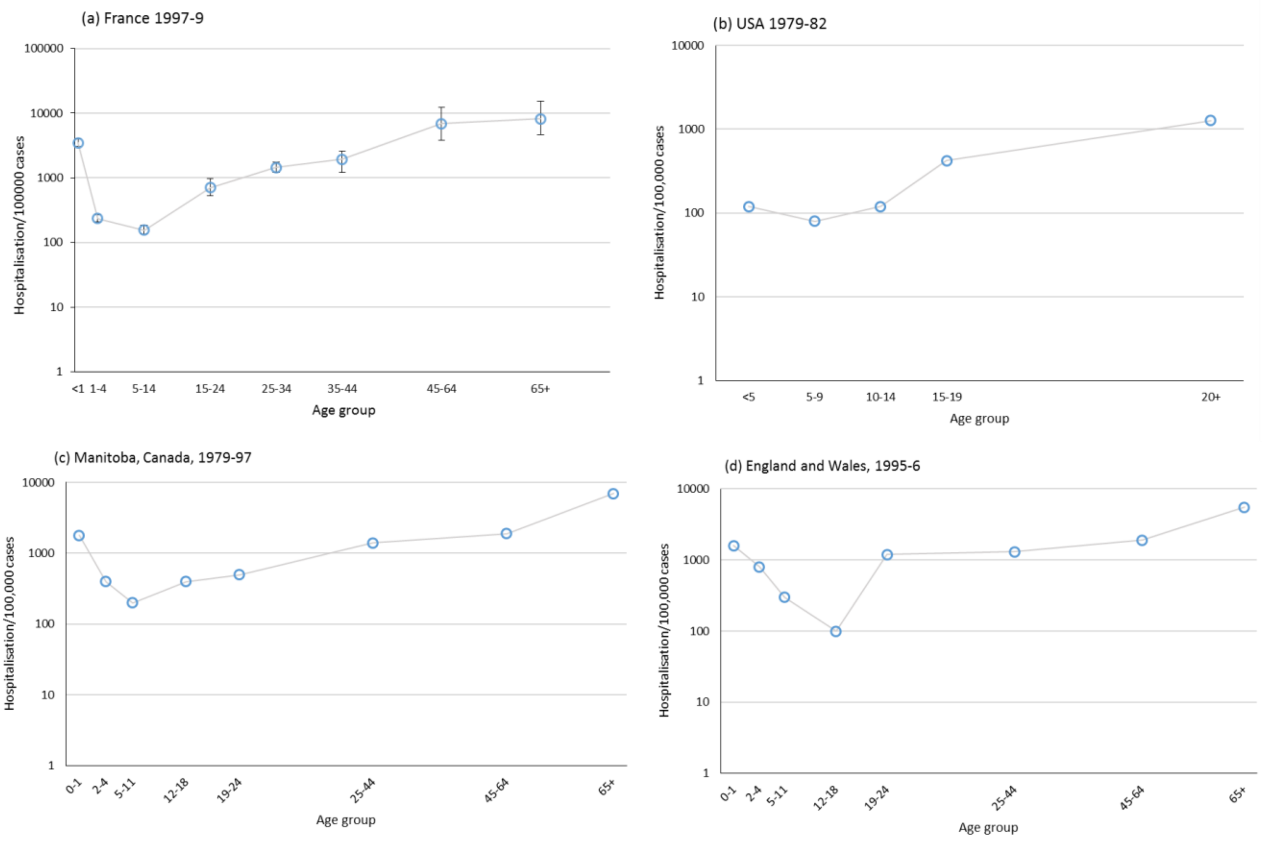

### *Infectious mononucleosis*

In Rochester, Minnesota, US, medical records from 1950-69 were reviewed to extract data on patients with clinical, haematological or serological evidence of infectious mononucleosis.[43] Among 776 confirmed cases, hospitalisation rates were lowest at age 5-14 years, much lower than in those aged 15-18 years or older (Figs 1f, S7).

**Fig S7 Infectious mononucleosis** (a) Rochester Minnesota, US, 1950-69. Percent hospitalised (the age groups were based on school and college ages).[43]

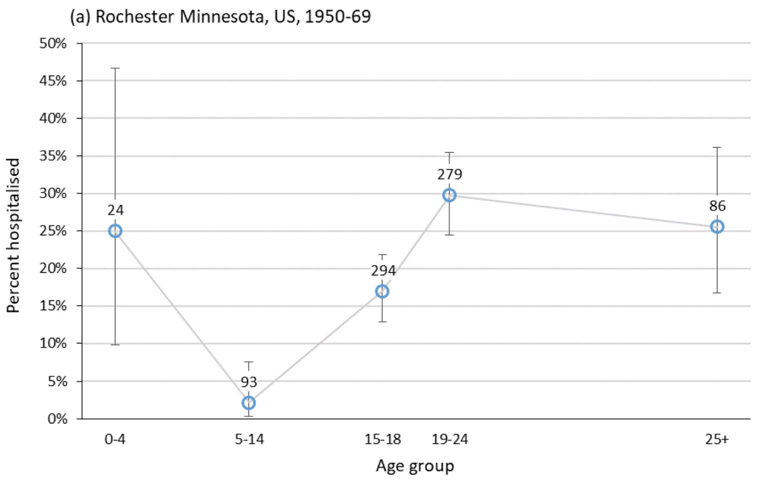

### *HIV*

In a pooled analysis of 13,030 individuals with reliable estimates of date of HIV infection (seroconversion), excluding those who were vertically infected, the time to AIDS and to death decreased for each 10-year increase in age.[44] The median survival for those aged <5 years or 5-14 years at seroconversion was not reached during follow-up, but was more than 13 years. This compares to a median survival of 12·5 years (95% CI 12·1–12·9) for those aged 15-24, 10·9 years (10·6–11·3) for those aged 25-34, 9·1 years (8·7–9·5) for 35-44, 7·9 years (7·4–8·5) for 45-54, 6·1 years (5·5–7·0) for 55-64 and 4·0 years (3·4–4·6) for those aged 65 and over (Fig 1h, S8). Most of the younger individuals were haemophiliacs infected through blood products. Haemophiliacs had slightly better survival than the other groups, but in a separate analysis restricted to haemophiliacs the same age pattern was seen.[45] There were no other differences in mortality by risk group or route of infection.[44]

**Fig S8 HIV**. Europe, North America, Australia, 1983-1996. Percent dying within 10 years of seroconversion. Approximate estimates based on figure of survival analysis. (Mother-to-child infections excluded).[44]

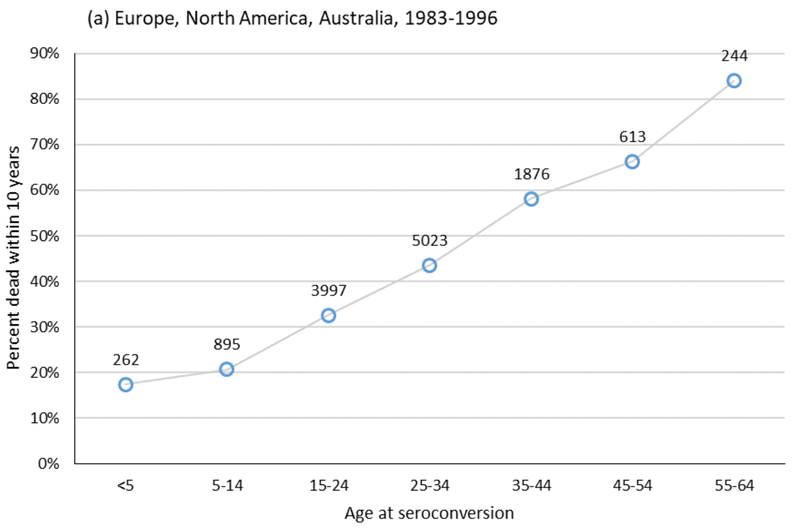

### *Influenza*

The 1918-19 "Spanish" influenza epidemic had a relatively low mortality rate in children but high rate in young adults[4,46] Two large studies based on household surveys in the US showed that the CFR followed a similar age pattern to the mortality rates, despite a lower incidence in children (Fig 2a, S9a,b).[47,48] In both studies the lowest CFR was in children aged 5-14 years. The CFR rose quickly in young adults (higher in men than women) and then fell again in middle-age before rising in the over 60s. This dip in middle-age has been attributed to protective immunity from exposure to H1-like viruses prior to 1889, and was not seen in mortality rates from Spanish influenza in regions with different previous epidemics.[4,49-52] In other influenza epidemics the case fatality rates are much lower and deaths in recent outbreaks are rare in those without underlying medical conditions.[53] In a US household survey of the 1928-29 influenza epidemic, the case fatality rate was lowest at age 5-9 years but remained low until after age 45 years (Fig S9c).[48]

**Fig S9 Influenza** (a) Maryland, US, 1918-19, CFR from household surveys;[47] (b) Influenza, pneumonia and grippe in 11 localities in the US in 1918-19, CFR from household surveys;[48] (c) Influenza, pneumonia and grippe in 14 localities in the US in 1928-29, CFR from household surveys.[48]

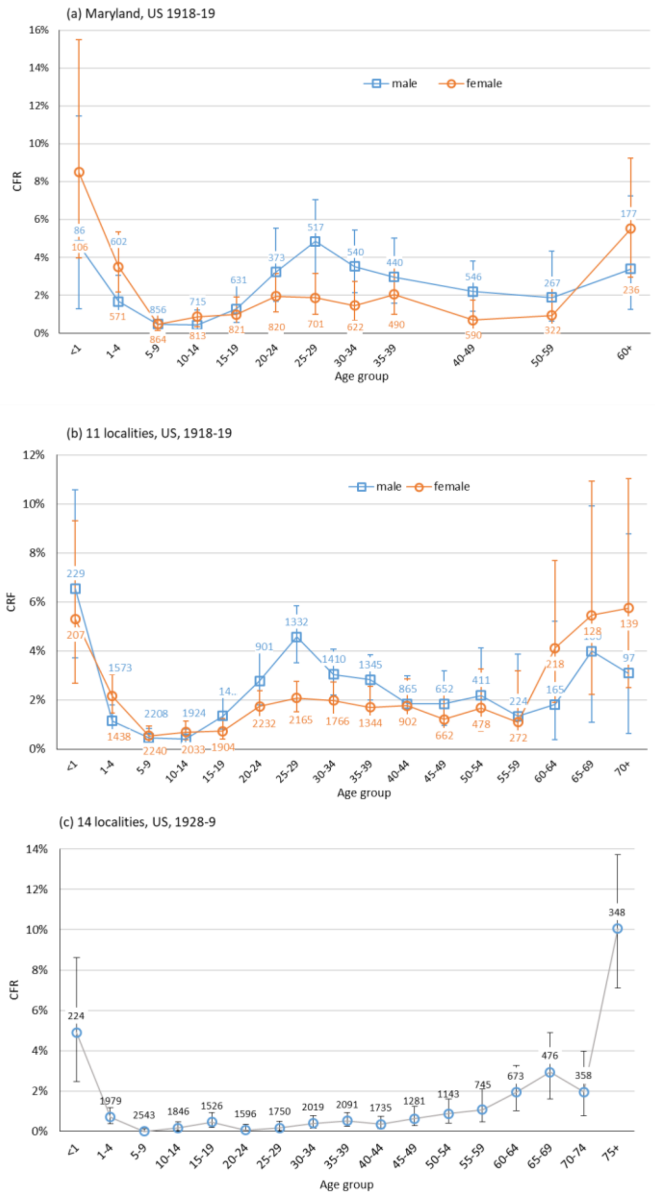

### *Pertussis*

Before vaccination, pertussis was rare in adults. Of 17,585 cases notified in Aberdeen in 1891-1900 only 89 were 15 years or older.[54] The CFR dropped quickly with age, with only one death over the age of 8 years (Fig S10a). In small towns in New York State, US, in 1915-24, the proportion dying was lower at 5-14 years than in those aged 15-19 or over (Fig 2b, S10b).[13]

**Fig S10 Pertussis** (a) Aberdeen, UK, 1891-1900, notified deaths / notified cases;[54] (b) Small towns in New York State, US, 1915-24, notified deaths / notified cases (note: the numbers from are estimated from the rates and population data).[13]

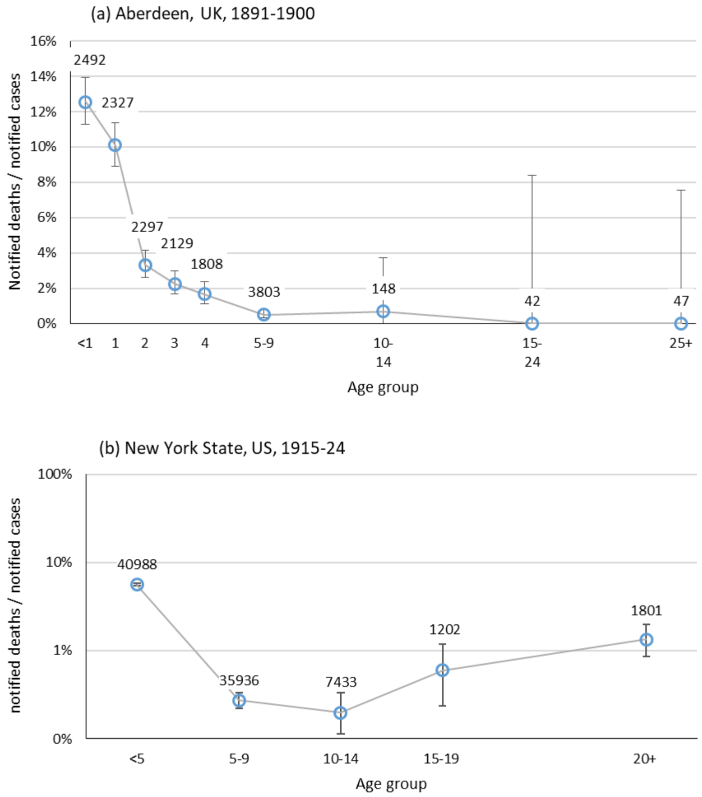

***Salmonella***

Most reports do not differentiate the different non-typhoid salmonellas. Among hospitalised patients in Spain the CFR rose with age from age 15 years,[55] and in Taiwan the proportion with septicaemia increased similarly with age.[56]. The proportion hospitalised, estimated by comparing notification data and hospital statistics in Canada[57] and Australia,[58] and directly in Denmark,[59] rose more slowly with age. In Belgium the lowest proportion with septicaemia was in young children, but confidence intervals were wide.[60] Investigation of a large point-source outbreak of *Salmonella Newport* in Sweden in 1960 identified 626 individuals with *S Newport* in their stools.[61] The proportion symptomatic was lowest at 7-15 years, half that in those aged 16-24 years (Fig 2c, S11). School-based testing may have contributed to the number of asymptomatic individuals identified at younger ages.

**Fig S11 Salmonella** (a) Spain, 1997-2006, CFR among hospital patients (numbers estimated from graph, and from population data from UN Population Division;[55] (b) Taiwan, 2006-8, percent of hospitalised patients with septicaemia;[56] (c) Canada, 2001-4, percent of cases hospitalised (estimated from notification data and hospital morbidity database);[57] (d) Australian Capital Territory, 2003-12, percent hospitalised, (estimated from graph, 1469 cases, 205 hospitalised);[58] (e) Funen County, Denmark, 1991-9, percent hospitalised (estimated from graph, 3328 cases, 952 hospitalised);[59] (f,g) Leuven, Belgium,1973-92, proportion septicaemic (those with isolates from blood / isolates from blood or faeces), separately for *S typhimurium* and *S enteritidis*;[60] (h) *S Newport*, Uppsala, Sweden, 1960, proportion symptomatic.[61]

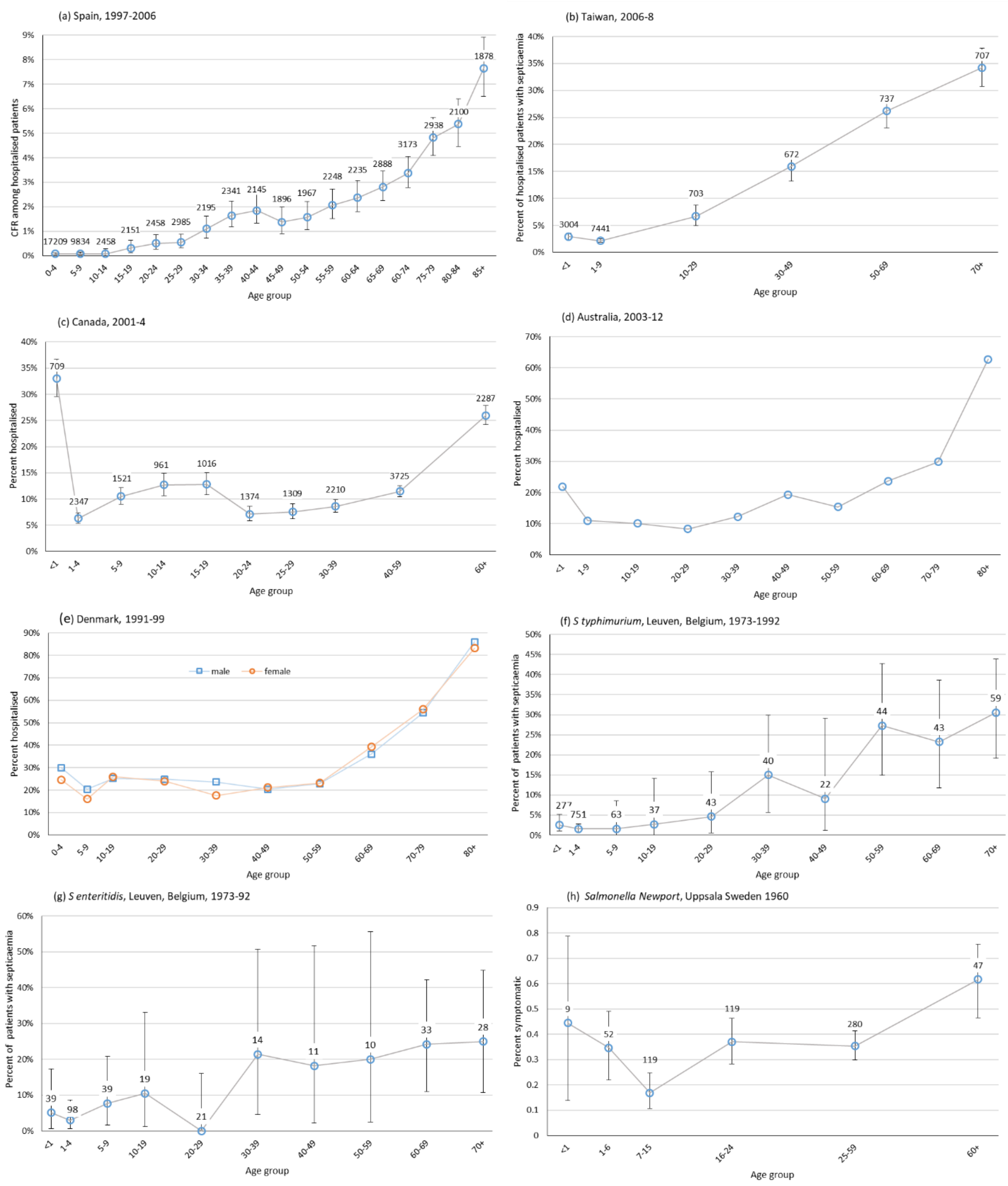

***Yellow Fever***

In Yellow fever outbreaks in 1878 in the US, the CFR among white patients was lowest at age 10-19 years in New Orleans (Fig 2d, S12a)[62] and at 10-14 years in Kentucky (Fig S12b).[62] There were relatively few deaths among non-white patients.

**Fig S12 Yellow fever** (a) New Orleans, US, 1878, CFR among white patients seen by 4 private practitioners (only 2 of 66 non-white patients died);[63] (b) Kentucky, US, 1878, CFR among white patients.[62]

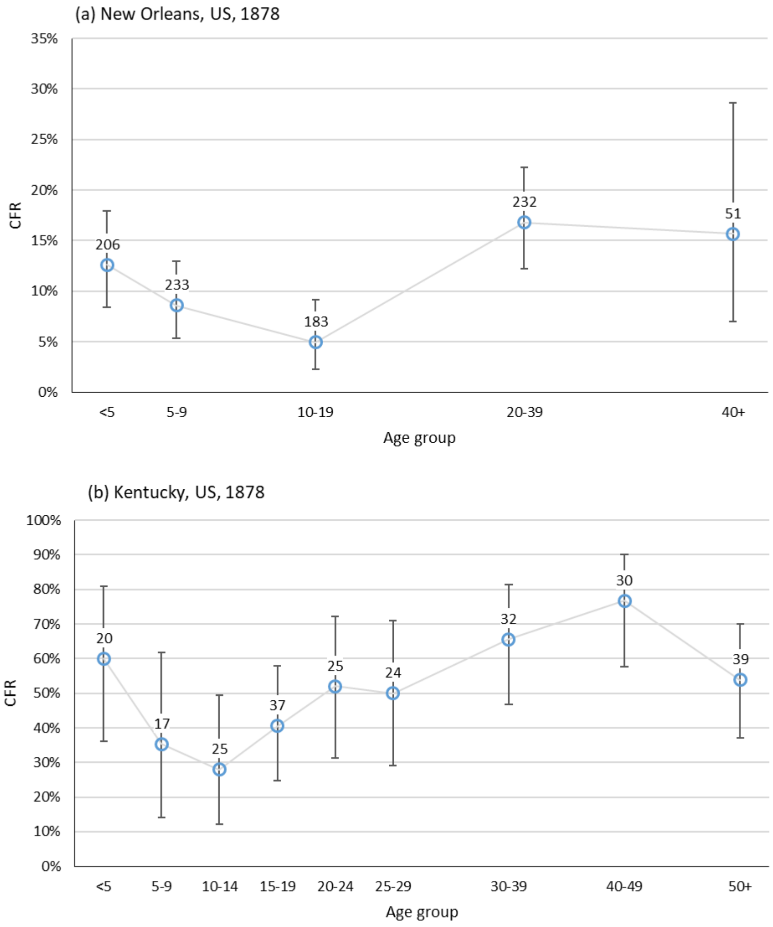

### *Typhus*

Typhus was the major cause of fever in patients admitted to the London Fever Hospital, London, UK, in 1848-57.[15] The CFR showed a J-shape pattern with age, lowest at 10-19 years (Fig 2e, S13).

**Fig S13 Typhus** (a) London Fever Hospital 1848-57, CFR[15]

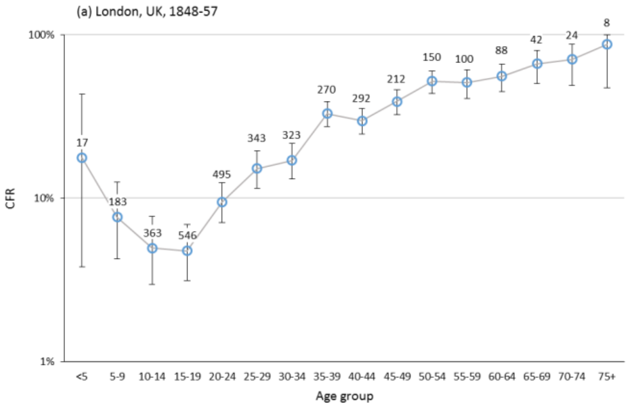

### *Scarlet fever*

For scarlet fever, adult cases were uncommon.[9] In Pennsylvania, US, 1907-12, and London, UK, 1895-1914, the age-specific CFR was lowest at 10-19 years, with clearly higher rates after age 20 years (Fig 2f, S14a,c).[9,64] Among hospitalised patients in London, UK, 1874-91, the CFR rose more slowly with age (Fig S14b).[65]

**Fig S14 Scarlet fever** (a) Pennsylvania, US, 1907-12, notified deaths / notified cases;[9] (b) London, UK, 1871-94, CFR in 81,350 hospitalised cases (numbers by age group not given);[65] (c) Eastern Hospital, London, UK, 1895-1914, CFR.[64]

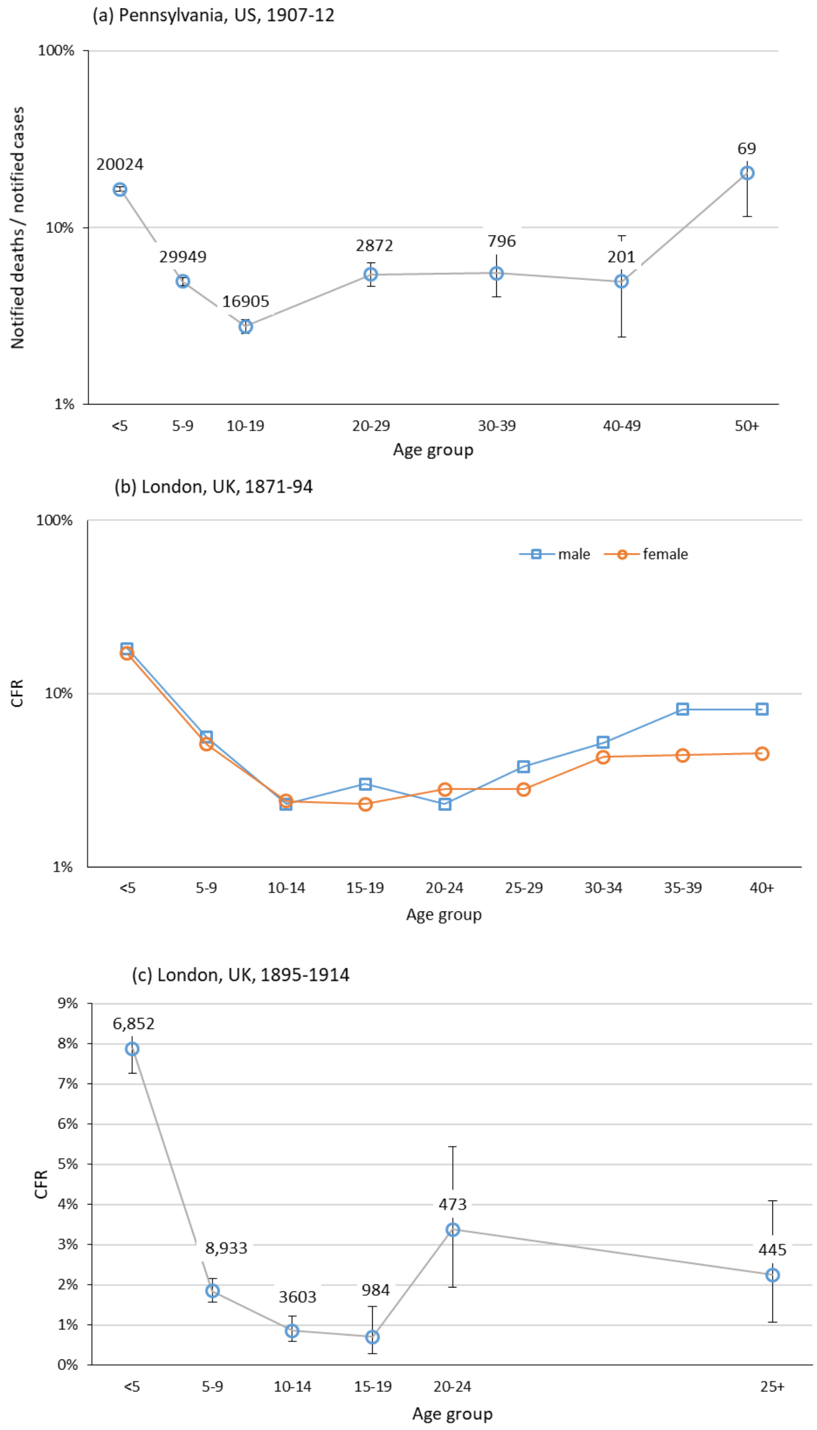

### *Ebola*

In the West African Ebola epidemic large numbers of people were affected who (probably) had no prior exposure to Ebola virus. The age-specific CFRs published by the World Health Organisation are 'J-shaped', with a nadir at 10-19 years, similar for males and females (Figs 2g, S15a).[66] Outcome data were missing for half of the cases and subclinical cases would not have been included. A study of Ebola-affected households in Sierra Leone, including serological testing, found a similar pattern (Fig S15b).[67,68] In this household study, the proportion of those exposed who developed Ebola Virus Disease showed a similar 'J-shaped' pattern with age (Fig S15c). The extent of exposure to patients with Ebola Virus Disease and their bodily fluids (a proxy of infectious dose) was strongly associated with the attack rate, but did not influence the case fatality rate or fully explain the age-specific attack rate pattern.[67,69]

**Fig S15 Ebola**. (a) Guinea, Liberia and Sierra Leone 2013-15. CFR among confirmed and probable cases with known outcome (9,393 of 20,035 cases with data on sex);[66] (b,c) Sierra Leone 2014-15, CFR (b) and attack rate (c) among household members in 94 Ebola-affected households.[67]

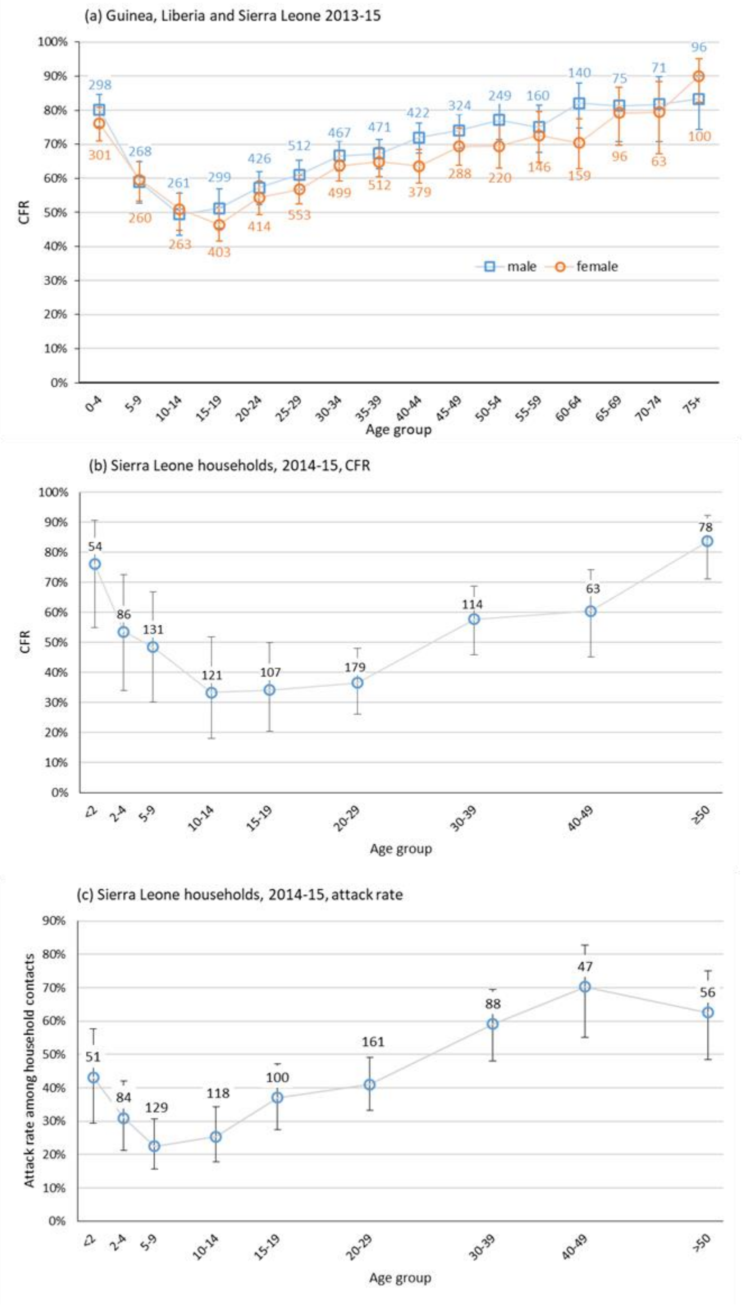

### *Meningococcal meningitis*

In a large outbreak of meningitis in Cyprus in 1908-9, the age-specific CFR pattern was 'J-shaped', with the nadir at 10-19 years (Fig 2h, S16a).[70] In a smaller outbreak in California in 1929, and among hospitalised patients in New York in 1901-6, the CFR was also relatively low in younger children (Fig S16bc).[71,72]

**Fig S16 Meningococcal meningitis** (a) Cyprus 1908-9, CFR;[70] (b) California 1929, notified deaths / notified cases;[71] (c) Mount Sinai Hospital, New York, US, 1901-6, CFR.[72]

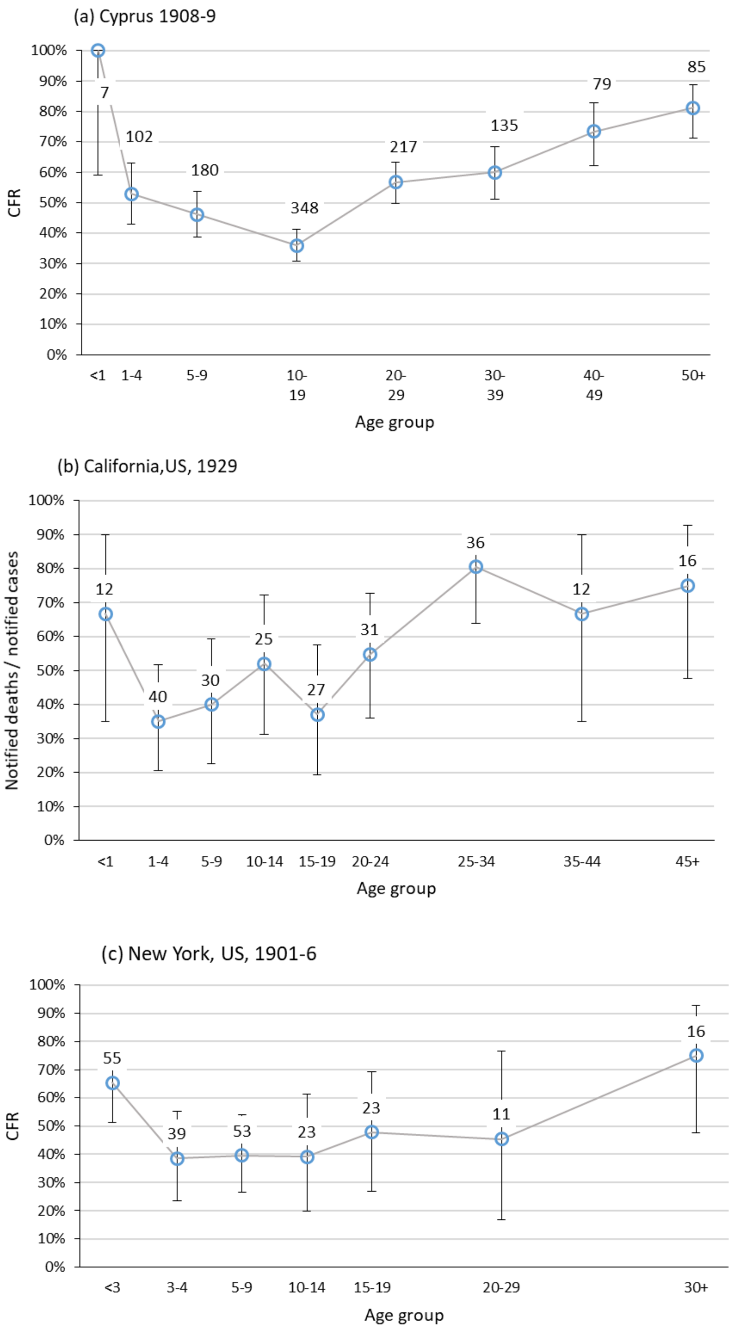

### *Japanese encephalitis*

Surveillance data from Korea, Japan and Nepal show an increase in CFR by age after age 25-30 years, and no evidence of a higher CFR in children <5 years than in older children (Fig 3b, S18a-c).[73,74]

**Fig S17 Japanese encephalitis** (a) Korea, 1955-66, notified deaths / notified cases;[73] (b) Japan, 1965, CFR;[73] (c) Nepal, 2007-2015, notified deaths / notified cases.[74]

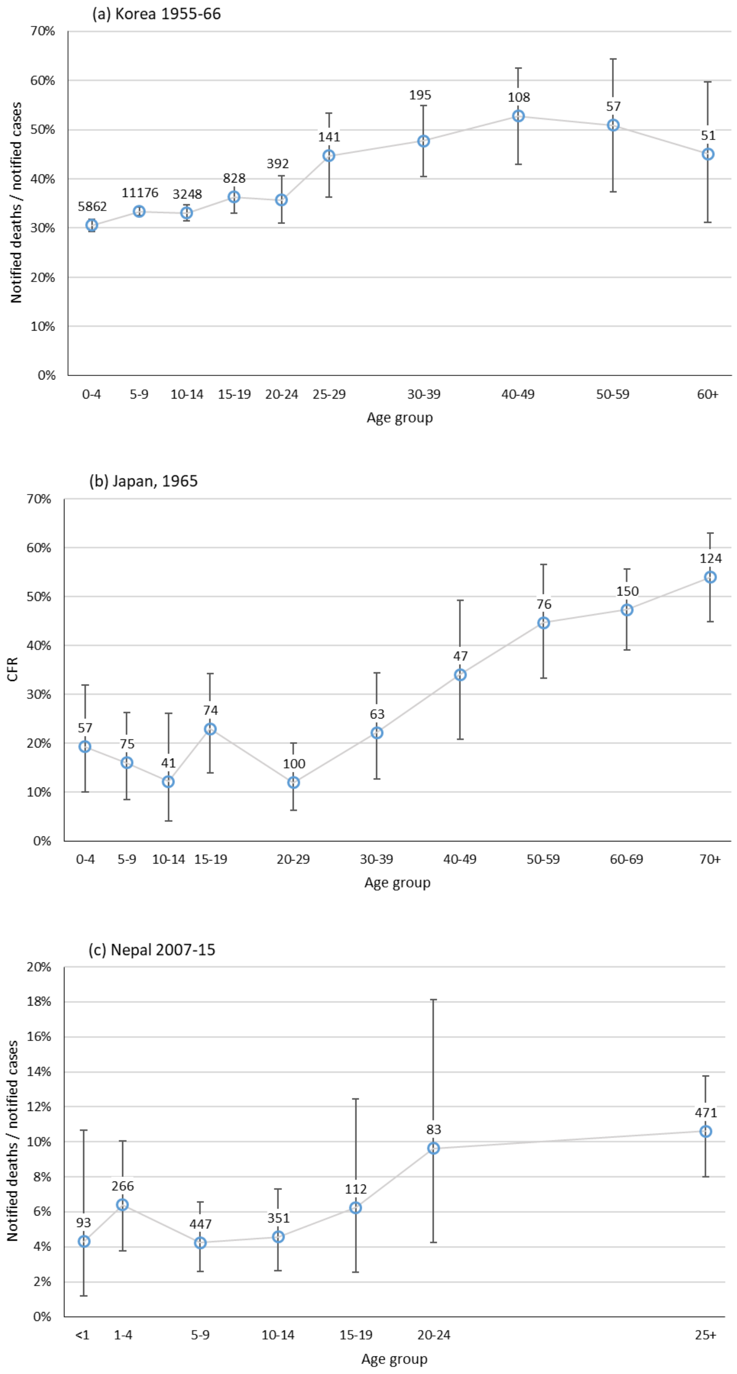

### *Cholera*

In a large outbreak of cholera in Hamburg in 1892 the CFR was lowest at age 15-24 years (Fig S19a).[75] In Munich, Germany, in 1873-4 and Glasgow, UK, in 1848-9 the CFR was lowest at 10-19 years (Fig 3c, S19b,c).[76] Other estimates of the CFR from European outbreaks are based on much smaller numbers, and found the lowest CFR at 5-14, 15-24, 10-19 or 20-29 years in different studies (Fig S19d-h).[77-81] In Jamaica in 1851[82] and Shanghai, China in 1907,[83] where the CFR was lower, there was a less clear pattern with age (Fig S22i,j), and in Calcutta, India, in 1905 the CFR rose with age from age 20 years (Fig S19k).[84]

In more recent outbreaks, rehydration therapy has dramatically increased survival. Analysis of data from a study in Haiti, including cases not seen in medical facilities, shows a CFR of 11%, lowest at 10-14 years, and rising only slowly with age (Fig S19l),[85] with a similar pattern when adjusted for care-seeking, district and sex (not shown). Among patients with severe cholera treated with intravenous fluids in the Philippines, the lowest CFR was at 20-29 years (Fig S19m).[86] In Katsina, Nigeria, 1961-2, among hospitalised patients with cholera Ogawa, there was little variation in CFR by age (Fig S19n).[87]

In large water-borne outbreaks of cholera, exposure of all age-groups is expected (though breastfed infants may be less exposed) so mortality rates can be informative. The mortality rates from cholera in England in 1849, based on 72,110 deaths,[88] and Paris, France, in 1832, based on 18,402 deaths, were lowest at age 5-14 years.[89] In central India in 1875-6, the mortality rates (excluding infants) were lowest at age 12-19 years.[90](Fig S19o-q)

**Fig S18 Cholera** (a) Hamburg, Germany, 1892, notified deaths / notified cases;[75] (b) Munich, Germany, 1873-4, notified deaths / notified cases;[76] (c) Glasgow, UK, 1848-9, notified deaths / notified cases;[76] (d) Clonmel, Ireland, 1849, patients at Clonmel District Cholera Hospital, CFR;[79] (e) Dublin, Ireland, Mater Misericordiae Hospital, 1866, CFR;[80] (f) Prague (now in Czech Republic), 1850, CFR; [81] (g) London, UK, 1854, inpatients at Middlesex Hospital, CFR;[77] (h) Oxford, UK, 1854, CFR;[78] (i) Jamaica, 1851, cases treated by one physician, CFR;[82] (j) Isolation hospital, Shanghai, 1907, CFR;[83] (k) Calcutta Medical College Hospital, Calcutta, India, 1905, CFR;[84] (l) Haiti 2010-11, CFR;[85] (m) Philippines 1961-2, in-patients treated with iv fluids, CFR;[86] (n) Cholera Ogawa, Katsina, Nigeria, 1961-2, CFR in hospitalised patients.[87] (o) England, 1849, mortality rates, numbers show deaths (no. of deaths given for age 0-4 combined);[88] (p) Paris, 1832, mortality rates, numbers show deaths;[89] (q) Central Provinces, India, 1875-6, mortality rates, numbers show deaths.[90]

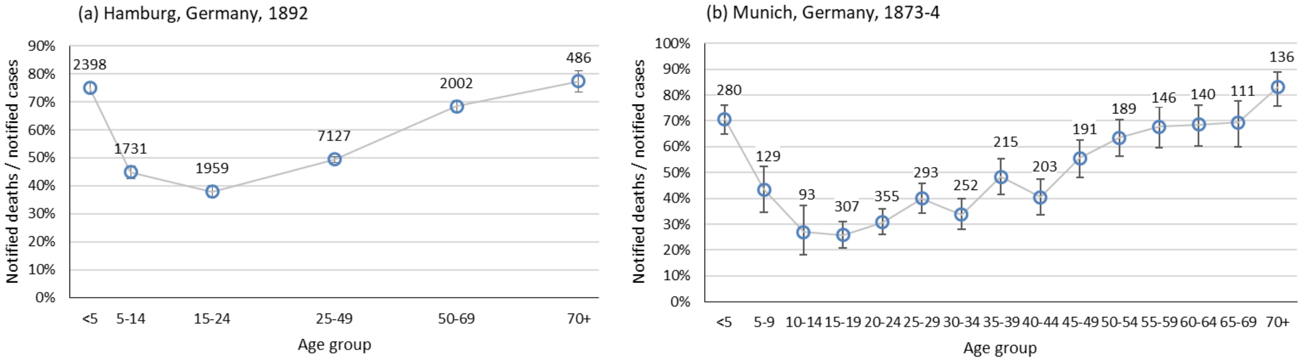

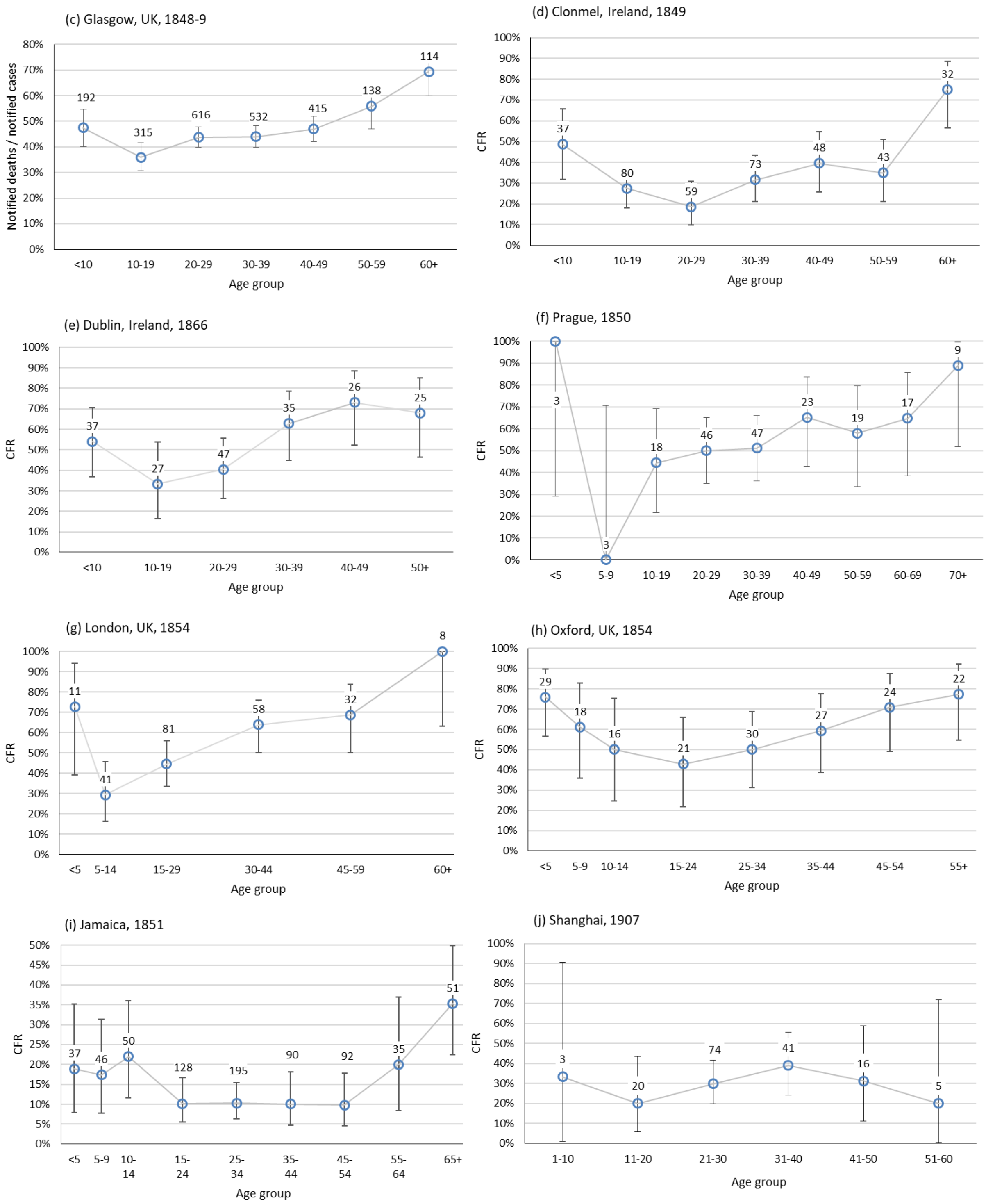
(c) Glasgow, UK, 1848-9
Notified deaths / notified cases
0%
10%
20%
30%
40%
50%
60%
70%
80%
192
315
616
532
415
138
114
<10
10-19
20-29
30-39
40-49
50-59
60+
Age group
(d) Clonmel, Ireland, 1849
CFR
0%
10%
20%
30%
40%
50%
60%
70%
80%
90%
100%
37
80
59
73
48
43
32
<10
10-19
20-29
30-39
40-49
50-59
60+
Age group
(e) Dublin, Ireland, 1866
CFR
37
27
47
35
26
25
<10
10-19
20-29
30-39
40-49
50+
Age group
(f) Prague, 1850
CFR
3
3
18
46
47
23
19
17
9
<5
5-9
10-19
20-29
30-39
40-49
50-59
60-69
70+
Age group
(g) London, UK, 1854
CFR
11
41
81
58
32
8
<5
5-14
15-29
30-44
45-59
60+
Age group
(h) Oxford, UK, 1854
CFR
29
18
16
21
30
27
24
22
<5
5-9
10-14
15-24
25-34
35-44
45-54
55+
Age group
(i) Jamaica, 1851
CFR
0%
5%
10%
15%
20%
25%
30%
35%
40%
45%
50%
37
46
50
128
195
90
92
35
51
<5
5-9
10-14
15-24
25-34
35-44
45-54
55-64
65+
Age group
(j) Shanghai, 1907
CFR
3
20
74
41
16
5
1-10
11-20
21-30
31-40
41-50
51-60
Age group

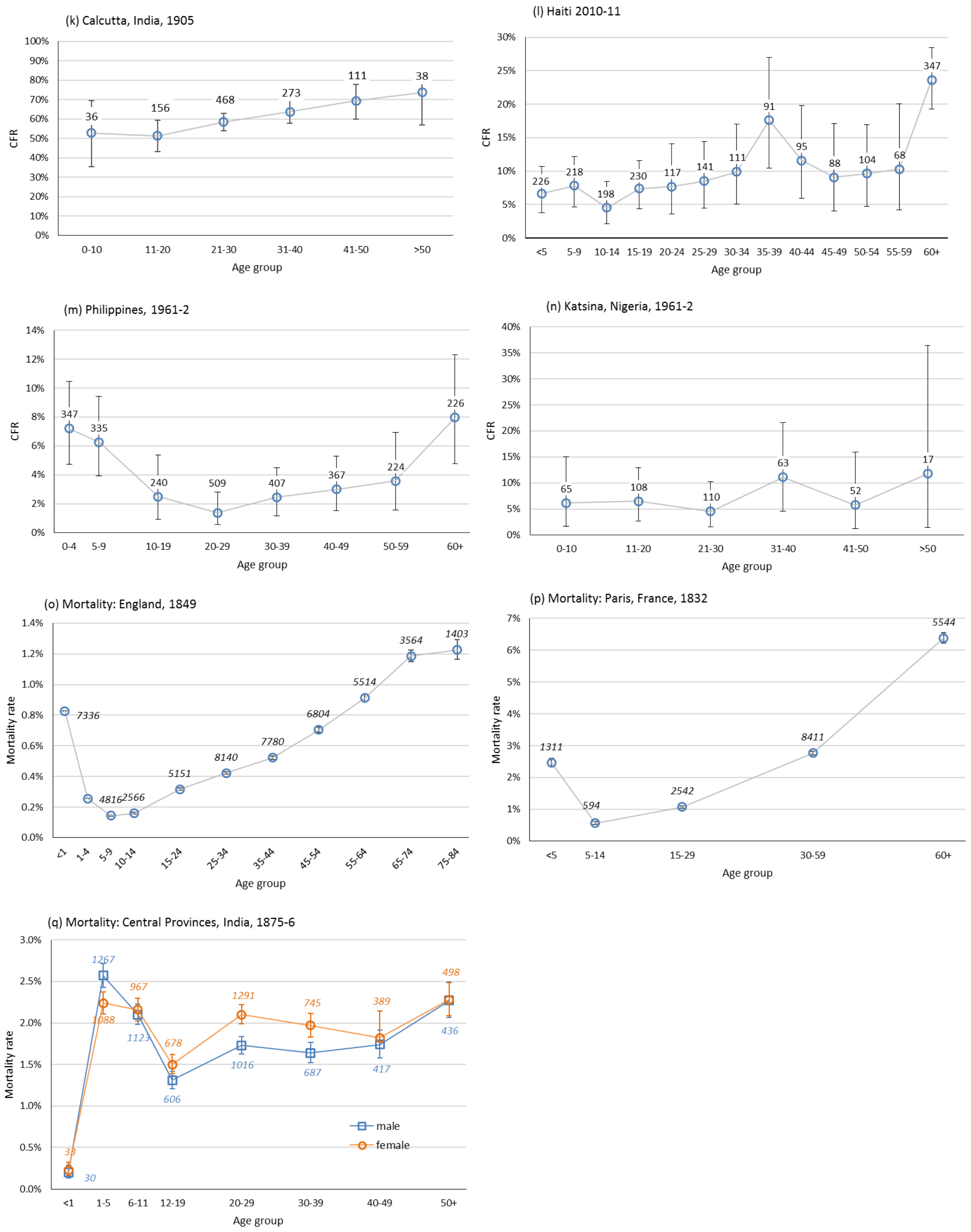
(k) Calcutta, India, 1905
CFR
100%
90%
80%
70%
60%
50%
40%
30%
20%
10%
0%
36
156
468
273
111
38
0-10
11-20
21-30
31-40
41-50
>50
Age group
(l) Haiti 2010-11
CFR
30%
25%
20%
15%
10%
5%
0%
226
218
198
230
117
141
111
91
95
88
104
68
347
<5
5-9
10-14
15-19
20-24
25-29
30-34
35-39
40-44
45-49
50-54
55-59
60+
Age group
(m) Philippines, 1961-2
CFR
14%
12%
10%
8%
6%
4%
2%
0%
347
335
240
509
407
367
224
226
0-4
5-9
10-19
20-29
30-39
40-49
50-59
60+
Age group
(n) Katsina, Nigeria, 1961-2
CFR
40%
35%
30%
25%
20%
15%
10%
5%
0%
65
108
110
63
52
17
0-10
11-20
21-30
31-40
41-50
>50
Age group
(o) Mortality: England, 1849
Mortality rate
1.4%
1.2%
1.0%
0.8%
0.6%
0.4%
0.2%
0.0%
7336
4816
2566
5151
8140
7780
6804
5514
3564
1403
<1
1-4
5-9
10-14
15-24
25-34
35-44
45-54
55-64
65-74
75-84
Age group
(p) Mortality: Paris, France, 1832
Mortality rate
7%
6%
5%
4%
3%
2%
1%
0%
1311
594
2542
8411
5544
<5
5-14
15-29
30-59
60+
Age group
(q) Mortality: Central Provinces, India, 1875-6
Mortality rate
3.0%
2.5%
2.0%
1.5%
1.0%
0.5%
0.0%
1267
967
1088
1123
678
606
1291
1016
745
687
389
417
498
436
33
30
male
female
<1
1-5
6-11
12-19
20-29
30-39
40-49
50+
Age group

### *Lassa fever*

In Nigeria in 2018 the CFR of laboratory-confirmed cases rose with age.[91] The only other studies reporting CFR are hospital-based and show conflicting patterns, with an increase with age in Nigeria[92] but no consistent pattern in Sierra Leone (Figs 3a, S17).[93,94] Most people with Lassa virus infection have mild symptoms so hospitalized and notified cases are not typical.[95]

**Fig S19 Lassa fever** (a) Nigeria, Jan-May 2018, CFR in laboratory-confirmed cases;[91] (b) Irrua specialist teaching hospital, Nigeria, 2011-15, CFR;[92] (c) Sierra Leone, 1977-9, CFR in hospitalised patients;[93] (d) Kenema General Hospital, Sierra Leone, 2008-12, CFR.[94]

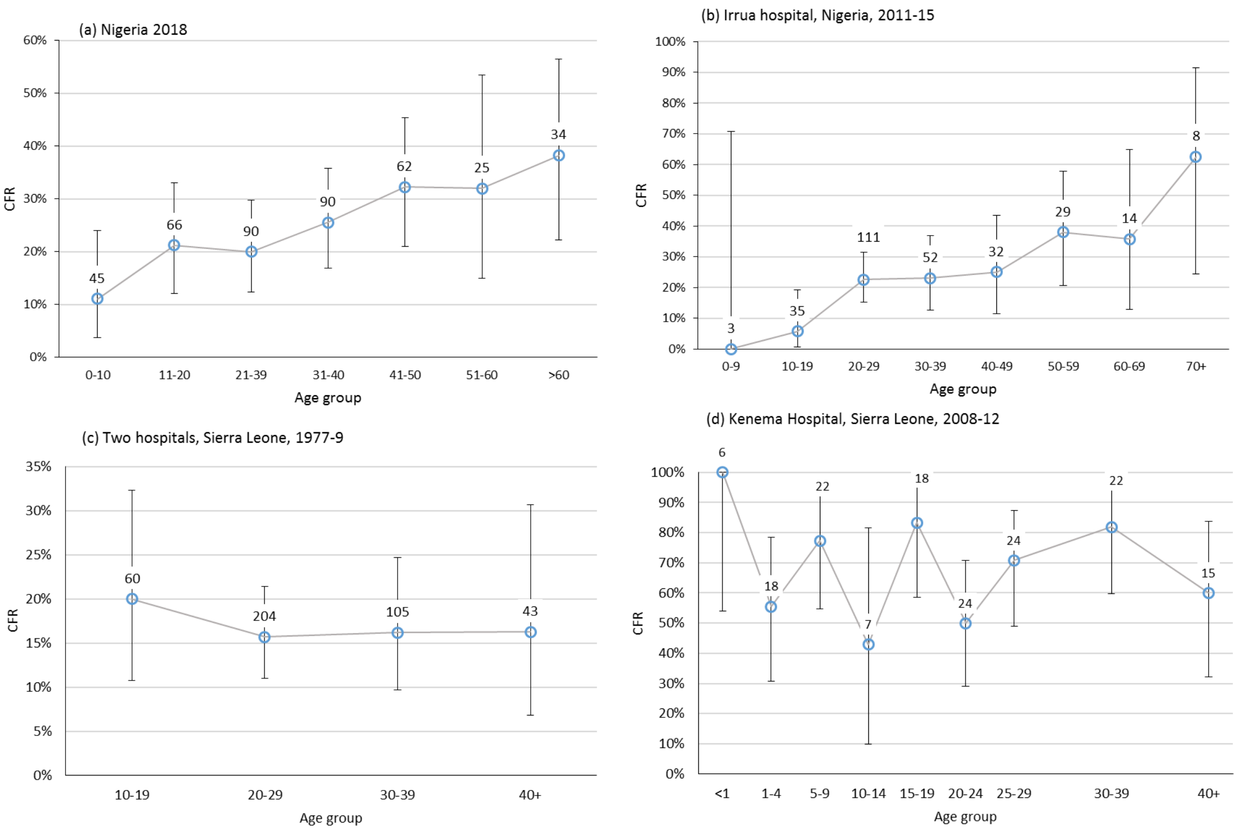

### *Brucellosis*

In Malta in 1936 the CFR from brucellosis was lowest at age 5-9 years (Fig 3d, S20a).[96] In Malta in 1906 the CFR among soldiers' children (age not given) was lower than among the soldiers and their wives (age of wives not given). Among the soldiers the CFR increased with age (Fig S20b).[97]

**Fig S20. Brucellosis** (a) Malta, 1936, CFR;[96] (b) Military and their families, Malta, 1902-5, CFR.[97]

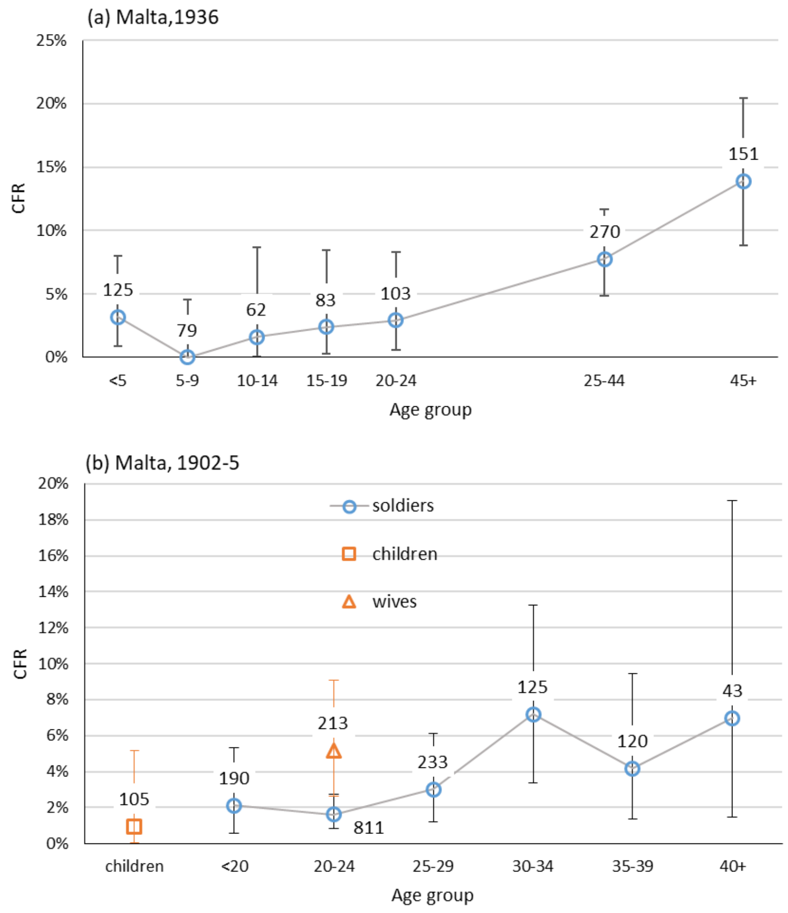

### *Hepatitis B*

With increasing age of infection the risk of symptomatic acute hepatitis increases and the risk of chronic carriage of Hepatitis B surface antigen decreases.[98] The results of a prospective study in Alaska are shown in Figs 3e and S21.[99] Other studies included limited age ranges, but the combined data show a similar pattern of decreasing risk of carriage with increasing age of infection from 0-25 years, and very high carriage rates in the youngest children.[100]

**Fig S21 Hepatitis B** Alaska 1971-6 (a) proportion of new infections with symptomatic hepatitis (b) proportion with chronic Hepatitis B surface antigen carriage[99]

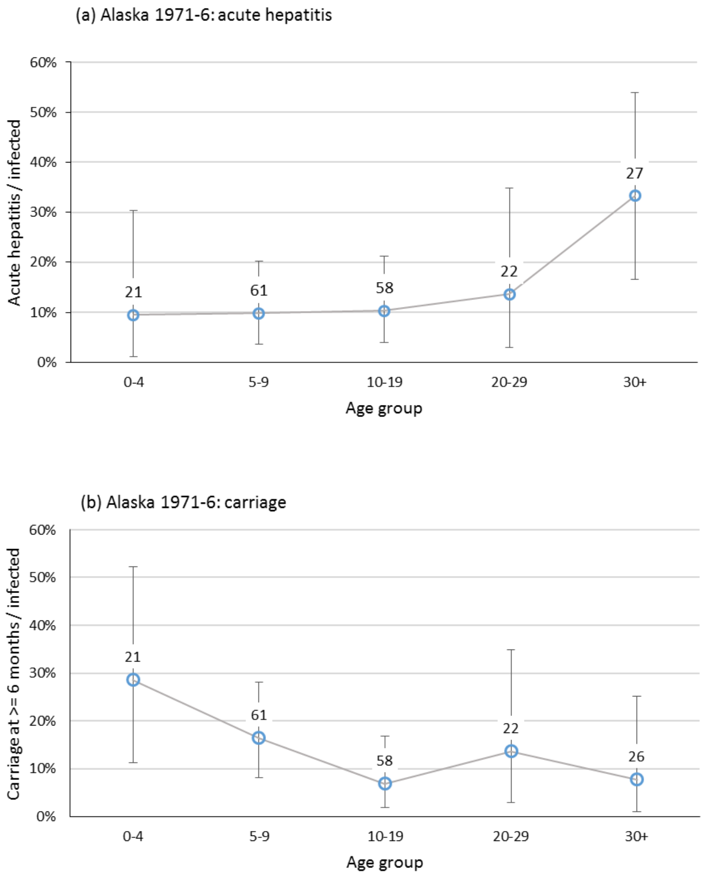

### *Plague*

Most of the available estimates of CFR by age for plague come from around 1900. In the larger outbreaks (India in 1897, Taiwan in 1898)[101,102] the CFR increased with age after age 30 years [102,103] (Fig 3f, S22a,e). In some of the smaller outbreaks there was no clear pattern with age (Fig S22).[104-109]

**Fig S22 Plague** (a) Jalandhar District, India, 75 villages, 1897-8, notified deaths / notified cases;[102] (b) Palanpur District, India, 1897-8, notified deaths / notified cases;[102] (c) Parel Hospital Bombay 1897, CFR;[108] (d) Bombay plague hospitals ~1902, CFR;[105] (e) Taiwan 1898, notified deaths / notified cases;[101] (f) Bubonic plague among Chinese patients in Kennedy Town Hospital, Hong Kong, 1903, CFR;[104] (g) Queensland, Australia, 1900-7, CFR;[103] (h) Natal (non-European population) 1902-3, CFR.[106] (i) Astrakhan, Russia, 1900-1, notified deaths / notified cases;[109] (j) Northeast Brazil, 1939-40, CFR.[107]

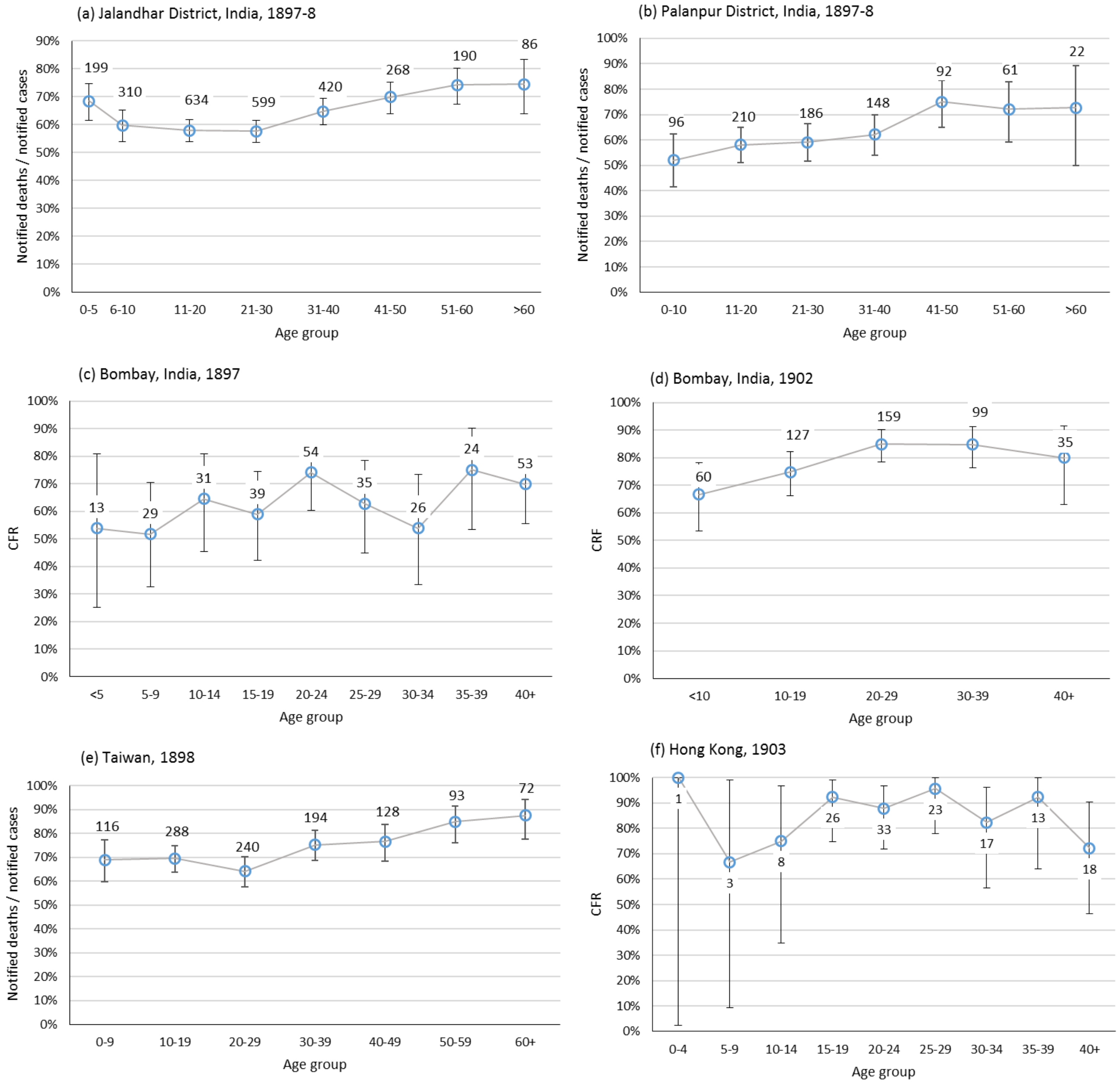

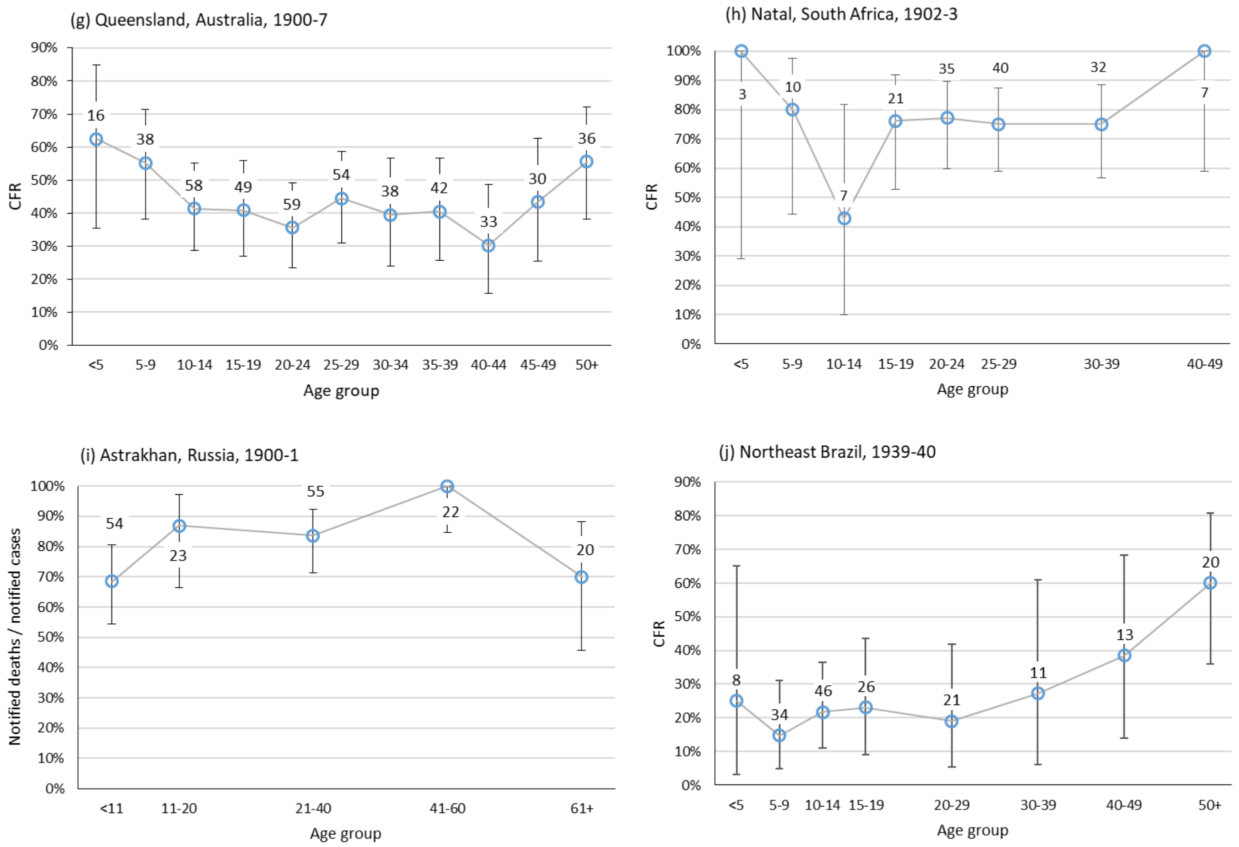
(g) Queensland, Australia, 1900-7
CFR
90%
80%
70%
60%
50%
40%
30%
20%
10%
0%
16
38
58
49
59
54
38
42
33
30
36
<5
5-9
10-14
15-19
20-24
25-29
30-34
35-39
40-44
45-49
50+
Age group
(h) Natal, South Africa, 1902-3
CFR
100%
90%
80%
70%
60%
50%
40%
30%
20%
10%
0%
3
10
7
21
35
40
32
7
<5
5-9
10-14
15-19
20-24
25-29
30-39
40-49
Age group
(i) Astrakhan, Russia, 1900-1
Notified deaths / notified cases
100%
90%
80%
70%
60%
50%
40%
30%
20%
10%
0%
54
23
55
22
20
<11
11-20
21-40
41-60
61+
Age group
(j) Northeast Brazil, 1939-40
CFR
90%
80%
70%
60%
50%
40%
30%
20%
10%
0%
8
34
46
26
21
11
13
20
<5
5-9
10-14
15-19
20-29
30-39
40-49
50+
Age group

### *Hepatitis A*

It is thought that Hepatitis A is more likely to be asymptomatic in childhood but direct evidence is scarce.[110] In England and Wales the CFR based on notifications was lowest between 5 and 34 years (Fig 3g, S23a).[111] Among patients in a referral hospital in Korea there was little difference by age between 10 and 49 years in the proportion with severe hepatitis (defined from the prothrombin time, Fig S23b).[112]

**Fig S23 Hepatitis A** (a) England and Wales 1979-85, notified deaths / notified cases (case numbers estimated from graph);[111] (b) Seoul, Korea, 1996-2010, proportion of hospitalised cases with prolonged prothrombin time in Kyung Hee University Hospital.[112]

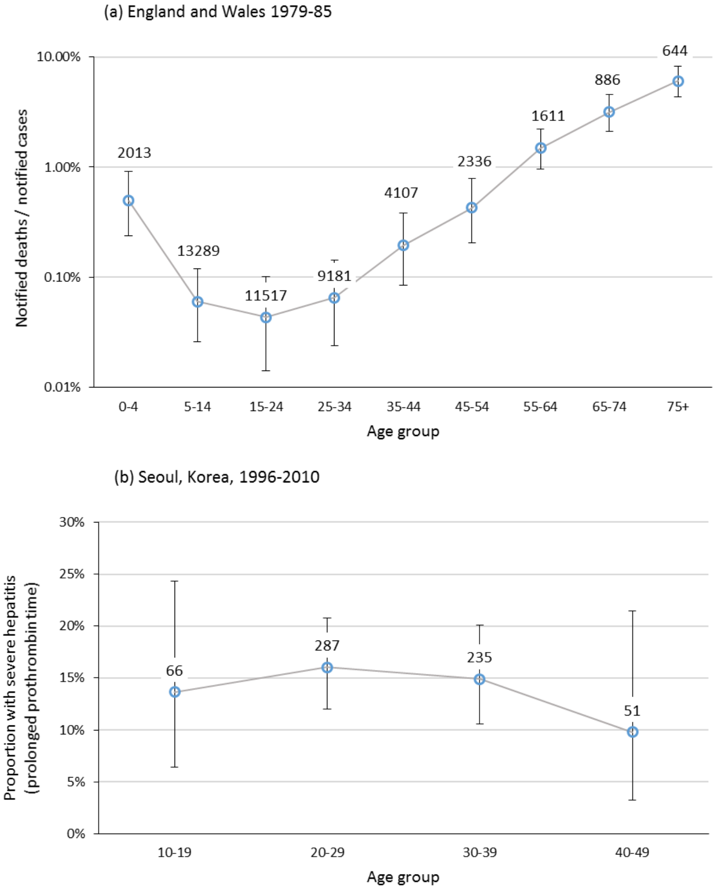

### *Severe acute respiratory syndrome (SARS)*

SARS coronavirus infection was first reported in 2002. Case numbers in children were low, and most published data group children with young adults (Fig S24b).[113] The CFR of SARS increased dramatically with age over 40 years.[113-115] Reports on 135 cases aged <18 years from Canada, Hong Kong, Taiwan and Singapore found no deaths, and children <12 years had milder illness than older children.[116]

**Fig S24 SARS** (a) Hong Kong, 2003, CFR (numbers estimated from incidence data in the paper and population data from UN population estimates);[114] (b) Hong Kong, Beijing and Taiwan, 2003, CFR;[113] (c) China, 2003, CFR (estimated from graph, ~5000 cases).[115]

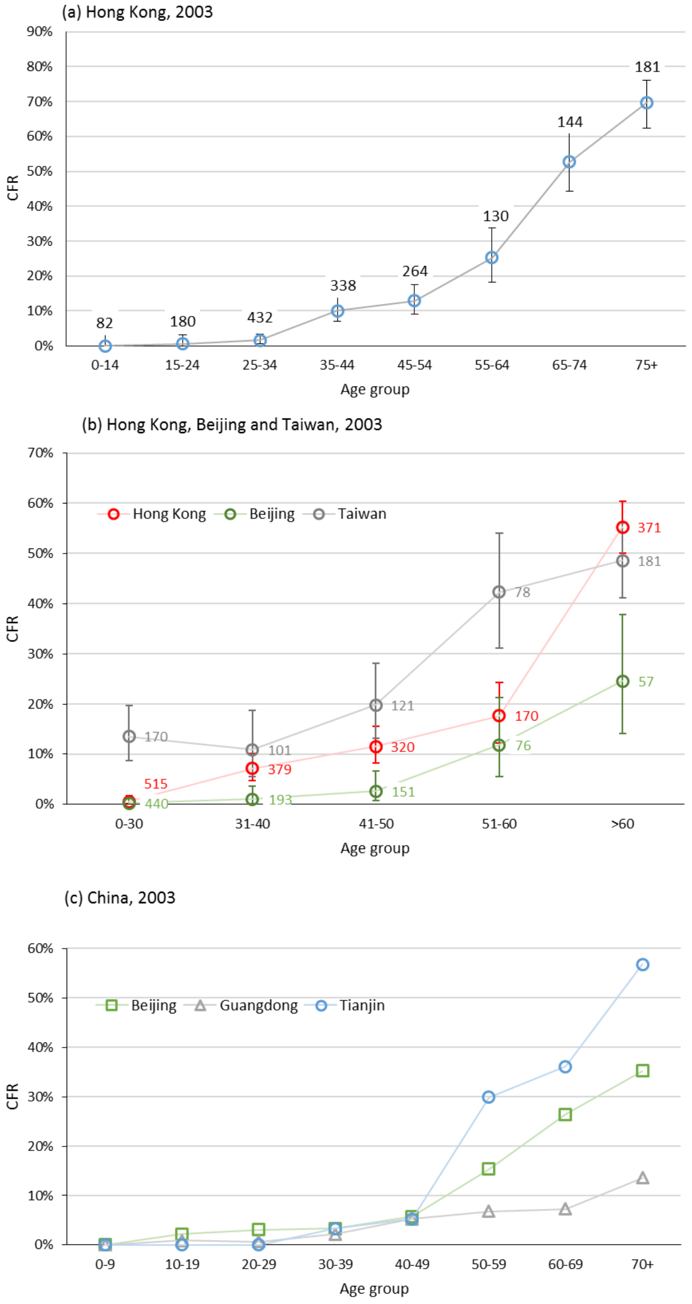

### *COVID-19*

Case fatality rates by age for COVID-19 are available for several settings. It is not always clear who is included, as testing patterns vary between settings and over time (including only more severe cases, or including mild and even asymptomatic cases). While the pandemic is ongoing case fatality rates are often underestimated as some included patients are still ill. Nevertheless, the age-specific patterns in Spain,[117] China,[1] South Korea,[118] The Philippines,[119] Canada,[120] and Italy[121] are remarkably similar, with very few deaths in children and young adults, and slight increases from age 40-49 years, rising very steeply with age thereafter. The data from Mexico[122] show higher rates overall, suggesting that they are more selected, and a slightly earlier rise with age. The interim CFR data of hospitalised patients from New York[123] show a similar pattern with age. Where available, CFR is higher in males than females, but both increase similarly with age.

**Fig 25 COVID-19.** (a) Spain 2020, notified deaths / notified confirmed cases as of 11 May 2020;[117] (b) China 2020, CFR among confirmed cases, outcomes as of 11 February 2020;[1] (c) South Korea 2020, CFR among confirmed cases, outcomes as of 30 March 2020;[118] (d) The Philippines 2020, notified deaths / notified confirmed cases, as of 3 April 2020;[119] (e) Ontario, Canada, CFR among symptomatic confirmed cases and their contacts, outcomes by 15 April 2020;[120] (f) Italy 2020, notified deaths / notified confirmed cases as of 17 March 2020 (case numbers estimated from deaths and CFR so not available for the youngest groups with zero deaths);[121] (g) Mexico 2020, CFR of 7497 confirmed cases (allowing for right censoring) as of 18 April 2020;[122] (h) New York, US, 2020, CFR among confirmed hospital patients, outcomes as of 4 April 2020.[123]

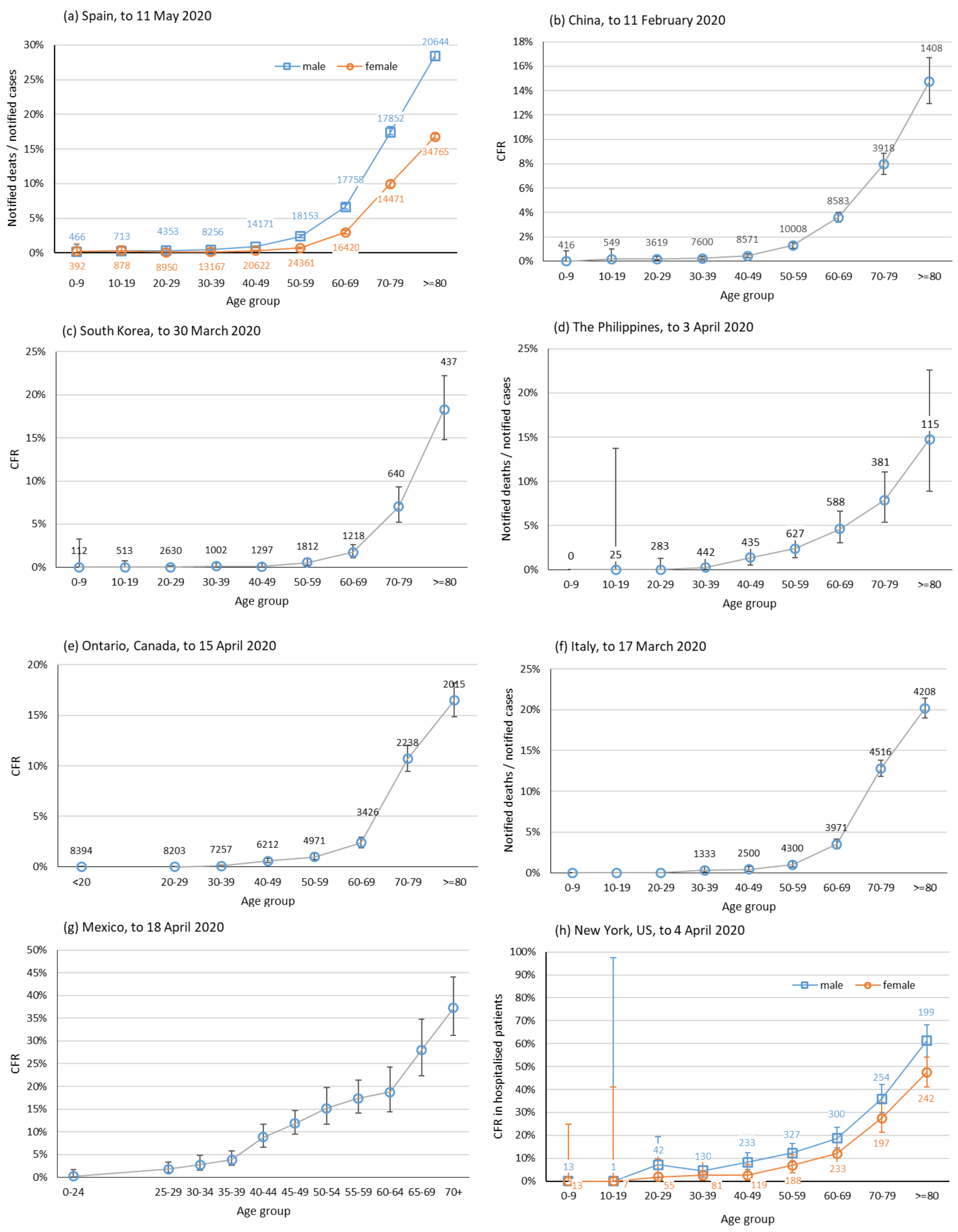
(a) Spain, to 11 May 2020
Notified deats / notified cases
male
female
466 713 4353 8256 14171 18153 17758 17852 20644
392 878 8950 13167 20622 24361 16420 14471 34765
Age group
(b) China, to 11 February 2020
CFR
416 549 3619 7600 8571 10008 8583 3918 1408
Age group
(c) South Korea, to 30 March 2020
CFR
112 513 2630 1002 1297 1812 1218 640 437
Age group
(d) The Philippines, to 3 April 2020
Notified deaths / notified cases
0 25 283 442 435 627 588 381 115
Age group
(e) Ontario, Canada, to 15 April 2020
CFR
8394 8203 7257 6212 4971 3426 2238 2015
Age group
(f) Italy, to 17 March 2020
Notified deaths / notified cases
1333 2500 4300 3971 4516 4208
Age group
(g) Mexico, to 18 April 2020
CFR
Age group
(h) New York, US, to 4 April 2020
CFR in hospitalised patients
male
female
13 1 42 130 233 327 300 254 199
13 7 55 81 119 188 233 197 242
Age group

**Middle East respiratory syndrome (*MERS-CoV)***

Data on confirmed cases of MERS-CoV in Saudi Arabia are available from June 2012-July 2014 and from 2017-18.[124,125] The CFR rises from age 40 years with no clear pattern under this age (Fig 4a, S25). Using the data to July 2014, and the difference in cases found by active and passive case finding, the proportion of infections leading to death was estimated to be lowest at ages 10-19 and 20-29.[124]

**Fig S26 MERS-CoV** (a) Saudi Arabia June 2012-July 2014 and 2017-18, CFR[124,125]

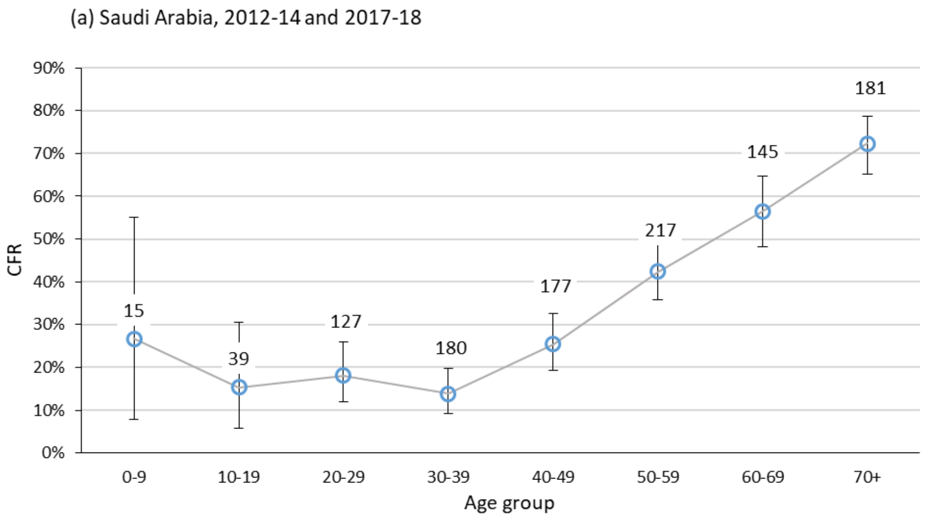

***St Louis encephalitis***

In the first recognised outbreak, in St Louis, US, in 1933, both the incidence and the CFR increased steeply with age from age 40 years (Fig 4b, S26a).[126] Data from later outbreaks and surveillance in the US show a similar pattern (Fig S26b-d).[127-129]

**Fig S27 St Louis Encephalitis** (a) St Louis, US, 1933, CFR,[126] numbers and confidence intervals estimated from data in [130]; (b) US surveillance 1955-71, CFR in cases with known outcome;[128] (c) Houston, Texas, US, 1964, CFR in confirmed and presumptive cases;[127] (d) Dallas, Texas, US, 1966, CFR in confirmed cases.[129]

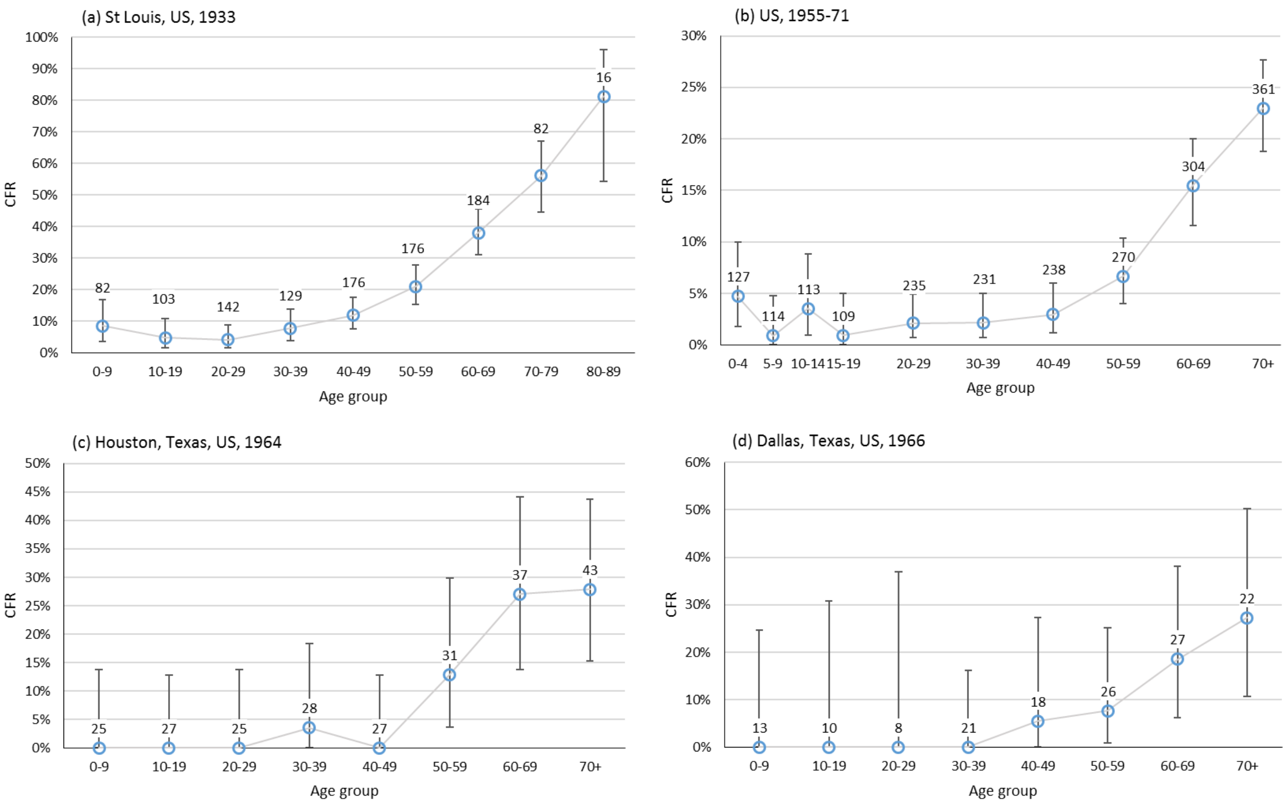

**Campylobacter**

In large studies based on surveillance data the proportion hospitalised (in Canada)[57] and the case fatality rate (in England and Wales)[131] rose with age only from around age 50 years. These studies did not distinguish the different Campylobacter species. In Chelmsford, UK, the proportion of isolates of *Campylobacter enteritidis* that came from hospital patients was higher at the extremes of age.[132] In a milk-borne outbreak of *Campylobacter jejuni* in Aberdeen, UK, the proportion symptomatic fell with age (perhaps reflecting prior immunity).[133]

**Fig S28 Campylobacter** (a) Canada, 2001-4, percent hospitalised (from hospital morbidity database and surveillance data);[57] (b) England and Wales, 1989-2011, CFR (numbers estimated from graph, >1 million cases, 81 deaths);[131] (c) Chelmsford, UK, 1977, proportion of isolates of *Campylobacter enteritidis* from hospitalised patients;[132] (d) *Campylobacter jejuni* Aberdeen, UK, 1979, proportion symptomatic.[133]

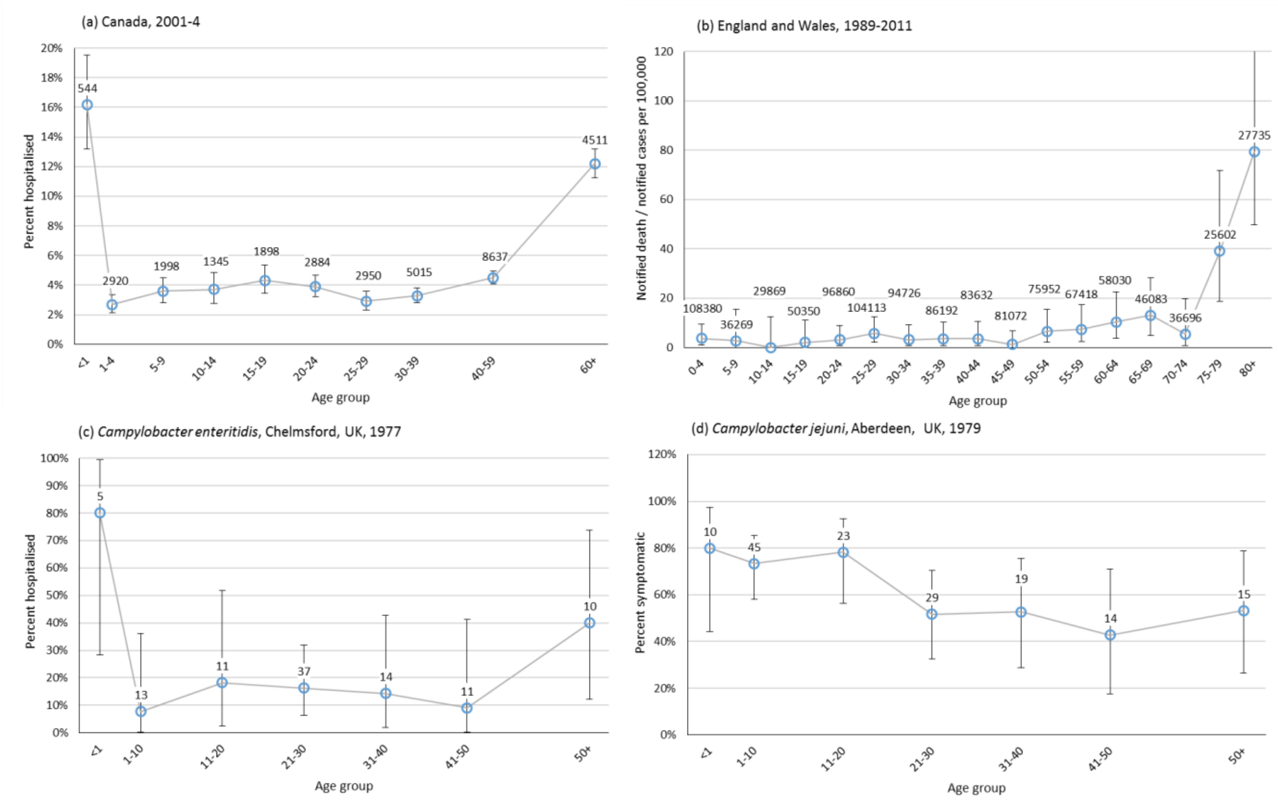

*Western Equine encephalitis*

In an outbreak of Western Equine encephalitis in Canada in 1941, the CFR rose with age only from age 50 (Figs 4c, S27a).[134] Surveillance data from the US, 1955-1971, suggest a rise from age 40 years (S27b).[128]

**Fig S29 Western equine encephalitis** (a) Mannitoba, Canada, 1941, CFR;[134] (b) US surveillance 1955-71, CFR among those with known outcomes.[128]

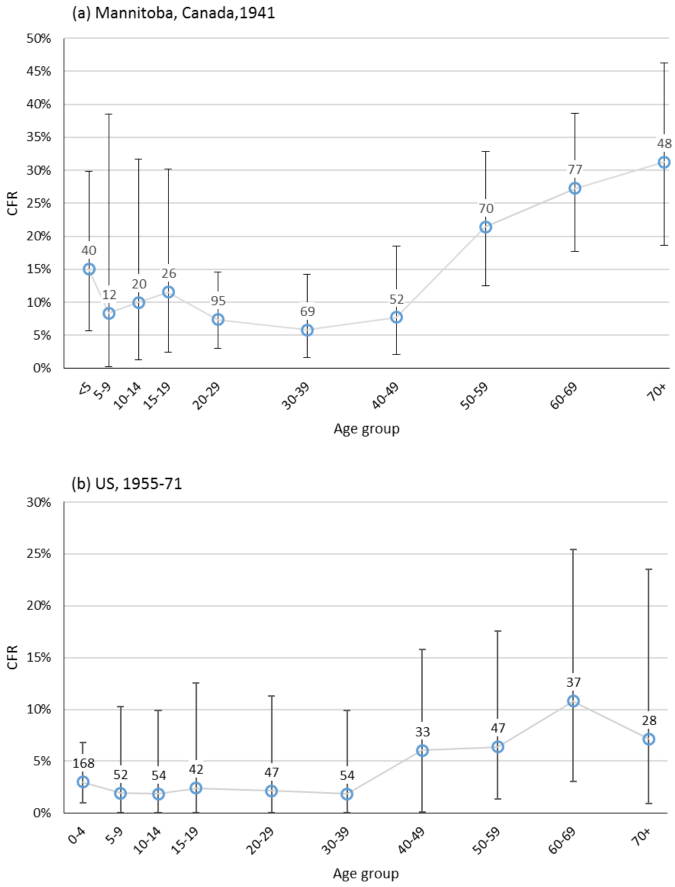

***Diphtheria***

Before vaccination, diphtheria was common in children, with few cases in adults. The CFR among >117,000 patients with diphtheria in London, UK, 1894-1903, decreased throughout childhood and adolescence with the lowest levels in young adults before rising slowly over 35 years (Figs 4d, S28a).[135] A similar pattern was seen in notification data in Pennsylvania in 1907-12 (Fig S28b).[9] CFRs in London hospitals in 1894 (before the use of anit-toxin),[136] in Worcester, UK, in 1896-9,[137] and in small towns in New York State, US, 1915-24[13] dropped with age, with the lowest rates at age 15-19 and ≥20 years (Fig S28c-e).

**Fig S30 Diphtheria** (a) London, UK, 1894-1903, notified deaths / notified cases;[135] (b) Pennsylvania, US, 1907-12, notified deaths, / notified cases;[9] (c) London hospitals, UK, 1894, CFR;[136] (d) Worcester, UK, 1896-99, notified deaths / notified cases.[137] (e) Small towns in New York State, US, 1915-24, notified deaths / notified cases.[13]

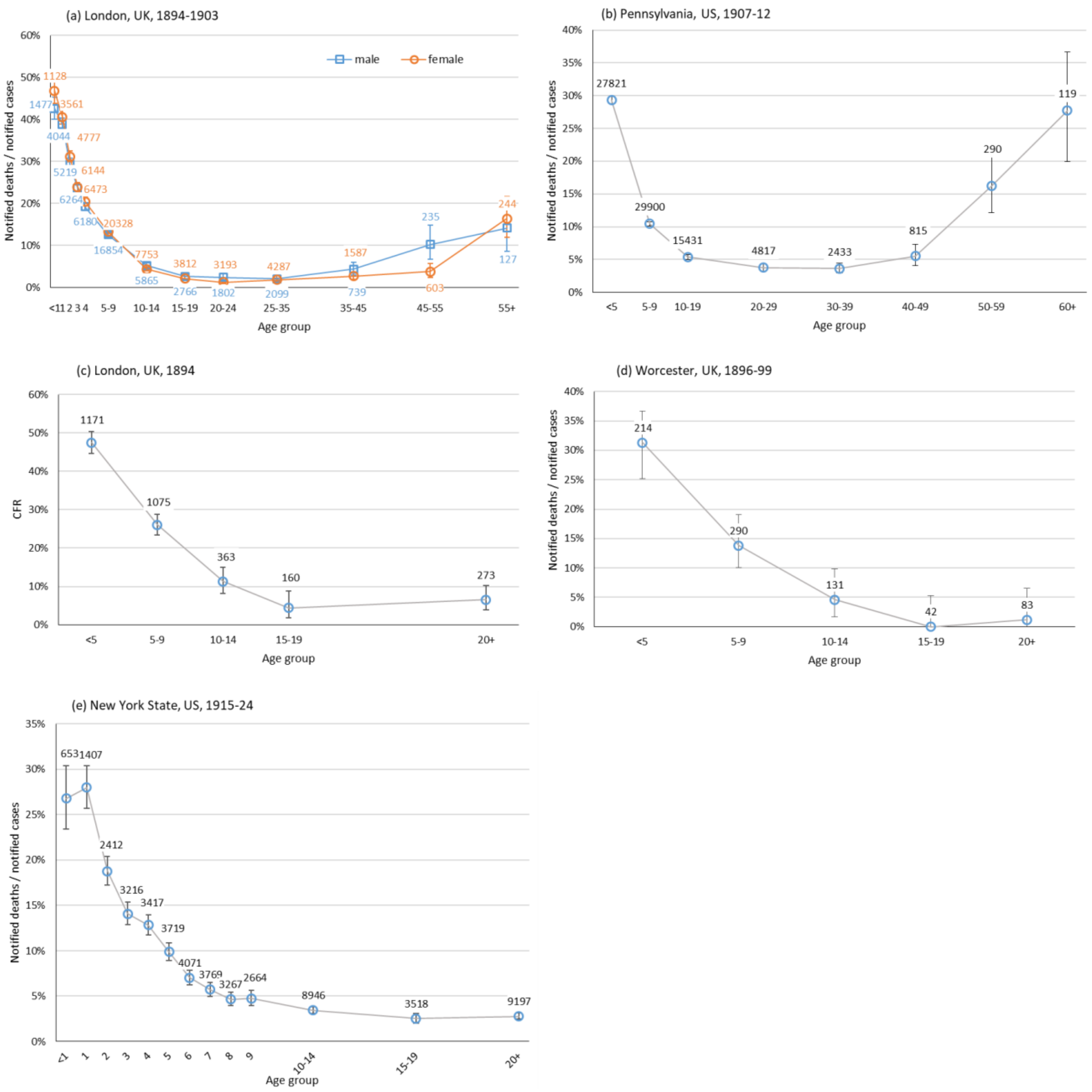

### *Escherichia coli*

In 2011 Germany experienced a large outbreak of enterohaemorrhagic *Escherichia coli* serotype O104:H4.[138] Among 656 patients from Hamburg, the proportion who developed haemolytic uraemic syndrome (HUS) was highest at age 11-20 years and lowest at 40-60 years (Fig 44, S29a). Studies of Shiga-toxin producing *E coli* in the US and UK have grouped data from adults, but also found a higher proportion of haemolytic uraemic syndrome in children than in adults, and in younger than in older children (Fig S29b,c).[139-141] The proportion hospitalised was higher in children than in adults in England (based on information obtained from notified cases),[141] but not in Canada (based on comparison of hospital discharge diagnoses and notification data).[57]

**Fig S31 Escherichia coli:** (a) *E coli* 0104/H4 Hamburg, Germany, 2011, percent developing haemolytic uraemic syndrome;[138] (b) Shiga toxin–producing *E coli* O157 from surveillance sites in US, 2000-2006, percent developing haemolytic uraemic syndrome;[139] (c) Shiga toxin–producing *E coli* in England, 2009-12 from enhanced surveillance data, percent developing HUS and percent hospitalised (approximate data from figures);[141] (d) *E coli* in Canada, 2001-4, hospital recording of enterohaemorrhagic *E coli* (EHEC) / notified cases verotoxigenic *E coli* (VTEC).[57]

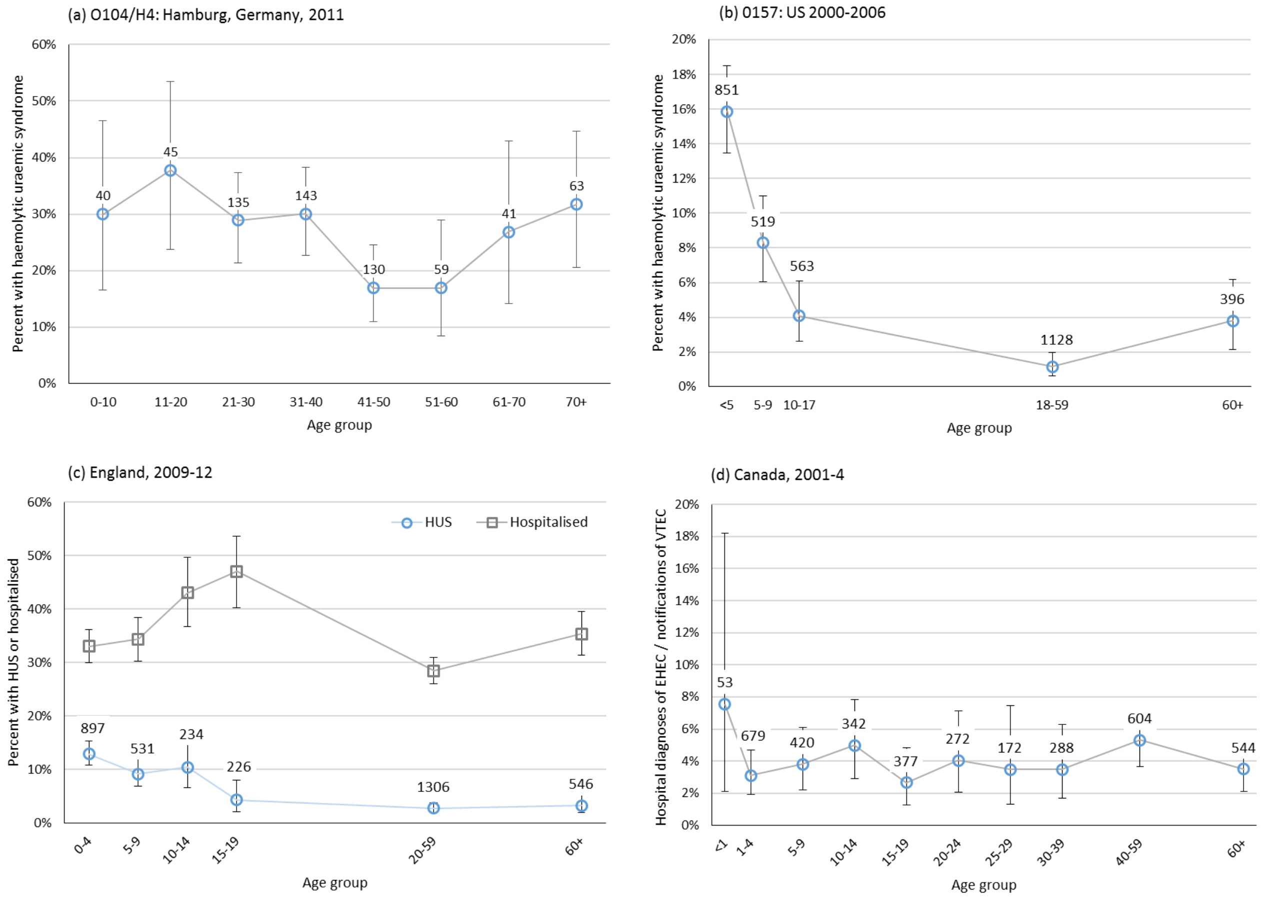

### ***Dengue***

Among nearly 5.5 million notified cases of Dengue in Brazil in 2000-2014, information on severity was available for nearly 4 million and on hospitalisation for 1.4 million.[142] The rates of hospitalisation, of dengue haemorrhagic fever, and of any complications (haemorrhagic fever, dengue shock syndrome, or "complicated dengue" as defined in the Brazilian notification system) were all highest at age 6-10 years, and lowest at 21-35 years, rising again in older adults (Fig 4f, S30a). A study from Cuba used hospital and serosurvey data to estimate the mortality rate among those infected with DENV-2 in 1981, who had previously been infected with DENV-1.[143] The mortality fell with age in children, with no consistent pattern by age thereafter (Fig S30b).

**Fig S32 Dengue** (a) Brazil 2000-2014, proportion haemorrhagic, with any complications, and hospitalised, (number of cases was the same for any complications and haemorrhagic);[142] (b) Cuba 1981, secondary Dengue-2 infection, deaths / estimated number infected (154 deaths and estimated > 800,000 infections).[143]

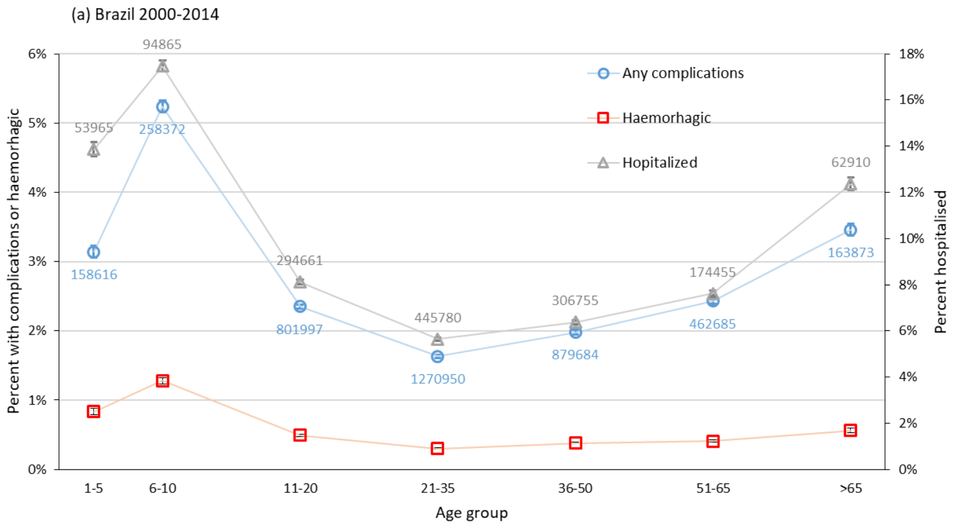


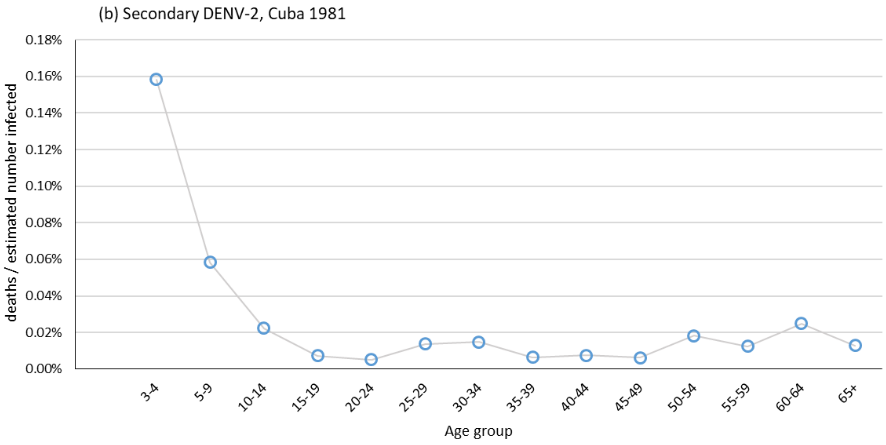

## References


1 The Novel Coronavirus Pneumonia Emergency Response Epidemiology Team. The epidemiological characteristics of an outbreak of 2019 Novel Coronavirus Diseases (COVID-19) - China, 2020. *China CDC Weekly* **2**, 1-10 (2020).
2 Simon, A. K., Hollander, G. A. & McMichael, A. Evolution of the immune system in humans from infancy to old age. *Proc Biol Sci* **282**, 20143085, doi:10.1098/rspb.2014.3085 (2015).
3 Stallybrass, C. O. *The principles of epidemiology and the process of infection*. (George Routledge & Son Ltd, 1931).
4 Ahmed, R., Oldstone, M. B. & Palese, P. Protective immunity and susceptibility to infectious diseases: lessons from the 1918 influenza pandemic. *Nat Immunol* **8**, 1188-1193 (2007).
5 Nathanson, N. & Kew, O. M. From emergence to eradication: the epidemiology of poliomyelitis deconstructed. *Am J Epidemiol* **172**, 1213-1229, doi:10.1093/aje/kwq320 (2010).
6 Department of Health for Scotland. *Poliomyelitis. A survey of the outbreak in Scotland in 1947*. (His Majesty's Stationary Office, 1950).
7 Donovan, C. R. & Bowman, M. Some epidemiological features of poliomyelitis and encephalitis, Manitoba 1941. *Canadian Public Health Journal* **33**, 241-314 (1942).
8 Freyche, M.-J. & Nielsen, J. in *Poliomyelitis (WHO Monograph Series, no. 26)* (World Health Organization, 1955).
9 Lavinder, C. H., Freeman, A. W. & Frost, W. H. Epidemiologic studies of poliomyelitis in New York City and the North Eastern United States during the year 1916. *Public Health Bulletin* **91** (1918).
10 Lumsden, L. L. *Epidemiological studies of poliomyelitis in Kentucky*. (United States Government Printing Office, 1936).
11 Olin, G. in *Poliomyelitis. Papers and discussions presented at the second international poliomyelitis conference* 367-374 (J.B. Lippincott Company, 1952).
12 Logan, W. P. D. Distribution of poliomyelitis by sex, age and geographical area. *Mon Bull Minist Health Public Health Lab Serv* **11**, 147-173 (1952).
13 Godfrey, E. S. The Age Distribution of Communicable Disease According to Size of Community. *Am J Public Health Nations Health* **18**, 616-631 (1928).
14 Picken, R. M. F. One aspect of the transmission of enteric fever. *Public Health* **29**, 2-6 (1915).
15 Murchison, C. Contributions to the etiology of continued fever: or an investigation of various causes which influence the prevalence and mortality of its different forms. *Medico-Chirurgical Transactions* **41**, 219-306 (1858).
16 Davies, S. An epidemic of enteric fever. *Public Health* **8**, 119-124 (1896).
17 Holden, O. M. The Croydon typhoid outbreak. (A summary of the chief clinical features). *Public Health* **52**, 135-146 (1939).
18 Guest Gornall, J. The prevalence of enteric fever in Warrington during 1899. *Public Health* **12**, 841-850 (1900).
19 Porter, C. History of an epidemic of Typhoid fever including consideration of the means of prevention of the disease in Midden towns. *Public Health* **68**, 80-84 (1893).
20 Merrillees, C. R. *Report on typhoid fever in the City of Moorabbin 1943.* (Melbourne, 1943).
21 Lindhardt, M. *The statistics of pulmonary tuberculosis in Denmark 1925-1934. A statistical investigation on the occurrence of pulmonary tuberculosis in the period 1925-1934, worked out on the basis of the Danish National Health Service file of notified cases and deaths.*, (Ejnar Munksgaard, 1939).
22 MacGregor, A. S. M. Studies in the epidemiology of Phthisis. *Public Health* **27**, 269-278 (1924).
23 Picken, R. M. F. Age and sex incidence, as distinct from mortality, in respiratory tuberculosis. *Public Health* **54**, 42-46 (1940).
24 Marais, B. J. *et al.* The clinical epidemiology of childhood pulmonary tuberculosis: a critical review of literature from the pre-chemotherapy era. *Int J Tuberc Lung Dis* **8**, 278-285 (2004).
25 Ferebee, S. H. & Mount, F. W. Tuberculosis morbidity in a controlled trial of the prophylactic use of isoniazid among household contacts. *Am Rev Respir Dis* **85**, 490-510 (1962).

26 Comstock, G. W., Livesay, V. T. & Woolpert, S. F. The prognosis of a positive tuberculin reaction in childhood and adolescence. *Am J Epidemiol* **99**, 131-138 (1974).
27 Panum, P. L. *Observations made during the epidemic of measles on the Faroe Islands in the year 1846*. (Delta Omega Society, 1940).
28 Wilson, G. N. Measles: its prevalence and mortality in Aberdeen. *Public Health* **18**, 65-82 (1905).
29 Ramsay, M. *et al.* The epidemiology of measles in England and Wales: rationale for the 1994 national vaccination campaign. *Commun Dis Rep CDR Rev* **4**, R141-146 (1994).
30 Institutul National de Sanatate Publica Romania. http://www.cnscbt.ro/index.php/informari-saptamanale/rujeola-1/1071-situatia-deceselor-datorate-rujeolei-romania-2016-2019/file (2019).
31 Institutul National de Sanatate Publica Romania. http://www.cnscbt.ro/index.php/informari-saptamanale/rujeola-1/1143-situatia-rujeolei-in-romania-la-data-de-01-03-2019/file (2019).
32 Muscat, M. *et al.* The measles outbreak in Bulgaria, 2009-2011: An epidemiological assessment and lessons learnt. *Euro surveill* **21**, 30152, doi:10.2807/1560-7917.ES.2016.21.9.30152 (2016).
33 Woudenberg, T. *et al.* Large measles epidemic in the Netherlands, May 2013 to March 2014: changing epidemiology. *Euro surveill* **22**, doi:10.2807/1560-7917.ES.2017.22.3.30443 (2017).
34 Barry, D. *Report on an epidemic of small-pox at Sheffield during 1887-88*. (HMSO, 1889).
35 Marson, J. F. in *General Board of Health. Papers relating to the history and practice of vaccination* (ed J. Simon) (HMSO, 1857).
36 Killinck Mallard, C. The Leicester Method of dealing with small-pox. *Public Health* **16**, 607-629 (1904).
37 Dingle, C. V. The story of the Middlesborough small-pox epidemic and some of its lessons. *Public Health* **11**, 173-192 (1898).
38 Niven, J. Small-pox problems. *Public Health* **64** (1893).
39 Hill, A. The small-pox experience of Birmingham 1893-5. *Public Health* **8**, 413-414 (1896).
40 Boelle, P. Y. & Hanslik, T. Varicella in non-immune persons: incidence, hospitalization and mortality rates. *Epidemiol Infect* **129**, 599-606 (2002).
41 Guess, H. A., Broughton, D. D., Melton, L. J., 3rd & Kurland, L. T. Population-based studies of varicella complications. *Pediatrics* **78**, 723-727 (1986).
42 Brisson, M. *et al.* Epidemiology of varicella zoster virus infection in Canada and the United Kingdom. *Epidemiol Infect* **127**, 305-314 (2001).
43 Henke, C. E., Kurland, L. T. & Elveback, L. R. Infectious mononucleosis in Rochester, Minnesota, 1950 through 1969. *Am J Epidemiol* **98**, 483-490 (1973).
44 Collaborative group on AIDS incubation and HIV survival including the CASCADE EU Concerted Action. Time from HIV-1 seroconversion to AIDS and death before widespread use of highly-active antiretroviral therapy: a collaborative re-analysis. *Lancet* **355**, 1131-1137 (2000).
45 Darby, S. C., Ewart, D. W., Giangrande, P. L., Spooner, R. J. & Rizza, C. R. Importance of age at infection with HIV-1 for survival and development of AIDS in UK haemophilia population. UK Haemophilia Centre Directors' Organisation. *Lancet* **347**, 1573-1579 (1996).
46 Taubenberger, J. K. & Morens, D. M. 1918 Influenza: the mother of all pandemics. *Emerg Infect Dis* **12**, 15-22 (2006).
47 Frost, W. H. & Sydenstricker, E. Influenza in Maryland. Preliminary statistics of certain localities. *Public Health Reports* **34**, 491-504 (1919).
48 Collins, S. D. Age and sex incidence of influenza and pneumonia morbidity and mortality in the epidemic of 1928-29 with comparative data for the epidemic of 1918-19. *Public Health Reports* **46**, 1909-1937 (1931).
49 Chowell, G., Simonsen, L., Flores, J., Miller, M. A. & Viboud, C. Death patterns during the 1918 influenza pandemic in Chile. *Emerg Infect Dis* **20**, 1803-1811 (2014).
50 Chowell, G. *et al.* The 1918-19 influenza pandemic in Boyaca, Colombia. *Emerg Infect Dis* **18**, 48-56 (2012).

51 Chowell, G. *et al.* The 1918-1920 influenza pandemic in Peru. *Vaccine* **29 Suppl 2**, B21-26, doi:10.1016/j.vaccine.2011.02.048 (2011).
52 Mamelund, S. E. Geography may explain adult mortality from the 1918-20 influenza pandemic. *Epidemics* **3**, 46-60 (2011).
53 McMorrow, M. L. *et al.* Severe Acute Respiratory Illness Deaths in Sub-Saharan Africa and the Role of Influenza: A Case Series From 8 Countries. *J Infect Dis* **212**, 853-860 (2015).
54 Laing, J. S. & Hay, M. Whooping cough: its prevalence and mortality in Aberdeen. *Public Health* **14**, 584- (1902).
55 Gil Prieto, R., Alejandre, C. G., Meca, A. A., Barrera, V. H. & de Miguel, A. G. Epidemiology of hospital-treated Salmonella infection; data from a national cohort over a ten-year period. *J Infect* **58**, 175-181 (2009).
56 Chen, P. L. *et al.* Epidemiology, disease spectrum and economic burden of non-typhoidal Salmonella infections in Taiwan, 2006-2008. *Epidemiol Infect* **140**, 2256-2263 (2012).
57 Ruzante, J. M., Majowicz, S. E., Fazil, A. & Davidson, V. J. Hospitalization and deaths for select enteric illnesses and associated sequelae in Canada, 2001-2004. *Epidemiol Infect* **139**, 937-945 (2011).
58 Wilson, H. L., Kennedy, K. J. & Moffatt, C. R. M. Epidemiology of non-typhoid Salmonella infection in the Australian Capital Territory over a 10-year period. *Intern Med J* **48**, 316-323 (2018).
59 Fisker, N., Vinding, K., Molbak, K. & Hornstrup, M. K. Clinical review of nontyphoid Salmonella infections from 1991 to 1999 in a Danish county. *Clin Infect Dis* **37**, e47-52, doi:10.1086/375897 (2003).
60 Le Bacq, F., Louwagie, B. & Verhaegen, J. Salmonella typhimurium and Salmonella enteritidis: changing epidemiology from 1973 until 1992. *Eur J Epidemiol* **10**, 367-371 (1994).
61 Bille, B., Mellbin, T. & Nordbring, F. An Extensive Outbreak of Gastroenteritis Caused by Salmonella Newport. I. Some Observations of 745 Known Cases. *Acta Med Scand* **175**, 557-567 (1964).
62 Procter, J. R. Notes on the Yellow Fever epidemic at Hickman, Ky., 1878. (Frankfort, 1879).
63 Sternberg, G. M. *Report on the etiology and prevention of yellow fever*. (Government Printing Office, 1890).
64 Woods, H. M. *Epidemiological study of scarlet fever in England and Wales since 1900. Medical Research Council Special Report Series No. 180* (His Majesty's Stationary Office, London, 1933).
65 Anon. The case of mortality (fatality) of scarlet fever. *Public Health*, 333-334 (1895).
66 WHO Ebola Response Team. Ebola Virus Disease among Male and Female Persons in West Africa. *N Engl J Med* **374**, 95-96 (2016).
67 Bower, H. *et al.* Exposure-specific and age-specific attack rates for Ebola virus disease in Ebola-affected households, Sierra Leone. *Emerg Infect Dis* **22**, 1403-1412 (2016).
68 Glynn, J. R. *et al.* Asymptomatic infection and unrecognised Ebola Virus Disease: seroprevalence of antibodies to Ebola virus in a large cross-sectional study in Ebola-affected households, Sierra Leone, using a new non-invasive assay. *Lancet Infect Dis* **17**, 645-653 (2017).
69 Bower, H. *et al.* Deaths, late deaths, and role of infecting dose in Ebola virus disease in Sierra Leone: retrospective cohort study. *BMJ* **353**, i2403, doi:10.1136/bmj.i2403 (2016).
70 Williamson, G. A. *Report on the outbreak of epidemic cerebro-spinal meningitis, December 1908 to May 1909*, (Publisher not identified, 1909).
71 Dickie, D. *Transactions of the twenty-seventh annual conference of state and territorial health officers with the United States Public Health Service. Public Health Bulletin No. 194* (United States Government Printing Office, Washington, 1930).
72 Heiman, H. & Feldstein, S. *Meningococcus meningitis*. (JB Lippincott Company, 1913).
73 Kono, R. & Kim, K. H. Comparative epidemiological features of Japanese encephalitis in the Republic of Korea, China (Taiwan) and Japan. *Bull World Health Organ* **40**, 263-277 (1969).
74 Kumar Pant, D., Tenzin, T., Chand, R., Kumar Sharma, B. & Raj Bist, P. Spatio-temporal epidemiology of Japanese encephalitis in Nepal, 2007-2015. *PLoS ONE* **12**, e0180591 (2017).

75 Reincke. in *The Local Government Board. Reports and Papers on Cholera in England in 1893.* (Her Majesty's Stationary Office, 1894).
76 Sticker, G. *Abhandlungen aus der Seuchengeschichte und Seuchenlehre. II. Band: Die Cholera*. (A. Töpelmann, 1912).
77 Sibley, S. W. *Report on the cholera patients admitted into the hospital during the year 1854*. (James Truscott, 1855).
78 Acland, H. W. *Memoir on the cholera at Oxford in the year 1854 with considerations suggested by the epidemic*. (John Churchill, 1856).
79 Mahoney, O. B. *Pathological and practial treatise on epidemic cholera, its history , causes, various forms, and treatment*. (John Churchill, 1853).
80 Hayden, T. & Cruise, F. R. *Report on the cholera epidemic of 1866: as treated in the Mater Misericordiae Hospital, Dublin; with general remarks on the disease*. (Fannin and Company, 1867).
81 Finger, D. *Die Cholera Epidemica nach Beobachtungen.* (Herman Fritzsche, Leipzig, 1851).
82 Parkin, J. *Statistical report of the epidemic cholera in Jamaica*. (William H Allen, 1852).
83 Cox, S. M. *Report on an outbreak of asiatic cholera in Shanghai during the summer of 1907*. (Methodist Publishing House, 1908).
84 Rogers, L. *Cholera and its treatment*. (Oxford University Press, 1911).
85 Page, A. L. *et al.* Geographic distribution and mortality risk factors during the cholera outbreak in a rural region of Haiti, 2010-2011. *PLoS Negl Trop Dis* **9**, e0003605, doi:10.1371/journal.pntd.0003605 (2015).
86 Dizon, J. J. *et al.* Studies of cholera El Tor in the Philippines. I. Characteristics of cholera El Tor in Negros Occidental Province, November 1961 to September 1962. *Bull World Health Organ* **33**, 627-636 (1965).
87 Umoh, J. U., Adesiyun, A. A., Adekeye, J. O. & Nadarajah, M. Epidemiological features of an outbreak of gastroenteritis/cholera in Katsina, Northern Nigeria. *J Hyg (Lond)* **91**, 101-111 (1983).
88 Gull, W. W. in *Reports on epidemic cholera drawn up at the desire of the cholera committee of The Royal College of Physicians* (eds W. Baly & W. W. Gull) (John Churchill, 1854).
89 Anon. *Report on the cholera in Paris*. (Samuel S & William Wood, 1849).
90 Townsend, S. C. *Report on the epidemic of cholera of 1875.1876 in the Central Provinces*. (Chief Commissioner's Office Press, 1878).
91 Ilori, E. A. *et al.* Epidemiologic and Clinical Features of Lassa Fever Outbreak in Nigeria, January 1-May 6, 2018. *Emerg Infect Dis* **25** (2019).
92 Okokhere, P. *et al.* Clinical and laboratory predictors of Lassa fever outcome in a dedicated treatment facility in Nigeria: a retrospective, observational cohort study. *Lancet Infect Dis* **18**, 684-695 (2018).
93 McCormick, J. B. *et al.* A case-control study of the clinical diagnosis and course of Lassa fever. *J Infect Dis* **155**, 445-455 (1987).
94 Shaffer, J. G. *et al.* Lassa fever in post-conflict sierra leone. *PLoS Negl Trop Dis* **8**, e2748, doi:10.1371/journal.pntd.0002748 (2014).
95 McCormick, J. B., Webb, P. A., Krebs, J. W., Johnson, K. M. & Smith, E. S. A prospective study of the epidemiology and ecology of Lassa fever. *J Infect Dis* **155**, 437-444 (1987).
96 Debono, J. E. in *Brucellosis in Man and Animals* (ed I. Forest Huddleson) (The Commonwealth Fund, 1939).
97 McCulloch, T. & Weir, J. C. in *Reports of the Commission appointed by the Admirality, the War Office, and the Civil Government of Malta, for thei investigation of Mediterranean Fever, under the supervision of an advisory committee of The Royal Society. Part VII* (Harrison and Sons, 1907).
98 Pearce, N., Milne, A. & Moyes, C. Hepatitis B virus: the importance of age at infection. *N Z Med J* **101**, 788-790 (1988).
99 McMahon, B. J. *et al.* Acute hepatitis B virus infection: relation of age to the clinical expression of disease and subsequent development of the carrier state. *J Infect Dis* **151**, 599-603 (1985).

100 Edmunds, W. J., Medley, G. F., Nokes, D. J., Hall, A. J. & Whittle, H. C. The influence of age on the development of the hepatitis B carrier state. *Proc Biol Sci* **253**, 197-201 (1993).
101 Benedict, C. *Bubonic plague in nineteenth century China. PhD thesis.* (UMI, 1992).
102 Sticker, G. *Abhandlungen aus der Seuchengeschichte und Seuchenlehre. I. Band: Die Pest*. (A. Töpelmann, 1910).
103 Burnet Ham, B. *Report on Plague in Queensland, 1900-1907*. (Department of Health, 1907).
104 Anon. *Plague cases treated in the Kennedy Town Hospital, Hong Kong.* (Govenrment Report, 1903).
105 Choksy, N. H. *The treatment of plague with Prof. Lustig's serum*. (Eagle Printing Office, 1903).
106 Hill, E. *Report on the plague in Natal 1902-3*. (Cassell and Company, 1904).
107 Macchiavello, A. *Contribuciones al estudio de la peste bubonica en el nordeste del Brasil*. (Oficina Sanitaria Panamericana, 1941).
108 Thomson, G. S. & Thomson, J. *A treatise on plague. The conditions for its causation, prevalence, incidence, immunity, prevention, and treatment*. (Swan Sonnenschein and Co. Lim., 1901).
109 Wu, L. T. *A treatise on pneumonic plague*. (League of Nations, 1926).
110 Lednar, W. M. *et al.* Frequency of illness associated with epidemic hepatitis A virus infections in adults. *Am J Epidemiol* **122**, 226-233 (1985).
111 Forbes, A. & Williams, R. Increasing age--an important adverse prognostic factor in hepatitis A virus infection. *J R Coll Physicians Lond* **22**, 237-239 (1988).
112 Shim, J. J., Chin, S. O., Lee, C. K., Jang, J. Y. & Kim, B. H. Epidemiological changes in hepatitis A in Korea: increasing age and its effect on clinical outcomes. *Epidemiol Infect* **140**, 2182-2189 (2012).
113 Lau, E. H. *et al.* A comparative epidemiologic analysis of SARS in Hong Kong, Beijing and Taiwan. *BMC Infect Dis* **10**, 50, doi:10.1186/1471-2334-10-50 (2010).
114 Chan-Yeung, M. & Xu, R. H. SARS: epidemiology. *Respirology* **8 Suppl**, S9-14 (2003).
115 Cao, W. C., de Vlas, S. J. & Richardus, J. H. The severe acute respiratory syndrome epidemic in mainland China dissected. *Infect Dis Rep* **3**, e2, doi:10.4081/idr.2011.e2 (2011).
116 Stockman, L. J. *et al.* Severe acute respiratory syndrome in children. *Pediatr Infect Dis J* **26**, 68-74 (2007).
117 Centro de Coordinación de Alertas y Emergencias Sanitarias. *Actualización nº 103. Enfermedad por el coronavirus (COVID-19). 12.05.2020 (datos consolidados a las 21:00 horas del 11.05.2020) Situación en España.* https://www.mscbs.gob.es/profesionales/saludPublica/ccayes/alertasActual/nCov-China/documentos/Actualizacion_103_COVID-19.pdf (2020).
118 The Government of the Republic of Korea. *Tackling COVID-19. Health, quarantine and economic measures: Korean experience.* http://ncov.mohw.go.kr/upload/viewer/skin/doc.html?fn=1588831612877_20200507150653.pdf&rs=/upload/viewer/result/202005/ (2020).
119 Alipio, M. M. & Pregoner, J. D. M. Epidemiological characteristics of an outbreak of Coronavirus Disease 2019 in the Philippines. Preprint at https://www.medrxiv.org/content/10.1101/2020.04.12.20053926v1 (2020).
120 Bignami-Van Assche, S., Ghio, D. & Van Assche, A. Estimates of COVID-19 case-fatality risk from individual-level data. Preprint at https://www.medrxiv.org/content/10.1101/2020.04.16.20067751v1 (2020).
121 Onder, G., Rezza, G. & Brusaferro, S. Case-Fatality Rate and Characteristics of Patients Dying in Relation to COVID-19 in Italy. *JAMA*, doi:10.1001/jama.2020.4683 (2020).
122 Solis, P. & Carreño, H. COVID-19 Fatality and Comorbidity Risk Factors among Diagnosed Patients in Mexico. Preprint at https://www.medrxiv.org/content/10.1101/2020.04.21.20074591v1 (2020).
123 Richardson, S. *et al.* Presenting Characteristics, Comorbidities, and Outcomes Among 5700 Patients Hospitalized With COVID-19 in the New York City Area. *JAMA*, doi:10.1001/jama.2020.6775 (2020).

124 Lessler, J. *et al.* Estimating the Severity and Subclinical Burden of Middle East Respiratory Syndrome Coronavirus Infection in the Kingdom of Saudi Arabia. *Am J Epidemiol* **183**, 657-663 (2016).
125 World_Health_Organization. *MERS-CoV Disease outbreak news (listings for 1 November 2018, 3 October 2018, 18 June 2018, 26 January 2018)* http://www.who.int/csr/don/archive/disease/coronavirus_infections/en/ (2018).
126 US Treasury Department Public Health Service. *Report on the St. Louis outbreak of encephalitis. Public Health Bulletin No. 214.* (Washington, 1935).
127 Luby, J. P. *et al.* The epidemiology of St. Louis encephalitis in Houston, Texas, 1964. *Am J Epidemiol* **86**, 584-597 (1967).
128 McGowan, J. E., Jr., Bryan, J. A. & Gregg, M. B. Surveillance of arboviral encephalitis in the United States, 1955-1971. *Am J Epidemiol* **97**, 199-207 (1973).
129 Hopkins, C. C. *et al.* The epidemiology of St. Louis encephalitis in Dallas, Texas, 1966. *Am J Epidemiol* **102**, 1-15 (1975).
130 Muckenfuss, R. S. Clinical Observations and Laboratory Investigations on the 1933 Epidemic of Encephalitis in St. Louis. *Bull N Y Acad Med* **10**, 444-453 (1934).
131 Nichols, G. L., Richardson, J. F., Sheppard, S. K., Lane, C. & Sarran, C. Campylobacter epidemiology: a descriptive study reviewing 1 million cases in England and Wales between 1989 and 2011. *BMJ Open* **2**, doi:10.1136/bmjopen-2012-001179 (2012).
132 Bradshaw, M. J., Brown, R., Swallow, J. H. & Rycroft, J. A. Campylobacter enteritis in Chelmsford. *Postgrad Med J* **56**, 80-84 (1980).
133 Porter, I. A. & Reid, T. M. A milk-borne outbreak of Campylobacter infection. *J Hyg (Lond)* **84**, 415-419 (1980).
134 Donovan, C. R. & Bowman, M. Epidemiology of Encephalitis: Western Equine Type, Manitoba, 1941. *Can Med Assoc J* **46**, 525-530 (1942).
135 Ramsey Smith, W. Data from Albutt's System of Medicine compiled from County of London Records of Diphtheria. In *The official year-book of the Commonwealth of Australia* no.16 (1923).
136 Anon. *Report of the Medical Superintendents upon the use of antitoxic serum in the treatment of diphtheria in the hospitals of the Board during the year 1896*. (Metropolitan Asylums Board, 1897).
137 Read, M. Recent experiences of Diphtheria. *Public Health* **12**, 346-358 (1900).
138 Tahden, M. *et al.* Epidemiological and Ecological Characterization of the EHEC O104:H4 Outbreak in Hamburg, Germany, 2011. *PLoS One* **11**, e0164508, doi:10.1371/journal.pone.0164508 (2016).
139 Gould, L. H. *et al.* Hemolytic uremic syndrome and death in persons with Escherichia coli O157:H7 infection, foodborne diseases active surveillance network sites, 2000-2006. *Clin Infect Dis* **49**, 1480-1485 (2009).
140 Launders, N. *et al.* Disease severity of Shiga toxin-producing E. coli O157 and factors influencing the development of typical haemolytic uraemic syndrome: a retrospective cohort study, 2009-2012. *BMJ Open* **6**, e009933, doi:10.1136/bmjopen-2015-009933 (2016).
141 Byrne, L., Jenkins, C., Launders, N., Elson, R. & Adak, G. K. The epidemiology, microbiology and clinical impact of Shiga toxin-producing Escherichia coli in England, 2009-2012. *Epidemiol Infect* **143**, 3475-3487 (2015).
142 Burattini, M. N. *et al.* Age and regional differences in clinical presentation and risk of hospitalization for dengue in Brazil, 2000-2014. *Clinics (Sao Paulo)* **71**, 455-463, (2016).
143 Guzman, M. G. *et al.* Effect of age on outcome of secondary dengue 2 infections. *Int J Infect Dis* **6**, 118-124 (2002).
144 WHO Ebola Response Team. Ebola Virus Disease among Male and Female Persons in West Africa. *N Engl J Med* **374**, 96-98 (2016).
145 Aaby, P. Malnutrition and overcrowding/intensive exposure in severe measles infection: review of community studies. *Rev Infect Dis* **10**, 478-491 (1988).
146 Ross, A. H. Modification of chicken pox in family contacts by administration of gamma globulin. *N Engl J Med* **267**, 369-376 (1962).

147 Dunkle, L. M. *et al.* A controlled trial of acyclovir for chickenpox in normal children. *N Engl J Med* **325**, 1539-1544 (1991).
148 Glynn, J. R. & Bradley, D. J. The relationship between infecting dose and severity of disease in reported outbreaks of Salmonella infections. *Epidemiol Infect* **109**, 371-388 (1992).
149 Glynn, J. R., Hornick, R. B., Levine, M. M. & Bradley, D. J. Infecting dose and severity of typhoid: analysis of volunteer data and examination of the influence of the definition of illness used. *Epidemiol Infect* **115**, 23-30 (1995).
150 Glynn, J. R. & Palmer, S. R. Incubation period, severity of disease, and infecting dose: evidence from a Salmonella outbreak. *Am J Epidemiol* **136**, 1369-1377 (1992).
151 Virlogeux, V. *et al.* Brief Report: Incubation Period Duration and Severity of Clinical Disease Following Severe Acute Respiratory Syndrome Coronavirus Infection. *Epidemiology* **26**, 666-669 (2015).
152 Virlogeux, V., Park, M., Wu, J. T. & Cowling, B. J. Association between Severity of MERS-CoV Infection and Incubation Period. *Emerg Infect Dis* **22**, 526-528 (2016).
153 Virlogeux, V. *et al.* Association between the Severity of Influenza A(H7N9) Virus Infections and Length of the Incubation Period. *PLoS One* **11**, e0148506, doi:10.1371/journal.pone.0148506 (2016).
154 Preston, S. H. *Mortality patterns in national populations. With special reference to recorded causes of death*. (Academic Press, 1976).
155 Clark, S. J. & Sharrow, D. J. *Contemporary Model Life Tables for Developed Countries An Application of Model-based Clustering. Working Paper no. 107* (Washington, 2011).
156 Ottochian, M. *et al.* Does age matter? The relationship between age and mortality in penetrating trauma. *Injury* **40**, 354-357 (2009).
157 Petersen, L. K. *et al.* Impact of baseline covariates on the immunogenicity of the 9-valent HPV vaccine - A combined analysis of five phase III clinical trials. *Papillomavirus Res* **3**, 105-115 (2017).
158 Kang, G. *et al.* Comparison of the effect of increased hepatitis B vaccine dosage on immunogenicity in healthy children and adults. *Hum Vaccin Immunother* **12**, 2312-2316 (2016).
159 Tang, Y., Plikaytis, B. D., Preziosi, M. P. & Borrow, R. Influence of Age on Antibody Response and Persistence Following Immunization With MenAfriVac. *Clin Infect Dis* **61 Suppl 5**, S531-539 (2015).
160 Ostergaard, L. *et al.* A Bivalent Meningococcal B Vaccine in Adolescents and Young Adults. *N Engl J Med* **377**, 2349-2362 (2017).
161 Stockdale, L. *et al.* Human cytomegalovirus epidemiology and relationship to tuberculosis and cardiovascular disease risk factors in a rural Ugandan cohort. *PLoS One* **13**, e0192086, doi:10.1371/journal.pone.0192086 (2018).
162 van Lier, A. *et al.* Varicella zoster virus infection occurs at a relatively young age in The Netherlands. *Vaccine* **31**, 5127-5133 (2013).
163 Cohen, D. I. *et al.* Seroepidemiology of Varicella zoster in Israel prior to large-scale use of varicella vaccines. *Infection* **34**, 208-213 (2006).
164 Yoshida, K. *et al.* Aging-related changes in human T-cell repertoire over 20 years delineated by deep sequencing of peripheral T-cell receptors. *Exp Gerontol* **96**, 29-37 (2017).
165 Ben-Smith, A. *et al.* Differences between naive and memory T cell phenotype in Malawian and UK adolescents: a role for Cytomegalovirus? *BMC Infect Dis* **8**, 139, doi:10.1186/1471-2334-8-139 (2008).
166 Miles, D. J. *et al.* Cytomegalovirus infection induces T-cell differentiation without impairing antigen-specific responses in Gambian infants. *Immunology* **124**, 388-400 (2008).
167 Carr, E. J. *et al.* The cellular composition of the human immune system is shaped by age and cohabitation. *Nat Immunol* **17**, 461-468 (2016).
168 Boyd, E. Weight of the thymus and its component parts and number of Hassall corpuscles in health and in disease. *Am J Dis Child* **51**, 313-335 (1936).

169 Steinmann, G. G., Klaus, B. & Muller-Hermelink, H. K. The involution of the ageing human thymic epithelium is independent of puberty. A morphometric study. *Scand J Immunol* **22**, 563-575 (1985).
170 Katzelnick, L. C. *et al.* Antibody-dependent enhancement of severe dengue disease in humans. *Science* **358**, 929-932 (2017).
171 Roved, J., Westerdahl, H. & Hasselquist, D. Sex differences in immune responses: Hormonal effects, antagonistic selection, and evolutionary consequences. *Horm Behav* **88**, 95-105 (2017).
172 Ghosh, S. & Klein, R. S. Sex Drives Dimorphic Immune Responses to Viral Infections. *J Immunol* **198**, 1782-1790 (2017).
173 Giefing-Kroll, C., Berger, P., Lepperdinger, G. & Grubeck-Loebenstein, B. How sex and age affect immune responses, susceptibility to infections, and response to vaccination. *Aging Cell* **14**, 309-321 (2015).
174 Bouman, A., Heineman, M. J. & Faas, M. M. Sex hormones and the immune response in humans. *Hum Reprod Update* **11**, 411-423 (2005).
175 Klein, S. L. & Flanagan, K. L. Sex differences in immune responses. *Nat Rev Immunol* **16**, 626-638 (2016).
176 Van Damme, P. *et al.* A phase III clinical study to compare the immunogenicity and safety of the 9-valent and quadrivalent HPV vaccines in men. *Vaccine* **34**, 4205-4212 (2016).
177 Nagelkerke, N. J. D. *Courtesans and consumption: how sexually transmitted infection drive tuberculosis epidemics*. (Eburon, 2012).
178 Turner, J. E. *et al.* Rudimentary signs of immunosenescence in Cytomegalovirus-seropositive healthy young adults. *Age (Dordr)* **36**, 287-297 (2014).
179 Kaczorowski, K. J. *et al.* Continuous immunotypes describe human immune variation and predict diverse responses. *Proc Natl Acad Sci U S A* **114**, E6097-E6106, doi:10.1073/pnas.1705065114 (2017).
180 Aiello, A. E., Chiu, Y. L. & Frasca, D. How does cytomegalovirus factor into diseases of aging and vaccine responses, and by what mechanisms? *Geroscience* **39**, 261-271 (2017).
181 Nikolich-Zugich, J., Goodrum, F., Knox, K. & Smithey, M. J. Known unknowns: how might the persistent herpesvirome shape immunity and aging? *Curr Opin Immunol* **48**, 23-30 (2017).
182 Savva, G. M. *et al.* Cytomegalovirus infection is associated with increased mortality in the older population. *Aging Cell* **12**, 381-387 (2013).
183 Simanek, A. M. *et al.* Seropositivity to cytomegalovirus, inflammation, all-cause and cardiovascular disease-related mortality in the United States. *PLoS One* **6**, e16103, doi:10.1371/journal.pone.0016103 (2011).
184 Furman, D. *et al.* Cytomegalovirus infection enhances the immune response to influenza. *Sci Transl Med* **7**, 281ra243, doi:10.1126/scitranslmed.aaa2293 (2015).
185 Barton, E. S. *et al.* Herpesvirus latency confers symbiotic protection from bacterial infection. *Nature* **447**, 326-329 (2007).
186 Pera, A. *et al.* CMV latent infection improves CD8+ T response to SEB due to expansion of polyfunctional CD57+ cells in young individuals. *PLoS One* **9**, e88538, doi:10.1371/journal.pone.0088538 (2014).
187 Davis, M. M. & Brodin, P. Rebooting Human Immunology. *Annu Rev Immunol* **36**, 843-864 (2018).
188 Redeker, A. *et al.* The Contribution of Cytomegalovirus Infection to Immune Senescence Is Set by the Infectious Dose. *Front Immunol* **8**, 1953, doi:10.3389/fimmu.2017.01953 (2017).
189 Shanley, D. P., Aw, D., Manley, N. R. & Palmer, D. B. An evolutionary perspective on the mechanisms of immunosenescence. *Trends Immunol* **30**, 374-381 (2009).
190 McDade, T. W., Georgiev, A. V. & Kuzawa, C. W. Trade-offs between acquired and innate immune defenses in humans. *Evol Med Public Health*, **2016**, 1-16 (2016).
191 Shattuck-Heidorn, H., Reiches, M. W., Prentice, A. M., Moore, S. E. & Ellison, P. T. Energetics and the immune system. Trade-offs associated with non-acute levels of CRP in adolescent Gambian girls. *Evol Med Public Health*, 27-38 doi:10.1093/emph/eow1034 (2017).

192 Viner, R. M. *et al.* Susceptibility to SARS-CoV-2 infection amongst children and adolescents compared with adults: a systematic review and meta-analysis Preprint at https://www.medrxiv.org/content/10.1101/2020.05.20.20108126v2 (2020).
193 Miller, E. & Gay, N. Effect of age on outcome and epidemiology of infectious diseases. *Biologicals* **25**, 137-142 (1997).
194 Joachim, R. B. *et al.* The relative resistance of children to sepsis mortality: from pathways to drug candidates. *Mol Syst Biol* **14**, e7998, doi:10.15252/msb.20177998 (2018).
195 Glynn JR. Systematic analysis of infectious disease outcomes by age. *London School of Hygiene & Tropical Medicine* https://doi.org/10.17037/DATA.00001787 (2020)
